# Solvent‐Triggered Aggregation‐Induced Reversal and Enhancement of Circularly Polarized Luminescence in Chiral Salen Metalla‐Macrocycles

**DOI:** 10.1002/smll.202500751

**Published:** 2025-05-09

**Authors:** Qian‐Qian Yan, Jacopo Tessarolo, Shota Hasegawa, Zi‐Yi Han, Elie Benchimol, Alexander S. Mikherdov, Christoph Drechsler, Julian J. Holstein, Yen‐Ting Chen, Sudhakar Ganta, Guido H. Clever

**Affiliations:** ^1^ Department of Chemistry and Chemical Biology TU Dortmund University Otto‐Hahn‐Str. 6 44227 Dortmund Germany; ^2^ Department of Chemistry Chonnam National University 77, Yongbong‐ro, Buk‐gu Gwangju 61186 Republic of Korea; ^3^ Center of Molecular Spectroscopy and Simulation of Solvent‐driven Processes (ZEMOS) Ruhr‐University Bochum 44801 Bochum Germany

**Keywords:** aggregation‐induced emission, chirality, circularly polarized luminescence, macrocycles, self‐assembly, tetraphenylethylenes

## Abstract

Self‐assembled metalla‐macrocycles can serve as versatile platforms to prepare functional materials. Combined with a predictable structural design, they allow for the embedding of a broad range of properties. A series of dinuclear rings **M_2_R** (M = Zn(II), Co(II/III), R = bis‐salen macrocycle) is reported, which combine chirality with aggregation‐induced emission (AIE) and chiroptical sign reversal. The modular system forms from three building blocks: i) tetraphenylethylene (TPE) backbones, ii) chiral salen coordination environments, and iii) chelated transition metal ions. The chiroptical properties are modulated by the choice of metal ion, solvent, and the degree of aggregation, with growing intermolecular stacking leading to an increase of the emission intensity. Aggregation of the macrocycles leads to intensification and inversion of the circular dichroism (CD) signal, and, for **Zn_2_R**, of the circularly polarized luminescence (CPL), with |*g*_lum_| rising by one order of magnitude. The metalla‐macrocycles are characterized by NMR, FT‐IR, and ESI‐MS methods and three single‐crystal X‐ray structures. Dynamic light scattering (DLS), scanning electron microscopy (SEM), and computations are employed to examine the aggregates, showing helically twisted fibers whose handedness is controlled by the chiral component. Gaining stimuli‐responsive control over chiroptical properties contributes to new opportunities for the development of smart optical materials and sensors.

## Graphical abstract

A series of dinuclear metalla‐macrocycles **M_2_R** (M = Zn(II), Co(II/III)) combines a tetraphenylethylene backbone, showing aggregation‐induced emission (AIE), with a chiral salen coordination environment. The system's chiroptical properties depend on the choice of metal ion, solvent, and degree of aggregation. Solvent‐triggered assembly of helical fibers leads to enhancement and sign inversion of circular dichroism and circularly polarized luminescence.

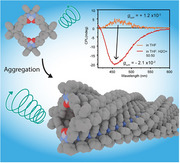

## 1 Introduction

In the past decades, coordination‐driven self‐assembly of polytopic ligands has yielded a wide variety of discrete supramolecular 2D and 3D architectures.^[^ ^1^, ^2^, ^3^, ^4^, ^5^ ^]^ Taking advantage of the directional and defined coordination geometry of most transition metal ions and the fixed bonding vectors of rigid organic ligands, a large number of metalla‐macrocycles^[^ ^6^, ^7^, ^8^, ^9^, ^10^ ^]^ and cages^[^ ^11^, ^12^, ^13^, ^14^, ^15^, ^16^ ^]^ with well‐defined shapes and sizes was developed. Besides studying such compounds as discrete objects, they were also examined with respect to aggregation phenomena in various solvent media.^[^ ^17^, ^18^, ^19^, ^20^, ^21^, ^22^, ^23^, ^24^, ^25^, ^26^ ^]^ Equipped with specific functionalities, a variety of applications in fields such as host‐guest chemistry, molecular sensing, catalysis, and functional materials have emerged.^[^ ^27^, ^28^, ^29^, ^30^, ^31^, ^32^, ^33^ ^]^ In particular, luminescent metalla‐macrocycles have been studied intensively, as they combine emissive properties with an accessible cavity and controllable stacking behavior and may be equipped with stimuli‐responsive photophysical functions.^[^ ^34^, ^35^ ^]^ Flat, conjugated rings that are emissive in dilute solution often show close intermolecular π‐π stacking at higher concentrations and in the solid state, leading to aggregation‐caused quenching (ACQ). This can be overcome by incorporating propeller‐like molecular features whose restriction of intramolecular rotation, hence blocking of non‐radiative deactivation pathways, leads to aggregation‐induced emission (AIE).^[^ ^36^ ^]^ Aggregation can be promoted by increasing concentration in solution, formation of crystals or glasses or by embedding such compounds in polymeric matrices. Alternatively, addition of a bad solvent to a solution of the macrocycle can promote colloidal aggregation, a method that is used in this study.

Common AIE chromophores that have been incorporated in supramolecular complexes include substituted hexaphenylsiloles (HPS) and tetraphenylethylenes (TPE).^[^ ^36^, ^37^ ^]^ In particular TPE derivatives have been widely investigated in photoelectric materials, chemosensors, and bioimaging applications.^[^ ^38^, ^39^ ^]^ Their incorporation into metal‐mediated macrocycles and cages has been shown to block phenyl rotation via steric congestion, resulting in strong fluorescence.^[^ ^40^, ^41^, ^42^, ^43^ ^]^ For example, Stang and co‐workers included TPE moieties in various Pt‐based metalla‐macrocycles^[^ ^44^ ^]^ and cages^[^ ^42^, ^45^ ^]^ and used them as luminescent temperature, pressure and halide sensors, white‐light emitters, and as light‐harvesting systems in photocatalytic reactions.^[^ ^35^ ^]^ Recently, the combination of AIE with chirality has attracted growing attention as it allows to control chiroptical material properties, particularly in terms of circularly polarized luminescence (CPL).^[^ ^46^, ^47^, ^48^ ^]^ Together with an enhancement of the emission properties, formation of aggregates has been shown to significantly enhance the CPL dissymmetry factor *g_lum_* = 2(*I*_L_ – *I*_R_)/(*I*_L_ + *I*_R_) = 2ΔI/I, where *I*_L_ and *I*_R_ represent the emission intensities of left and right circularly polarized luminescence, respectively.^[^ ^49^, ^50^ ^]^ Introduction of chirality into materials often requires considerable synthetic effort. Examples for chiral TPE derivatives have been reported by Zheng et. al., fixing the conformation of TPE by macrocyclization,^[^ ^51^ ^]^ and by Cao et. al. who prepared chiral organic cage by restricting the *P‐* or *M‐*rotational configuration of TPE faces through dynamic covalent bonds,^[^ ^52^ ^]^ and incorporated a TPE backbone in a P_2_L_4_ cage.^[^ ^53^ ^]^

**Figure 1:**
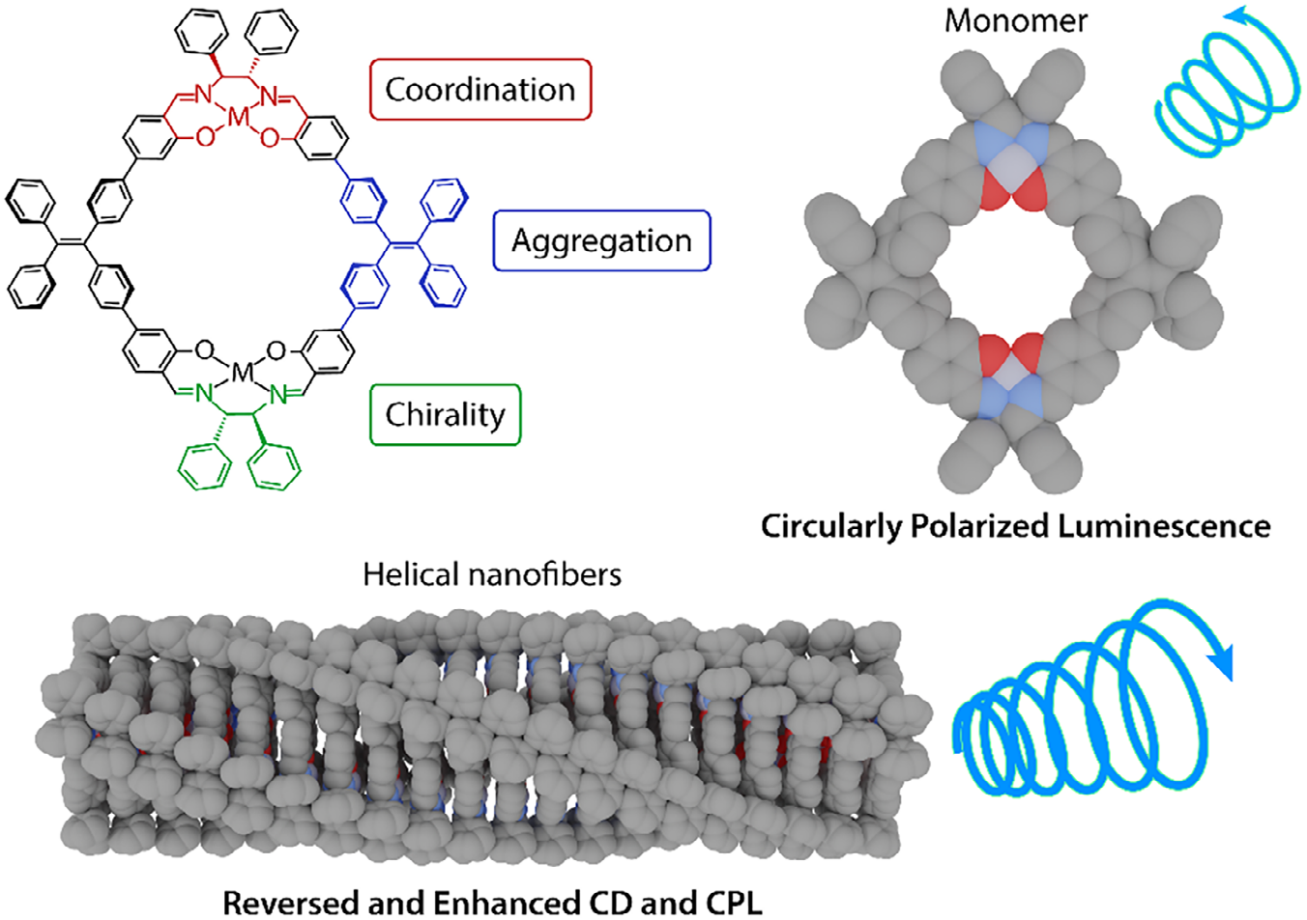
Modular combination of emissive TPE backbones with aggregation tendency, chiral building blocks, and salen coordination environments in **M_2_R** metalla‐macrocycles. Together, these molecular features lead to a system in which chiroptical properties can be reversed and enhanced upon solvent‐induced colloidal aggregation of individual rings to helical nanofibers.

Herein, we demonstrate how modular self‐assembly can be used to rapidly obtain a series of metalla‐macrocycles based on a TPE backbone and a chiral metal‐salen coordination unit. This strategy offers the advantage of easily combining the AIE properties of one building block with the chirality of another moiety, using abundant Zn(II) and Co(II/III) cations instead of the commonly used precious Pt(II) as metal components. The chiral macrocycles are assembled by the Schiff‐base condensation reaction of a TPE‐based bifunctional salicylaldehyde component **L** and an enantiomerically pure chiral diamine, followed by metal complexation. The chiroptical properties and AIE effect of the assemblies were studied and compared for metal‐free and Zn(II)‐containing macrocycles. Interestingly, inducing colloidal aggregation^[^ ^54^ ^]^ by addition of water to THF solutions^[^ ^55^ ^]^ of the metalla‐macrocycles not only caused CD and CPL signal enhancement but complete band reversal deriving from the formation of supramolecular helices (**Figure** **1**), that, to the best of our knowledge, has rarely been reported for similar systems.^[^ ^56^, ^57^, ^58^, ^59^ ^]^

## 2 Results and Discussion

### 2.1 Synthesis and Characterization of M_2_R Metalla‐Macrocycles

**Figure 2:**
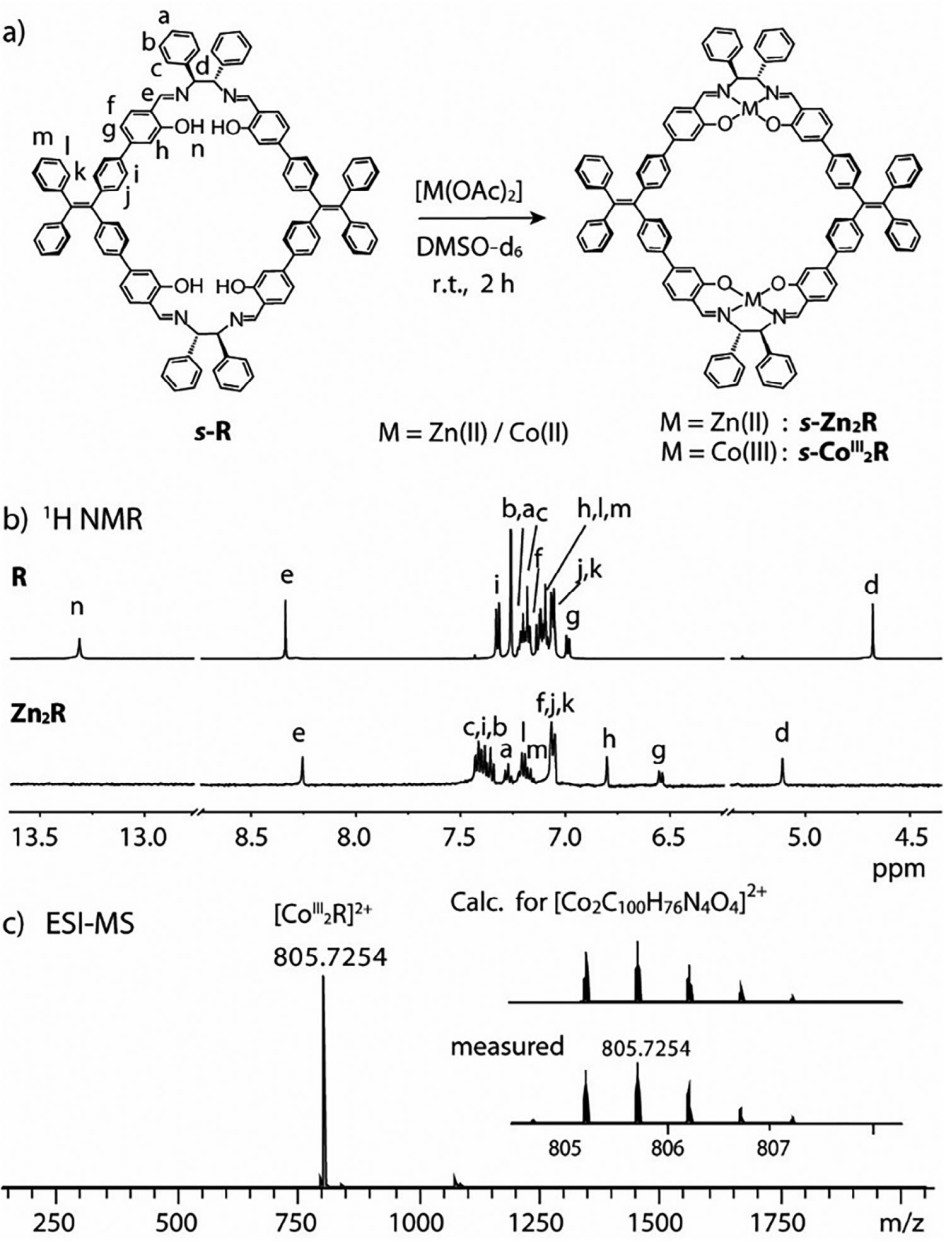
a) Assembly of **M_2_R** metalla‐macrocycles (M = Zn(II), Co(II/III)); b) ^1^H NMR spectra of **R** in CDCl_3_ and **Zn_2_R** in DMSO‐*d*_6_ (600 MHz, 298 K); c) ESI‐MS spectrum of **Co^III^_2_R** with isotopic pattern shown in the inset.

Salicylaldehyde ligand **L** was synthesized by Suzuki‐coupling of 2,2′‐dibromo‐tetraphenylethylene and 4‐formyl‐3‐hydroxyphenylboronic acid pinacol ester (see SI for details). The chiral bis‐salen ring (***r‐***/***s***‐**R**) was prepared by Schiff‐base condensation of the difunctional salicylaldehyde **L** and enantiopure 1,2‐diphenylethylenediamine in quantitative yield (Figure S1, Supporting Information). As a control, the achiral ring **R’** was obtained following the same procedure, but replacing the chiral diamine with ethylenediamine (Figure S2, Supporting Information). The resulting organic macrocycles featuring two salen coordination environments can coordinate a wide range of metal ions.^[^ ^60^, ^61^ ^]^ In this work, we reacted macrocycle ***r‐***/***s***‐**R** (or **R’**), with [M(OAc)_2_] (M = Zn(II), Co(II)) in DMSO in a 1:2 ratio, resulting in the formation of a series of **M_2_R** salen‐based metalla‐macrocycles in quantitative yield (**Figure** **2**a, see Supporting Information for details). Notably, when starting from Co(II) cations, these are easily converted into Co(III) by oxidation with atmospheric oxygen, and NH_4_PF_6_ was added to provide suitable counter anions to balance the overall charge, forming compounds [Co_2_R](PF_6_)_2_ (here named **Co^III^_2_R**).

Formation of the **M_2_R** assemblies was firstly confirmed by ^1^H NMR spectroscopy (Figure 2b). In the case of **Zn_2_R**, the characteristic signal of the Schiff base imine proton H*e* (8.34 ppm in **R**) is upfield shifted to 8.25 ppm upon Zn(II) complexation. At the same time, hydroxyl proton H*n* disappears, further supporting the formation of the metal complex. Diffusion‐ordered spectroscopy (DOSY) of **Zn_2_R** confirms the formation of a single species, with a diffusion coefficient of 9.54 × 10^−11^ m^2^/s (relating to a hydrodynamic radius r_H_ = 11.47 Å, in agreement with the macrocycle dimensions from the crystal structure, *vide infra*). The ^1^H NMR spectrum of **Co^III^_2_R** shows a set of broadened signals, hampering full assignment, most probably caused by residual paramagnetic Co(II) cations in the sample (Figure S29, Supporting Information). Nevertheless, the vanishing of proton H*n* again suggests the formation of the metalla‐macrocycle. The composition of **Co^III^_2_R** was further characterized by ESI‐MS analysis (Figure 2c), revealing a single peak at m/z 805.7254, corresponding to the expected dinuclear macrocyclic complex [**Co^III^_2_R**]^2+^.

The systems were further characterized by Fourier transform infrared (FT‐IR) spectroscopy (Figure S38, Supporting Information). **R** displays a characteristic band at 1625 cm^−1^, corresponding to the Schiff‐base C═N bond vibrations. The band at 3030 cm^−1^ is assigned to C─H aromatic ring stretching, while the band at 2856 cm^−1^ is attributed to the OH group vibration. Upon formation of **Zn_2_R** and **Co^III^_2_R**, the peak at 2856 cm^−1^ disappears, further supporting coordination of the metal ions.

**Figure 3:**
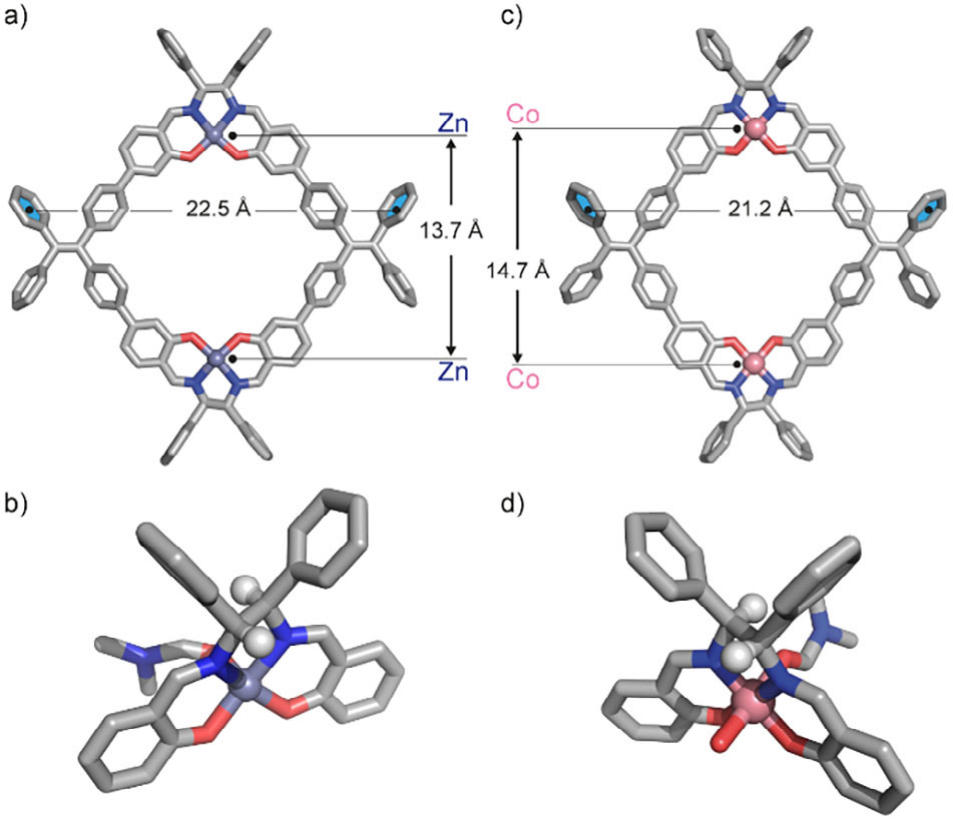
X‐Ray single crystal structure of a) ***r***‐**Zn_2_R** with Zn∙∙∙Zn and TPE∙∙∙TPE distances given; b) highlight of the Zn(II) square‐pyramid coordination environment and the *R*,*R*‐chirality of the two stereogenic sp^3^‐carbon centers; X‐Ray single crystal structure of c) ***s***‐**Co_2_R** with Co∙∙∙Co and TPE∙∙∙TPE distances given and d) highlight of the Co(III) octahedral coordination environment and the *S*,*S*‐chirality of the two stereogenic sp^3^‐carbon centers. Color code for C: gray, N: blue; O: red; Zn: dark purple; Co: Pink; H: white when shown. Solvent molecules and hydrogens are not shown for clarity.

The structures of achiral ring **R’** and homochiral ***s/r‐*M_2_R** metalla‐macrocycles were unambiguously confirmed by single‐crystal X‐ray diffraction analysis (**Figure** **3**). Colorless plates of **R’** were obtained by slow evaporation of CDCl_3_, while red plates of ***s***‐**Co_2_R** and colorless plates of ***r***‐**Zn_2_R** were grown by slow vapor diffusion of diethyl ether into DMF. Enantiopure ***r***‐**Zn_2_R** crystallize in the *C_2_* space group, with half metalla‐macrocycle in the asymmetric unit. The full macrocycle is composed of two propeller‐like TPEs bridged by two salen units (Figure 3a). The enantiopurity distinguishing parameters according to Parsons = 0.152(14) (determined with the SHELX software) and Hooft = 0.142(10) (determined with the PLATON software) were found to be only moderate. Each Zn(II) center shows a square‐pyramid geometry, with the N_2_O_2_ coordination sites of the salen units occupying the plane, while the apical position coordinates a DMF molecule with 50% occupancy (Figure 3b). The Zn∙∙∙Zn distance is 13.662(2) Å. The longest C∙∙∙C distance between the two salen‐units (diagonal from phenyl‐to‐phenyl substituent) measures 26.09(2). In the solid state, the metalla‐macrocycles are packed in columnar stacks with the TPE‐backbones of neighboring columns interdigitating in an alternating fashion, thus restricting the intramolecular rotation of the phenyl substituents. Likewise, the salen units are aligned on top of each other's. Furthermore, porous channels span the columnar assembly in the solid‐state, with a solvent accessible volume corresponding to 27% of the unit cell (determined with the PLATON software with a 1.2 Å probe radius).

Enantiopure ***s***‐**Co_2_R** crystallizes in the *P2_1_* space group and consist of one full metalla‐macrocycle in the asymmetric unit (Figure 3c). In this case the enantiopurity distinguishing parameters were found to be strong (Parsons = 0.104(7) and Hooft = 0.050(5)). Both Co(III) centers have an octahedral coordination geometry, with the salen N and O donors filling the equatorial positions while the apical positions are occupied by two DMF molecules for one metal center, and one DMF and one water molecule for the second one (Figure 3d). Compared to the Zn analogue, in **Co_2_R** the metal‐metal distance is about 1 Å longer (d = 14.658(3) Å). The reason for this observation is that coordination of Co(III) cations in the salen unit gives rise to shorter ligand‐to‐metal bonds compared to Zn(II) ions, thus squeezing the overall ring geometry and resulting in a larger metal‐metal separation, concomitantly with a shorter TPE‐TPE distance, measured from the centroids of the peripheral phenyl rings (22.49 Å for **Zn_2_R**, 21.18 Å for **Co_2_R**).

### 2.2 Photophysical Characterization

**Figure 4:**
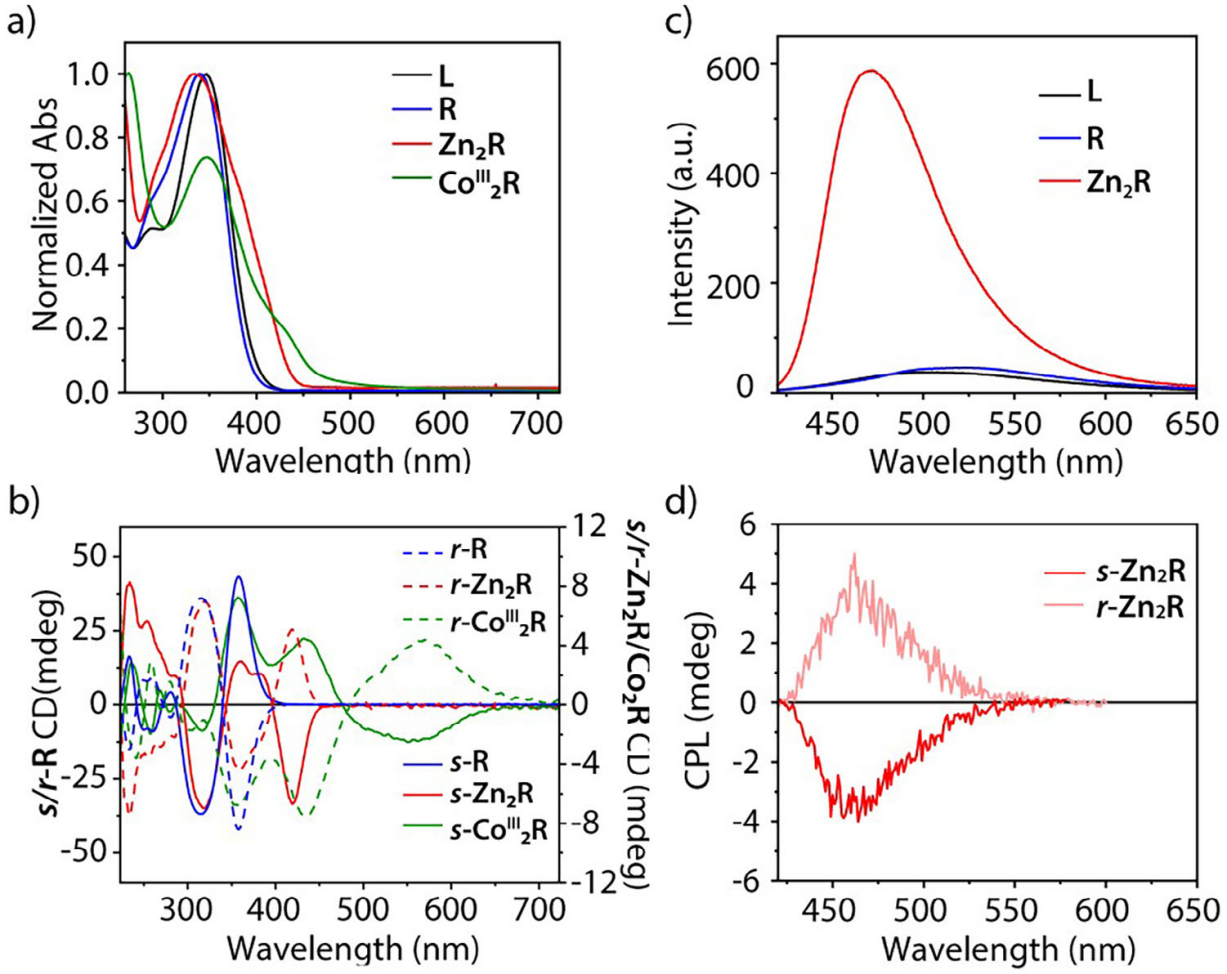
a) Normalized UV–vis absorption spectra of **L**, **R**, **Zn_2_R**, and **Co^III^_2_R**, b) CD spectra of ***s/r***‐**R**, ***s/r*‐Zn_2_R** and ***s/r*‐Co^III^_2_R**, c) emission spectra of **L**, **R** and **Zn_2_R**, and d) CPL spectra of ***s/r*‐Zn_2_R** (all spectra in THF, RT, c**_L_** = 2 × 10^−5^ м, c**_R,Zn2R,Co2R_** = 1 × 10^−5^ M, λ_ex_ = 360 nm).

Next, we studied the photophysical properties of the macrocycles in their monomeric form in dilute THF solution (**Figure** **4**). In the UV‐Vis spectra, ligand **L** shows a broad absorption band for the π‐π* transitions centred at 347 nm with a molar absorption coefficient (ɛ) of 7.53 × 10^4^ M^−1^cm^−1^.^[^ ^62^ ^]^ After forming the macrocyclic structure, the absorption maximum of **R** shifts to 339 nm. Upon coordination of Zn(II) cations, the absorption shows a slight hypsochromic shift compared to free macrocycle **R** with an absorption maximum at 334 nm (*ɛ* = 9.86 × 10^4^ M^−1^cm^−1^) and a shoulder around 400 nm, characteristic for metal complex formation in **Zn_2_R** (Figure 4a).^[^ ^63^ ^]^ Metallacycle **Co^III^_2_R** shows a similar absorption spectrum, with the addition of a shoulder at about 450 nm (Figure 4a). The analogue achiral compounds **R’** and **Zn_2_R’** show a similar behavior (Figure S39b, Supporting Information). All the compounds assembled from the chiral salen moieties, **R**, **Zn_2_R**, and **Co^III^_2_R**, show mirrored circular dichroism bands for the two enantiomers, while achiral ligand **L** and the rings **R’** and **Zn_2_R’** expectedly show no CD effects (Figure S44a, Supporting Information). The absence of linear dichroism (LD) contaminations (caused by some sort of sample anisotropy, e.g. any preferred aggregate orientation in the cuvette) to the measured signals was confirmed by showing that the CD spectra of the samples remain indistinguishable after turning the cuvettes by 180°. For the ***s/r*‐R**, ***s/r*‐Zn_2_R**, and ***s/r*‐ Co^III^_2_R** enantiomeric couples, the chiral information brought in via the diamine components is affecting the overall macrocycle, as evidenced by the clear observation of Cotton effects for the absorption band assigned to the TPE moieties, with maximal ellipticity at around λ = 350 nm (Figure 4b). Moreover, the metalla‐macrocycles show CD signatures ascribed to the metal‐salen transitions, with maximal ellipticity at 410 nm for Zn(II)‐salen, and a broad CD band in the visible range for the Co(III)‐salen moiety, centered at ≈550 nm. The latter may arise from electric dipole–forbidden but magnetic dipole–allowed transitions involving Co(III), which, in the presence of a dissymmetric force field, result in induced rotational strength, even when absorption in this region is relatively weak.^[^ ^64^ ^]^

Next, we investigated the emission properties of the systems in THF. As expected, **L** and **R** show a typical TPE emission band from 400–650 nm after excitation at λ = 360 nm, however, of weak intensity due to the freely rotating phenyl rings (Figure 4c). After coordinating Zn(II) cations in THF solution, **Zn_2_R** emits a strong blue luminescence with a shift of the emission maximum from 525 to 470 nm and a sixfold enhancement of the intensity compared to metal‐free **R** (Figure 4c). The latter is explainable by an increased structural rigidity after metal inclusion,^[^ ^42^, ^65^ ^]^ as well as the suppression of nonradiative n‐π* transitions and a photo‐induced enol‐keto tautomerization in the uncomplexed salen moiety,^[^ ^66^, ^67^ ^]^ both of which can lead to emission quenching and reduced photostability. Emission of **Co^III^_2_R** was found to be completely quenched and thus, this compound was not investigated further.

The chiral transfer from the diamines to the overall assembly, as detected by CD spectroscopy, combined with the TPE emission, endows metalla‐macrocycle **Zn_2_R** with circularly polarized luminescence (CPL) properties. Upon excitation at λ_ex_ = 360 nm, ***s/r*‐Zn_2_R** show mirror CPL behavior, with a maximum at 460 nm, corresponding to the emission from the TPE moiety, and a dissymmetry factor |*g*_lum_| = 1.2 × 10^−3^ in THF (Figure 4d). The *s*‐enantiomer shows a CPL band with negative sign, while ***r*‐Zn_2_R** gives a CPL band with positive sign, in agreement with the CD signature for the lowest‐energy transition in the absorption spectrum (λ_max_ = 410 nm). The obtained CPL data further demonstrates the success of the employed modular strategy to easily assemble discrete macrocycles with promising chiroptical properties, starting from commercially available chiral building blocks.

### 2.3 Aggregation‐Induced Effect on Emission and Chiroptical Properties

**Figure 5:**
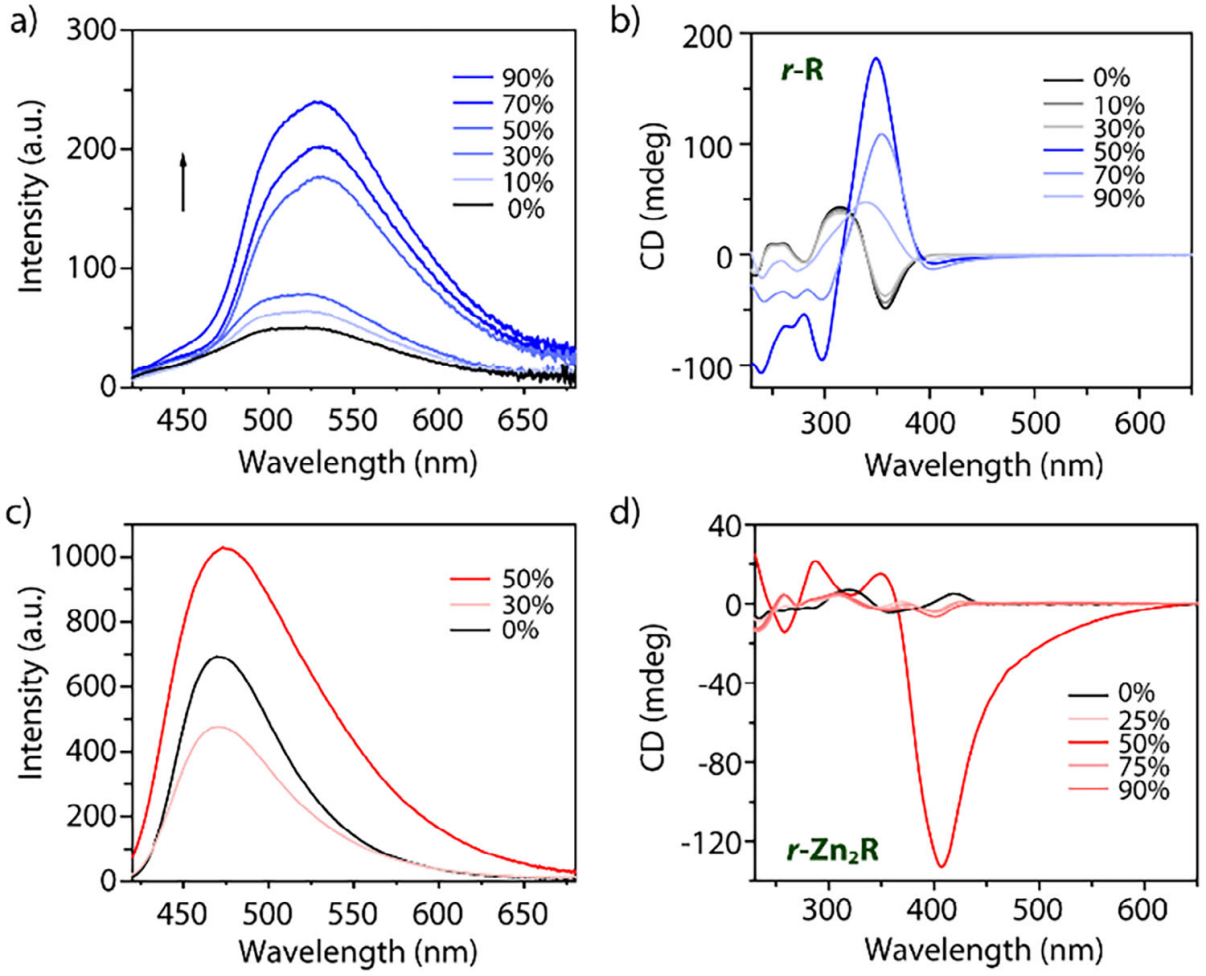
a) Emission spectra of **R**, b) CD spectra of ***r*‐R**, c) emission spectra of **Zn_2_R**, and d) CD spectra of ***r***‐**Zn_2_R** in THF/H_2_O with different fractions of water (c = 1 × 10^−5^ ****M, λ_ex =_ 360 nm). Spectra for the other enantiomers are reported in the Supporting Information (Figure S45, Supporting Information).

By constructing the rings from multiple aromatic moieties, including TPE as the ligand backbone, the macrocyclic compounds are predestined to form supramolecular aggregates of tubular shape. We wondered whether aggregation leads to a tuning of the (chir)optical properties, going along with aggregation‐induced emission (AIE), as well as modification of the CD and CPL effects (**Figure** **5**). This was investigated by exposing the THF solutions of the rings to increasing amounts of water. The emission spectra of metal‐free species **L** and **R** show a similar behavior, with a typical AIE effect arising in the form of a gradual enhancement of emission intensity as the water fraction is increased from 0 up to 90% (Figure S41, Supporting Information; Figure 5a). Furthermore, the emission wavelength shows a blue shift upon water addition, which is in line with the reported TPE‐based AIE mechanism, assuming that the perpendicular conformation of the peripheral phenyl rings weakens π‐conjugation in the chromophore.^[^ ^68^ ^]^

In case of metalla‐macrocycle **Zn_2_R** (Figure 5c; Figure S42b, Supporting Information), the emission decreases upon addition of 10% of water, probably due to polarity‐induced fluorescence quenching. However, after adding 50% water, the fluorescence shows a twofold enhancement, supporting the persistence of the AIE effect. When the water content exceeds 50%, the formation of a precipitate is observed and the emission intensity only negligibly increases. Fluorescence quantum yields (ΦF) both in THF and a THF: H_2_O = 10:90 mixture were measured, showing an increase from the monomeric to the aggregated state (Figure S43 and Table S4, Supporting Information).

Formation of the colloidal aggregates also strongly affects the chiroptical properties of ***s/r*‐R** and ***s/r*‐Zn_2_R** macrocycles, giving rise to i) sign inversion and ii) increase of the amplitudes of both CD and CPL bands, reaching the largest effect with a water fraction of 50% (after adding even more water, precipitation is observed, hence again reducing chiroptical signal intensity, Figures S45–S48, Supporting Information). The CD signal of **R** around 350 nm initially shows a slight intensity decrease with the addition of water. When the water content exceeds 50%, however, the CD signal changes sign and enhances significantly, with *g*_abs_ increasing by a factor of four (Figure 5b; Table S5, Supporting Information). With even more water, the signal intensity decreases again, concomitant with the onset of precipitation, which is also consistent with DLS data indicating smaller particle sizes with higher water content (Figure S51, Supporting Information). Concerning CPL, the structurally flexible ring **R** does not show any measurable effect in pure THF and only a low intensity band in its aggregated form, furthermore suffering from instability under irradiation conditions in the CPL spectrometer (Figures S49 and S50, Supporting Information). As reason for the aggregation‐induced increase in CD and CPL band intensity, we assume formation of higher‐order columnar stacks with a common helical twist,^[^ ^68^ ^]^ controlled by the molecular chirality of the individual rings (see discussion below).

The chiroptical properties of **Zn_2_R** show a similar behavior. The THF solution of **Zn_2_R** becomes turbid when water is added and shows a reversal of the CD signal ≈410 nm (Figure 5d). With 50% water, the signal is dramatically increased, with *g*_abs_ reversing the sign and increasing by one order of magnitude (from 1.4 × 10^−3^ in THF to 2 × 10^−2^ in the THF/water mixture). We hypothesize that the reversal of the chiroptical signal is connected to the formation of a higher‐order aggregate with a pronounced, larger scale helical chirality.^[^ ^69^ ^]^

**Figure 6:**
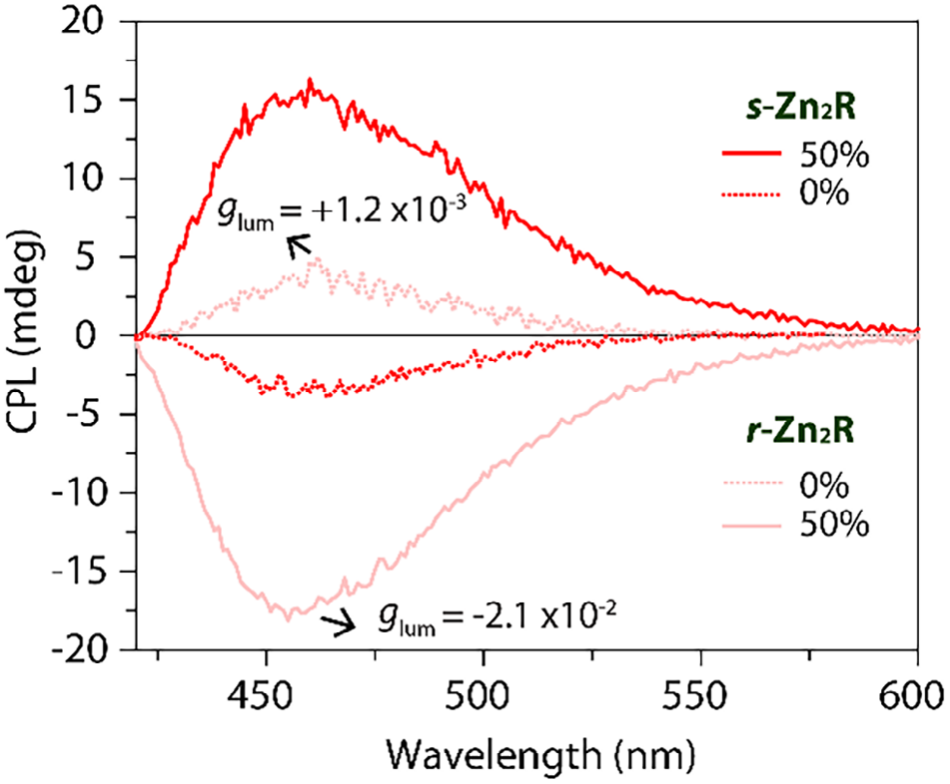
CPL spectra of ***s***‐/***r‐*Zn_2_R** in THF with different amounts of water (c = 1 × 10^−5^ ****M, λ_ex,_ **_Zn2R_** = 370 nm).

In a similar way, also the CPL signal of the ***s/r*‐Zn_2_R** monomers was found to invert and significantly increase when forming the colloidal aggregates (**Figure** **6**). The positive CPL band of ***r*‐Zn_2_R** changes sign upon addition of 50% water, in line with the CD spectroscopic results. In the THF/water mixture, the CPL band is blue‐shifted as compared to the spectrum in THF, in line with the observation in the respective emission spectra. Interestingly, the CPL intensity is enhanced by a factor of 20, going from *g*_lum_ = 1.2 × 10^−3^ in THF to *g*_lum_ = –2.1 × 10^−2^ in the THF/water mixture (1:1 ratio). As expected, analogous results, but with mirror image CPL bands, are obtained with ***s*‐Zn_2_R**.

**Figure 7:**
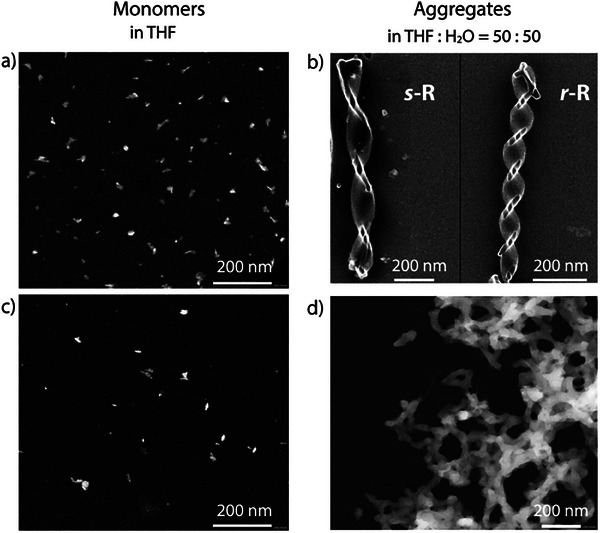
SEM images of a) ***s***‐**R**, c) ***s***‐**Zn_2_R** in THF, b) ***s***‐ (left) and ***r***‐**R** (right), d) ***s***‐**Zn_2_R** in THF**/**H_2_O (50:50, v:v). In both enantiomeric samples of the aggregated rings **R** we found helices of different pitch (but always same handedness), with the degree of twisting seemingly correlated to the fibers’ lateral widths (thinner fibers show stronger twisting, see Figure S55, Supporting Information).

The inversion and increase of the CD and CPL signals upon solvent‐induced aggregation suggest the formation of a higher‐order supramolecular structure. Other solution‐based spectroscopies, such as NMR, did not deliver further insights into the aggregation pattern during water addition (Figures S22–S24, Supporting Information). Therefore, to gain further insight into the morphology of the aggregated species, we performed a scanning electron microscopy (SEM) analysis (**Figure** **7**). At first, ***s***‐/***r‐*R** and ***s***‐/***r‐*Zn_2_R** were dropcasted from pure THF onto a silicon chip surface but images only showed dispersed speckles, without any signs of formation of a long‐range ordered supramolecular structure (Figure 7a). In contrast, SEM images of ***s***‐/***r‐*R** deposited from a THF: H_2_O = 50:50 mixture show the formation of uniform helical fibers with a diameter of about 200 nm–2 µm. Mirror image helices with left‐ and right‐handedness were observed to form from ***s***‐**R** and ***r***‐**R**, respectively (Figure 7b), albeit with a different helical pitch. As a control, achiral **R’** was found to only form non‐helical nanofibers (Figure S54e,f, Supporting Information). The morphology of **Zn_2_R** in the mixed solvent was also examined by SEM (Figure 7d). A mesh of less‐defined and more tightly packed fibers was observed and the existence of Zn in the structure was confirmed by EDS element mapping results, revealing all expected elements, namely C, N, O, and Zn (Figure S57, Supporting Information). In addition, dynamic light scatting (DLS) data for **R** and **Zn_2_R** was collected, using a concentration of 1 × 10^−5^ M in THF/water solution with differing water contents (Figures S51 and S52, Supporting Information). The average sizes of colloidal particles in THF were found to be below 200 nm, increasing up to 800 nm in a THF: H_2_O = 50:50 mixture for both **R** and **Zn_2_R** species.

### 2.4 Computational Study

In order to understand both the chiroptical behavior of the THF‐solvated and aggregated rings as well as the relation between plausible molecular models of the aggregated rings assembled into columnar stacks, we performed a series of computations.

**Figure 8:**
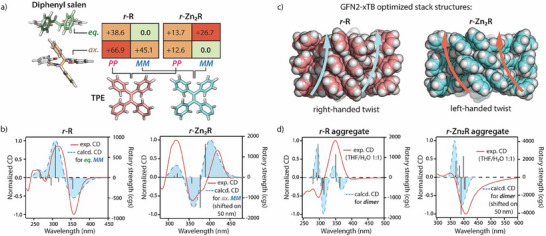
a) Relative energies of ***r*‐Zn_2_R** and ***r*‐R** conformers (kJ/mol; ωB97M‐V/def2‐TZVP/CPCM(THF)). b) Overlay of experimental (THF) and computed (BHandHLYP/def2‐SVP/CPCM(THF)) CD spectra for the lowest energy conformers of ***r*‐R** (eq. MM) and ***r*‐Zn_2_R** (ax. MM) monomers. c) GFN2‐xTB computed structures of stacks of three flat rings of ***r*‐R** (left) and of ***r*‐Zn_2_R** (right), displaying opposite helical assembly directions. d) Overlay of experimental (THF/H_2_O 1:1) and computed (BHandHLYP/def2‐SVP level) CD spectra for the aggregates of ***r*‐R** and ***r*‐Zn_2_R** (dimers).

First, we examined the conformational preference of the Zn‐free and Zn‐coordinated rings with *r,r*‐ (or *s,s*‐) stereochemistry at the diphenyl‐ethylenediamine parts by geometry‐optimizations (ORCA^[^ ^70^ ^]^ r^2^SCAN‐3c,^[^ ^71^ ^]^ followed by single point energies on ωB97M‐V/def2‐TZVP level of theory in CPCM solvent THF; for details see Supporting Information). For **Zn_2_R**, the lowest energy structure in solution always carries all phenyl substituents in axial positions (**Figure** **8**a; Table S6, Supporting Information). For the more structurally flexible metal‐free ring **R**, however, the equatorial conformer was found to be more stable due to the bent ring structure (Table S6, Supporting Information). Moreover, according to the calculations, the chirality and conformation of the salen moiety clearly affect the structure of the TPE units by a mechanical relay effect, conveyed by the 1,4‐phenylene linkers. This leads to the stabilization of a specific chiral conformational pattern via intramolecular chirality transfer, resulting in an *MM*‐conformation for *r,r*‐rings and *PP*‐conformation for *s,s*‐rings (Table S6, Supporting Information).

Next, we computed for all conformers of metal‐free ring ***r*‐R** and ***r***‐/**s‐Zn_2_R** the CD spectra in CPCM solvent THF by TD‐DFT calculations on the BHandHLYP/def2‐SVP level of theory. Interestingly, according to the calculations results, the sign of the longest wavelength CD band (Cotton effect) is governed by the eq./ax. conformation of the diphenyl salen units, not by the *P*‐ or *M*‐conformation of TPE moeties (Figure S59, Supporting Information). Moreover, for the lowest energy conformers of ***r*‐R** (eq. MM) and ***r*‐Zn_2_R** (ax. MM), the computed CD band signs are in full agreement with the experimentally observed CD signatures of these samples in THF solution (Figure 8b).

In order to get insight into plausible molecular arrangements in the aggregates obtained from the THF:water = 1:1 mixtures, we stacked three flat rings of metal‐free ***r*‐R** and of ***r*‐Zn_2_R**, respectively, in a way that the TPE‐units are on top of each other (likewise the salen units). While the conducted DFT calculations suggest that the axial conformation is more favorable for **Zn_2_R** in THF solution, the analysis of both obtained X‐ray structures for **Zn_2_R** and **Co^III^_2_R**, as well as available data from the Cambridge Structural Database (CSD) for salen complexes (Figure S60, Supporting Information), indicate that the equatorial conformation is the most common and probable arrangement for the diphenyl ethylenediamine unit in salen species in the solid and aggregated phases. This conformation should also allow for a much closer arrangement of the salen units and promote extensive π‐stacking interactions between the aromatic components in the stacked structures. Additionally, DFT calculations indicate that the energy required for the axial‐to‐equatorial conformational change in **Zn_2_R** ring can be fully compensated by the energetic gain from stacking of the rings (Table S6, Supporting Information). Therefore, for both stacks of metal‐free ***r*‐R** and of ***r*‐Zn_2_R**, the phenyl substituents on the ethylene diamine parts were arranged in equatorial positions and the trimeric stacks were then optimized using the GFN2‐xTB^[^ ^72^ ^]^ method. In both cases, the gearing of the aromatic substituents within the chiral rings led to an overall helical twist of the aggregates along the stacking axis. Interestingly, the optimized stack of the metal‐free, structurally flexible ***r*‐R** formed a *right*‐handed helix, while the more rigid, metal‐containing ***r*‐Zn_2_R** rings formed a *left*‐handed helical stack. This observation also aligns with the CD data of ***r*‐R** and ***r*‐Zn_2_R** measured after the water‐induced aggregation process, indicating opposite handedness of the formed aggregates. Additionally, CD calculations (BHandHLYP/def2‐SVP) for the optimized dimeric stacks of ***r*‐R** and ***r*‐Zn_2_R** further confirm an inversion of the sign of the CD band compared to the THF solution (Figure 8d). In the case of ***r*‐Zn_2_R**, this CD sign inversion is caused by the conformational change of the salen unit from axial to equatorial in the stack, as well as the *left*‐handed helical arrangement. For ***r*‐R**, this effect is primarily attributed to the *right*‐handedness of the stack.^[^ ^73^, ^74^ ^]^

From the experimental data, both metal‐free and Zn(II)‐containing rings exhibit similar behavior upon aggregation, showing a maximum enhancement of the CD response at 50% water content, as well as forming aggregates of similar size, according to DLS measurements. While the handedness of the **Zn_2_R** aggregates could not be resolved by SEM, in the case of ***r*‐R**, the *right*‐handed helical fiber observed by SEM corresponds to the *right*‐handedness of the calculated stacks, indicating a chirality transfer from the stack to the fiber. A similar relationship between the handedness of the stacked structure and the fiber was previously observed for stacked aggregates of other macrocyclic systems, such as chiral porphyrin rings,^[^ ^75^ ^]^ rosette‐like cyclic aggregates,^[^ ^76^ ^]^ or Ag(I)‐coordination macrocycles.^[^ ^77^ ^]^

## 3 Conclusion

We report the modular synthesis and characterization of a series of TPE‐based chiral dinuclear salen metalla‐macrocycles, combining aggregation‐induced emission (AIE) properties (contributed by the TPE chromophores) with chirality (introduced by diphenyl substituted salen units) and structural rigidity (established by metal coordination). Coordination of organic macrocycle **R** by two Zn(II) cations, forming **Zn_2_R**, leads to an increase of the emission properties both for the THF solution of the metalla‐macrocycles as well as for colloidal aggregates formed by the addition of water to the THF solution of the rings. CD and CPL spectra clearly show a chirality transfer from the two salen coordination environments to the TPE chromophores. Long‐range helical chirality is manifested in the aggregates, as characterized by chiroptical spectroscopy and SEM imaging. Metalla‐macrocycle ***s/r***‐**Zn_2_R** shows a reversal of the CD as well as CPL signal signs in the aggregated state with the |*g*_lum_| dissymmetry factor increasing from 1.2 × 10^−3^ to 2.1 × 10^−2^. Computations revealing the lowest energy conformers of the non‐metalated rings **R** as well as Zn(II) complexes **Zn_2_R** deliver calculated CD spectra in good agreement with the experimental results, indicating that the equatorial/axial position of the salen phenyl substituents governs the CD band sign for the fully solvated rings. Upon water‐induced aggregation, models of stacked rings suggest that both ***r*‐R** and ***r*‐Zn_2_R** form extended fibres of opposite helicity, in accordance with opposite CD signatures measured for the colloidal samples. The handedness of the modelled stack of **R** agrees with the handedness observed in the corresponding SEM images. Noteworthy, according to the calculations results, rings **R** keep their equatorial conformation upon aggregation while rings **Zn_2_R** flip from their axial solution preference to flatter equatorial in the π‐stacked aggregates. We assume that the experimentally observed CD sign change observed for both ***r*‐R** and ***r*‐Zn_2_R** upon water‐induced aggregation is then dominated by the overall helicity of the entire polymer stacks (similar to what is observed in helically assembled biopolymers such as double‐stranded or G‐quadruplex DNA).^[^ ^78^, ^79^ ^]^

The employed modular approach can be used to incorporate multiple properties in a metallosupramolecular self‐assembly with low synthetic effort.^[^ ^80^ ^]^ Obtained properties result from a synergistic interaction of the assembled building blocks, allowing to form material libraries featuring strong emission and adjustable chiroptical properties with potential application in chiral receptors, photo‐redox systems, CPL‐based displays and optoelectronic devices.

## Conflict of Interest

The authors declare no conflict of interest.

## Supporting information



Supporting Information

Supporting Information

Supporting Information

Supporting Information

## Data Availability

The data that support the findings of this study are available in the supplementary material of this article.
