# Supplementary material for: Solvent‐Triggered Aggregation‐Induced Reversal and Enhancement of Circularly Polarized Luminescence in Chiral Salen Metalla‐Macrocycles
Source: Small. 2025 May 9;21(37):2500751. doi: 10.1002/smll.202500751 (PMC12444834; doi:10.1002/smll.202500751)
Supplement: Supplementary file 1 — Supporting Information [file SMLL-21-2500751-s004.pdf]

NANO · MICRO
small

Supporting Information

for *Small*, DOI 10.1002/smll.202500751

Solvent-Triggered Aggregation-Induced Reversal and Enhancement of Circularly Polarized Luminescence in Chiral Salen Metalla-Macrocycles

Qian-Qian Yan, Jacopo Tessarolo, Shota Hasegawa, Zi-Yi Han, Elie Benchimol, Alexander S. Mikherdov, Christoph Drechsler, Julian J. Holstein, Yen-Ting Chen, Sudhakar Ganta and Guido H. Clever**

Contents

1. Materials and methods ... 2
2. Experimental Procedures... 2
2.1 Synthesis of pinacol ester salicylaldehyde compound 1: ... 3
2.2 Synthesis of compound 2: ... 3
2.3 Synthesis of L:... 3
2.4 Synthesis of *s*-R: ... 3
2.5 Synthesis of R’: ... 4
2.6 Synthesis of M_2R and Zn_2R’: ... 4
3. NMR spectroscopic results ... 6
3.1 ^{1}H DOSY NMR investigation...17
4. Further ESI mass spectrometric results ...19
5. X-ray crystal structure analysis...20
6. FT-IR spectroscopy ...25
7. UV-Vis Absorption spectroscopy ...25
8. Emission spectroscopy ...26
8.1 Quantum yield (QY) determination...28
9. Chiroptical Spectroscopy ...29
9.1 Circular Dichroism (CD) Spectra...29
9.2 Circularly Polarized Luminescence (CPL)...31
10. Dynamic Light Scattering (DLS) experiments ...33
11. SEM and EDS Electron Microscopy Results...34
12. Theoretical Calculations ...38
13. References...45

1. Materials and methods

Unless otherwise stated, all chemicals and solvents were purchased from commercial companies (Abcr, Sigma Aldrich, Acros Organics, VWR and Chempur) and used as received. (1*S*,2*S*)-1,2-diphenylethane-1,2-diamine and (1*R*,2*R*)-1,2-diphenylethane-1,2-diamine were recrystallized from hexane. NMR spectroscopic data was measured on the spectrometers Bruker AV 500 Avance NEO and Bruker AV 600 Avance III HD. Chemical shifts for ^{1}H and ^{13}C spectra are reported in ppm on the δ scale relative to proton resonance resulting from incomplete deuteration of the solvents. The chemical shift δ is given in ppm, the coupling constants J in Hz. High resolution Electrospray Ionization (HR-ESI) mass spectra and trapped ion mobility data were recorded on Bruker ESI-timsTOF and Compact mass spectrometers. Fourier Transform Infrared (FT-IR) spectra were measured on Perkin Elmer Spectrum Two spectrometer in the 400-4000 cm^{-1} spectral region. UV-vis spectra were recorded on a DAD HP-8453 UV-Vis spectrometer. Circular dichroism spectra were recorded in THF/water with an Applied Photophysics Chirascan qCD Spectrometer with a temperature-controlled cuvette holder. The spectra were background-corrected and smoothed with a window size of 5. Emission measurements were performed on a JASCO FP-8300 spectrometer, quantum yield determination have been performed on a JASCO ILF-835 integrating sphere as accessory of the JASCO FP-8300 and circularly polarized luminescence measurements were performed using a JASCO CPL-300 spectrophotometer, both equipped with a (150 W) Xe lamp as light source. CPL spectra are recorded with excitation bandwidth of 15 nm and emission bandwidth of 15 nm and averaged over 20 spectra (1 nm steps, 1 s digital integration time). Dynamic light scattering experiments were performed on a Malvern Zetasizer ZS nano instrument, with a single 173° scattering angle, and at 25 °C temperature. SEM measurements were done using a JEOL JEM-2800 with Schottky field emission cathode operated at 200 kV. SEM detectors are equipped for SEM images. Dual SDD X-ray detectors are used to capture EDS signals for elemental mapping with solid angle of 0.95 srad, and with 133 eV of spectral resolution.

2. Experimental Procedures

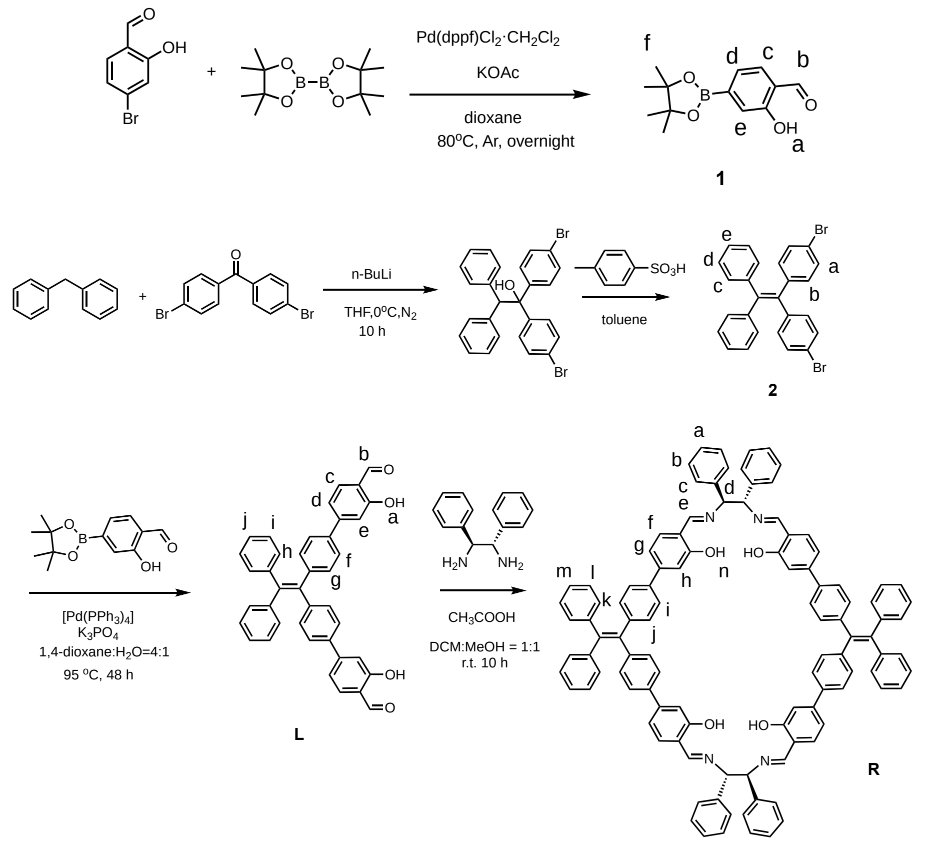

Figure S1. Synthesis of chiral macrocycle **R**.

2.1 Synthesis of pinacol ester salicylaldehyde compound 1:

The synthesis of **1** was carried out according to the literature procedure.[1]
Therefore, 4-bromosalicylaldehyde (1.0 g, 4.97 mmol), Pin_2B_2 (1.52g, 5.97 mmol), Pd(dppf)Cl_2·CH_2Cl_2 (203 mg, 0.249 mmol), and KOAc (1.46 g, 14.92 mmol) were combined in a 100 mL Schlenk tube, 15 mL anhydrous 1,4-dioxane were added. The mixture was degassed via freeze-thaw cycles and then heated at 85 ^oC under N_2 atmosphere for 5 h. After cooling to room temperature, the solvent was removed under vacuum. The residue was dissolved in CH_2Cl_2 and washed with H_2O and dried over $MgSO_4$. The crude product was purified by column chromatography (dichloromethane: pentane = 1:1) to provide a white solid as aim product (0.92 g, yield 75%). ^1H NMR (600 MHz, $CDCl_3$, 298 K): δ 10.83 (s, 1H, Ha), 9.93 (s, 1H, Hb), 7.54 (s, 1H, Hc), 7.42 (d, *J* = 8.4 Hz, 2H, Hd,He), 1.35 (s, 12H, Hf). ^{13}C NMR (151 MHz, $CDCl_3$): δ 197.16, 160.94, 132.96, 125.72, 124.20, 122.36, 84.78, 25.21.

2.2 Synthesis of compound 2:

2 was synthesized in two steps by following a reported procedure.[2]
Diphenylmethane (2.0 g, 11.9 mmol) and anhydrous tetrahydrofuran 15 mL were mixed in a 100 mL Schlenk tube and stirred under argon at 0°. A 2.5 M solution of *n*-butyllithium (4.8 mL, 762 mg, 12.88 mmol) in hexane was added into the Schlenk tube. After stirring for 1 h at 0°, 4-(4-bromophenyl) benzophenone (3.24 g, 9.5 mmol) was added and the reaction mixture was stirred for 10 h and the temperature to rise gradually to room temperature. Then the reaction was quenched with NH_4Cl aq. The mixture was extracted with DCM. The organic layer was collected and dryed over anhydrous Na_2SO_4. The solvent was evaporated, and the crude intermediate was obtained as yellow oil. This compound (2.0 g, 3.94 mmol) was dissolved in toluene, then 2 equiv. *p*-toluene sulfonic acid was added, and the mixture was refluxed overnight at 110°C under argon. After cooling to room temperature, the mixture was concentrated and the crude product was purified by column chromatography and pentane as eluent to obtain the product as a white solid (1.43 g, yield 74%). ^1H NMR (600 MHz, $CDCl_3$, 298 K): δ 7.23 (d, *J* = 8.6 Hz, 4H, Ha), 7.15 –7.11 (m, 6H, Hd, He), 7.00 (dd, *J* = 6.6, 3.0 Hz, 4H, Hc), 6.87 (d, *J* = 8.5 Hz, 4H, Hb). ^{13}C NMR (151 MHz, $CDCl_3$, 298 K): δ 143.02, 142.18, 138.36, 132.94, 131.14, 131.00, 127.92, 126.89, 120.71.

2.3 Synthesis of L:

2 (283 mg, 0.58 mmol, 1.0 equiv.), pinacol ester **1** (360 mg, 1.44 mmol, 2.5 equiv.), [Pd(PPh_3)$_4$] (66.7 mg, 0.058 mmol, 10 mol%) and K_3PO_4 (440 mg, 2.08 mmol, 3.6 equiv.) were mixed in mixture solvents of a 1,4-dioxane: H_2O = 4:1 (20 mL). The mixture was degassed (via freeze-thaw cycles) and then heated to 95 °C under N_2 atmosphere for 48 h. After cooling to room temperature, the solvents were removed under vacuum. Aqueous HCl (2 M, 50 mL) was added, and the mixture extracted with $CHCl_3$ (3 × 30 mL), organic phases combined and dried over Na_2SO_4. The solvent was removed under reduced pressure. The crude product was purified by column chromatog raphy ($CHCl_3$: Pentane = 2:1) to provide the product as a bright yellow solid (212 mg, yield 64%). ^1H NMR (600 MHz, [D6]DMSO, 298 K): δ 10.83 (s, 2H, Ha), 10.25 (s, 2H, Hb), 7.71 (d, *J* = 8.2 Hz, 2H, Hc), 7.52 (d, *J* = 8.5 Hz, 4H, Hf), 7.24 (dd, *J* = 8.1, 1.4 Hz, 2H, Hd), 7.20 –7.12 (m, 12H, He, Hi, Hj, Hg), 7.05 (dd, *J* = 8.2, 1.3 Hz, 4H, Hh). ^{13}C NMR (151 MHz, [D6]DMSO, 298 K): δ191.26, 161.00, 146.99, 143.58, 142.96, 141.75, 139.24, 136.60, 131.55, 130.68, 129.99, 127.99, 126.88, 126.37, 121.23, 117.89, 114.58.

2.4 Synthesis of *s*-R:

Ligand **L** (11.8 mg, 0.021 mmol, 1.0 equiv.) and (1*S*,2*S*)-(-)-1,2-diphenyl-1,2-ethanediamine (5.25 mg. 0.025 mmol, 1.2 equiv.) were dissolved in 2 mL DCM: MeOH=1:1. After addition of CH_3COOH (20 μL), the solution was stirred at room temperature for 10 h. Then, the solvent was removed under reduced pressure. The residue was washed with anhydrous methanol (3 × 1 mL) and dried in vacuum to provide the product as yellow powder in quantitative yield. ^1H NMR (600 MHz, $CDCl_3$, 298 K): δ13.31 (s, 4H, Hn), 8.34 (s, 4H, He), 7.32 (d, *J* = 8.4 Hz, 8H, Hi), 7.21 – 7.17 (m, 20H, Hb, Ha, Hc), 7.14 –7.09 (m, 20H, Hf, Hh, Hl, Hm), 7.07 –7.05 (m, 16H, Hj, Hk), 6.99 (dd, *J* = 8.0, 1.7 Hz, 4H, Hg), 4.68 (s, 4H, Hd). ^{13}C NMR (151 MHz, $CDCl_3$, 298 K): δ 166.14, 161.41, 145.08, 143.93, 141.92,

140.21, 139.66, 138.14, 132.35, 132.20, 131.67, 128.76, 128.25, 128.13, 127.97, 126.97, 126.69, 117.86, 117.79, 80.73, 30.06. HR ESI-MS: Calculated for $C_{108}H_{80}N_4O_4$, *m/z* 1498.6285 [M + H]$^+$, found: 1498.6285

***r*-R** was synthesised following the same procedure by using (1*R*,2*R*)-(-)-1,2-diphenyl-1,2-ethanediamine.

2.5 Synthesis of R':

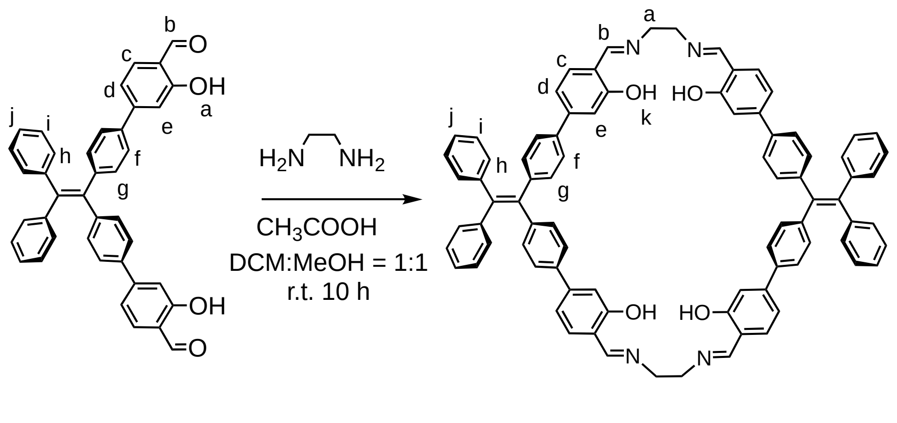

L **R'**

Figure S2. Synthesis of achiral macrocycle **R'**.

Ligand **L** (10 mg, 0.017 mmol, 1.0 equiv.) and ethanediamine (1.26 mg, 0.021 mmol, 1.2 equiv.) were dissolved in 2ml DCM: MeOH=1:1. Then the solution was stirred at room temperature for 10 h. The precipitate was collected by centrifugation and washed with anhydrous methanol (3 × 1 mL). The product was dried in vacuum to provide the product as yellow powder in quantitative yield. ^{1}H NMR (500 MHz, $CDCl_3$, 298 K): δ 13.15 (s, 4H, Hk), 8.29 (s, 4H, He), 7.33 (d, *J* = 8.3 Hz, 8H, Hf), 7.19 (d, *J* = 8.0 Hz, 4H, Hc), 7.13 – 7.09 (m, 16H, Hi, Hh), 7.09 – 7.06 (m, 8H, He, Hj), 7.06 – 7.04 (d, 8H, Hg), 7.02 (dd, *J* = 8.0, 1.7 Hz, 4H, Hd), 3.98 (s, 8H, Ha).

2.6 Synthesis of M_2R and Zn_2R':

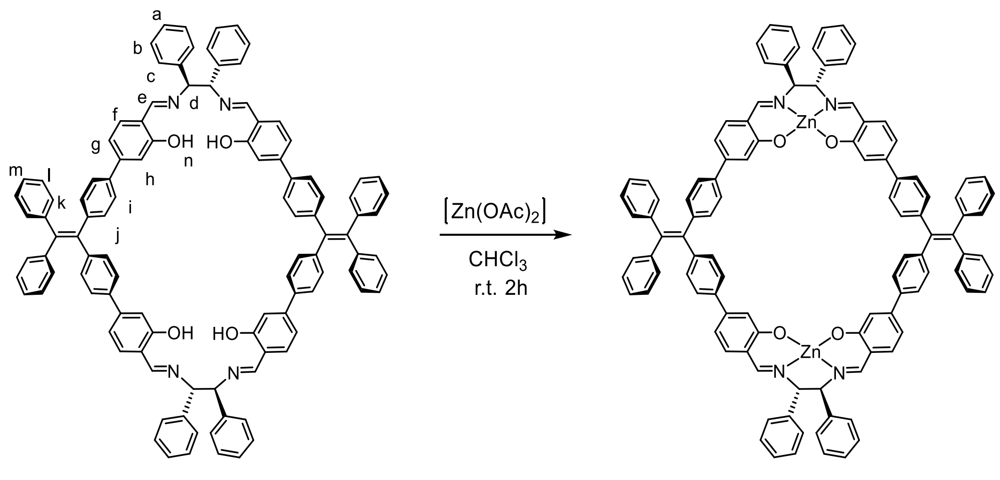

***s*-R** ***s*-Zn$_2$R**

Figure S3. Self-assembly of ***s*-Zn$_2$R**.

R (5 mg, 3.34 μmol, 1 equiv.) was dissolved in $CHCl_3$ (2 mL), stock solution of $[Zn(OAc)_2]$ (61 μL, 20 mg/mL, 2 equiv.) in DMSO was added and stir at room temperature for 2 h. After the reaction, solvents were removed, and the crude product was dissolved in a minimal amount of DCM and precipitated with Et_2O to give the product (4.9

mg, yield 90%) as yellow solid. ^{1}H NMR (600 MHz, [D6]DMSO, 298 K): δ 8.25 (s, 4H, He), 7.44 –7.41 (m, 8H, Hc), 7.40 (d, *J* = 8.3 Hz, 8H, Hi), 7.36 (t, *J* = 7.7 Hz, 8H, Hb), 7.29 –7.26 (m, 4H, Ha), 7.21 (dd, *J* = 8.0, 6.4 Hz, 8H, Hl), 7.19 –7.15 (m, 4H, Hm), 7.08 –7.04 (m, 20H, Hf, Hj, Hk), 6.81 (d, *J* = 1.7 Hz, 4H, Hh), 6.55 (dd, *J* = 8.0, 1.8 Hz, 4H, Hg), 5.11 (s, 4H, Hd). ^{13}C NMR (176 MHz, [D6]DMSO, 298 K): δ 171.24, 169.27, 144.80, 143.51, 142.81, 141.23, 140.73, 139.34, 138.77, 135.56, 131.74, 130.84, 128.53, 128.05, 127.74, 127.51, 126.83, 126.26, 120.83, 118.50, 111.38, 72.40.

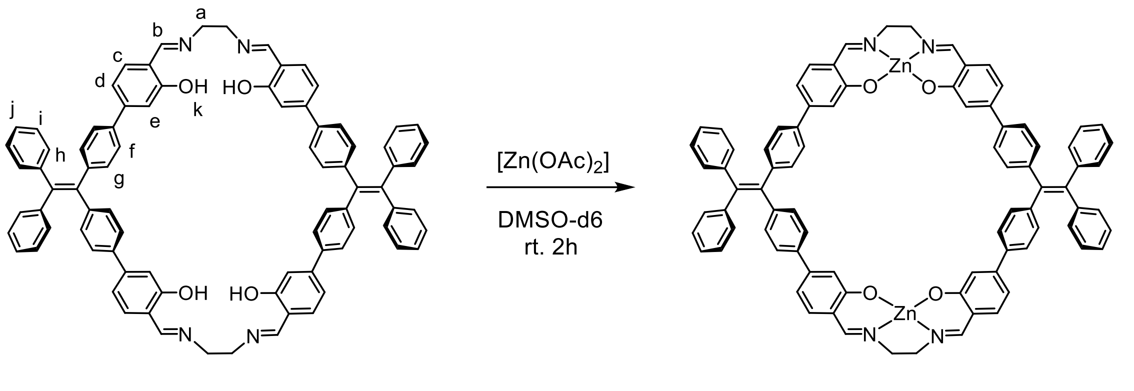

R' **Zn2R'**

Figure S4. Self-assembly of **Zn$_2$R'**.

R' (5 mg, 4.19 μmol, 1 equiv.) was dissolved in DMSO (0.5 mL), a stock solution of [Zn(OAc)$_2$] (77 μL, 20 mg/mL, 2 equiv.) in DMSO was added and stir at room temperature for 2 h. After the reaction, solvents were removed, and the crude product was dissolved in a minimal amount of DCM and precipitated with Et_2O to give the product as yellow solid. ^{1}H NMR (500 MHz, [D6]DMSO, 298 K): δ 8.47 (s, 4H, Hb), 7.41 (d, *J* = 8.2 Hz, 8H, Hf), 7.26-7.14 (m, 16H, Hc, H,j, Hi), 7.11 – 6.98 (m, 16H, Hg, Hh), 6.79 (d, *J* = 1.8 Hz, 4H, He), 6.61 (dd, *J* = 8.0, 1.8 Hz, 4H, Hd), 3.76 (s, 8H, Ha). ^{13}C NMR (151 MHz, [D6]DMSO, 298 K): δ 170.94, 167.58, 144.04, 143.58, 142.71, 140.64, 139.42, 138.78, 135.22, 131.75, 130.88, 128.05, 126.19, 120.71, 118.56, 111.10, 55.59.

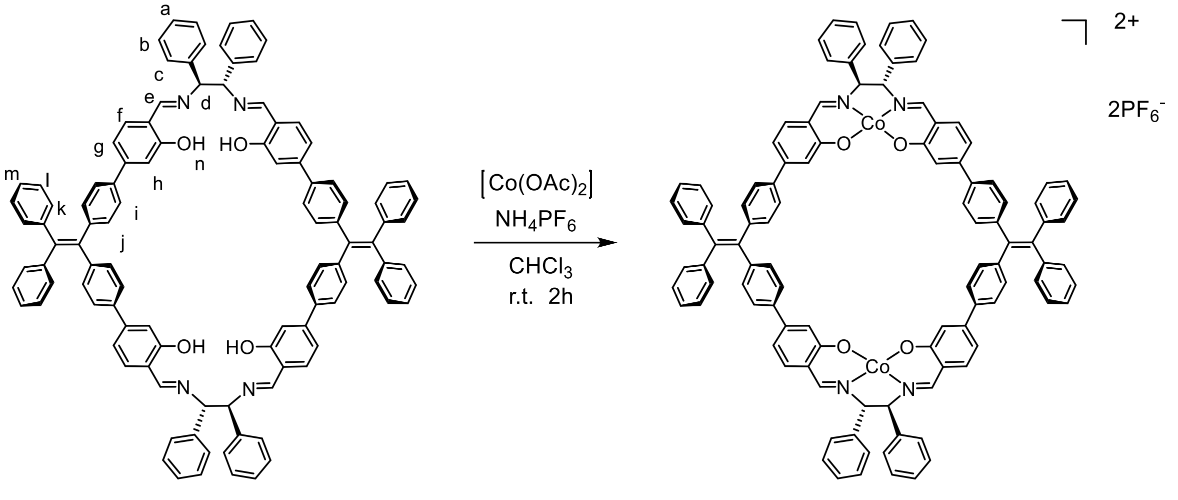

***s*-R** ***s*-Co2R**

Figure S5. Self-assembly of ***s*-Co$_2$R**.

R (5 mg, 3.34 μmol, 1 equiv.) was dissolved in $CHCl_3$ (2 mL), a stock solution of [Co(OAc)$_2$] (59 μL, 20 mg/mL, 2 equiv.) in DMSO was added and stirred at room temperature for 2 h. After stirring for 15 min under aerobic conditions, the NH_4PF_6 was added and stirred for 6 h at room temperature. After the reaction, solvents were

removed, and the crude product was dissolved in a minimal amount of DCM and precipitated with Et_2O to give the product as dark red solid. For analytical data see below and discussion in the main text.

3. NMR spectroscopic results

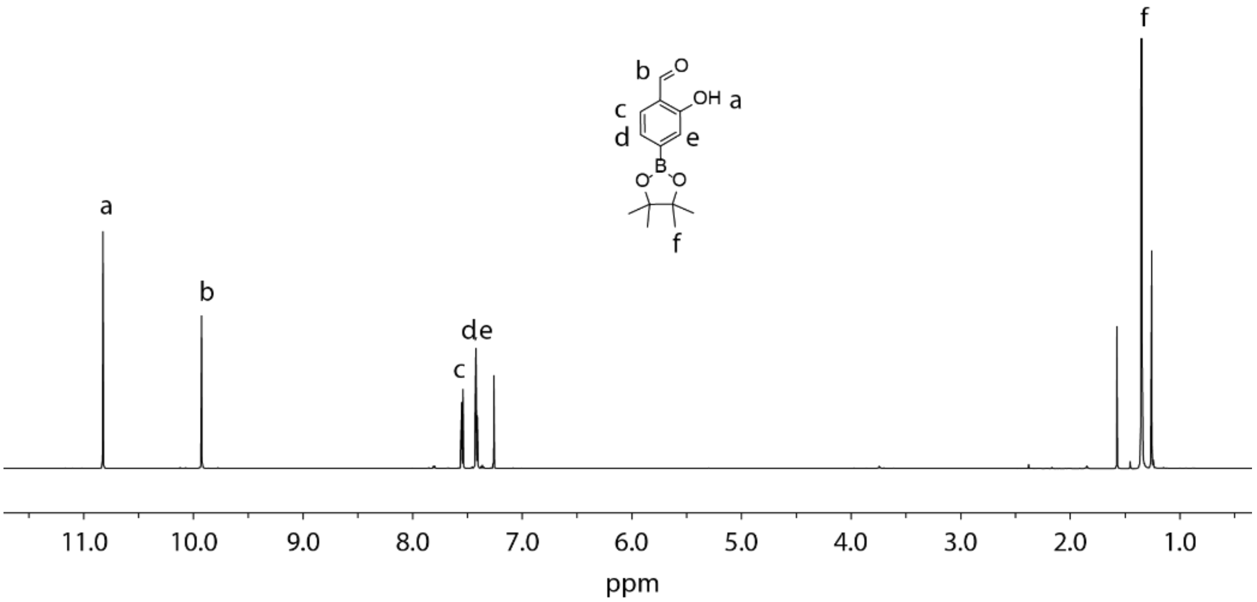

Figure S6. ^{1}H NMR spectrum of compound **1** (600 MHz, 298 K, $CDCl_3$).

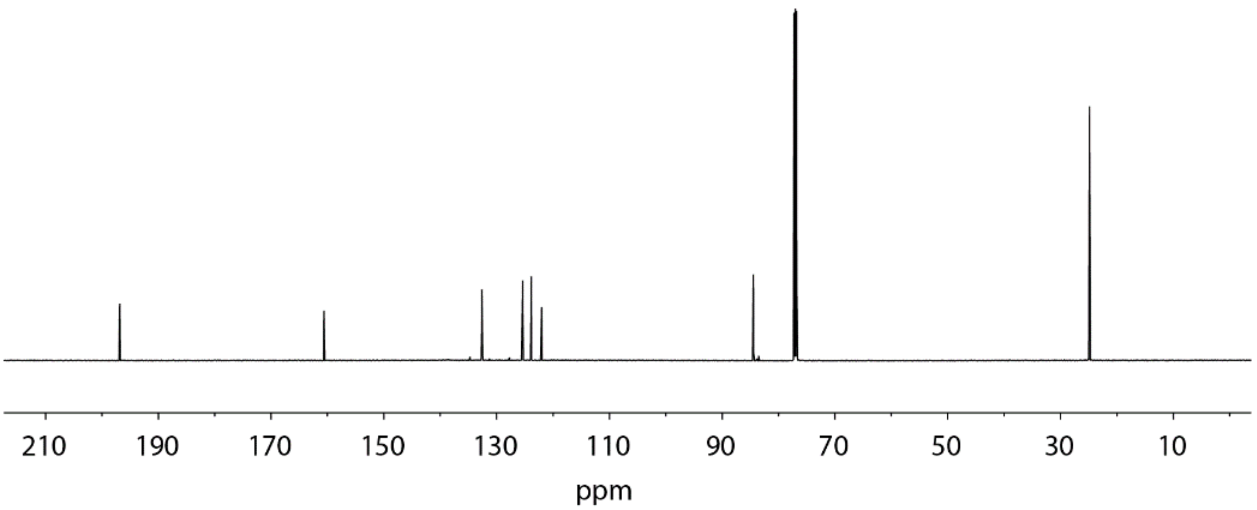

Figure S7. ^{13}C NMR spectrum of compound **1** (151 MHz, 298 K, $CDCl_3$).

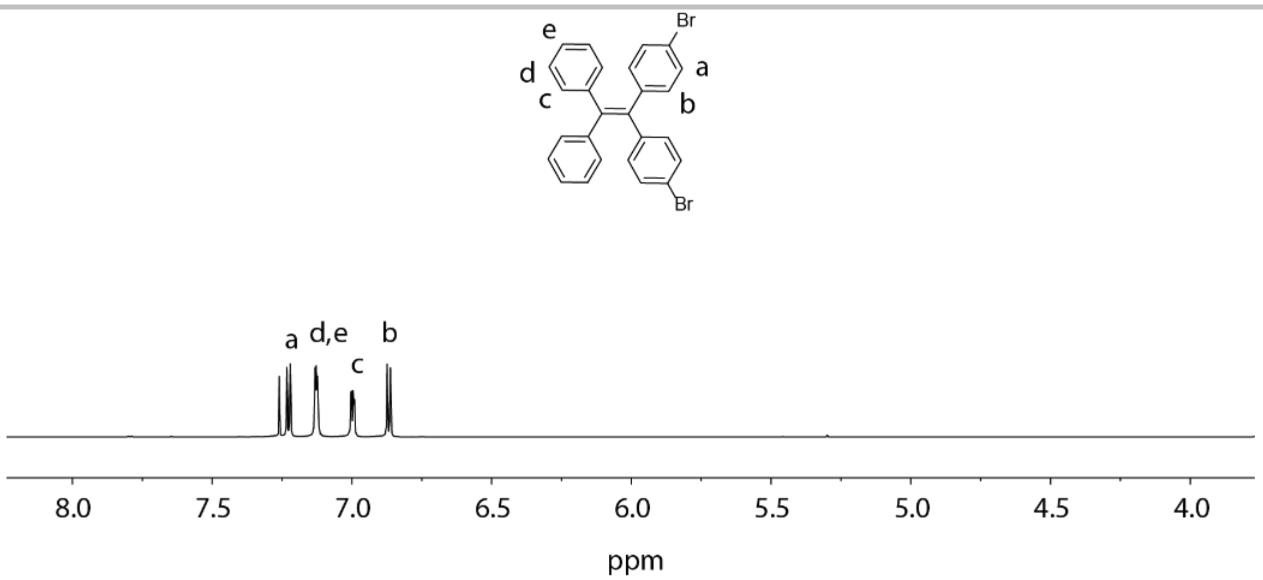

Figure S8. ^{1}H NMR spectrum of compound **2** (600 MHz, 298 K, $CDCl_3$).

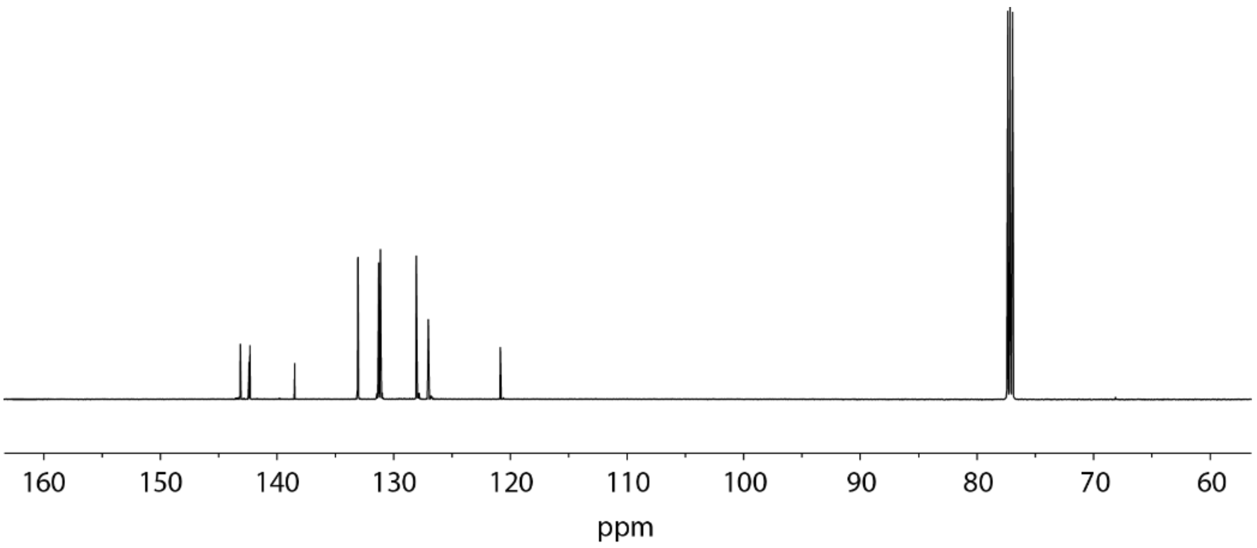

Figure S9. ^{13}C NMR spectrum of compound **2** (151 MHz, 298 K, $CDCl_3$).

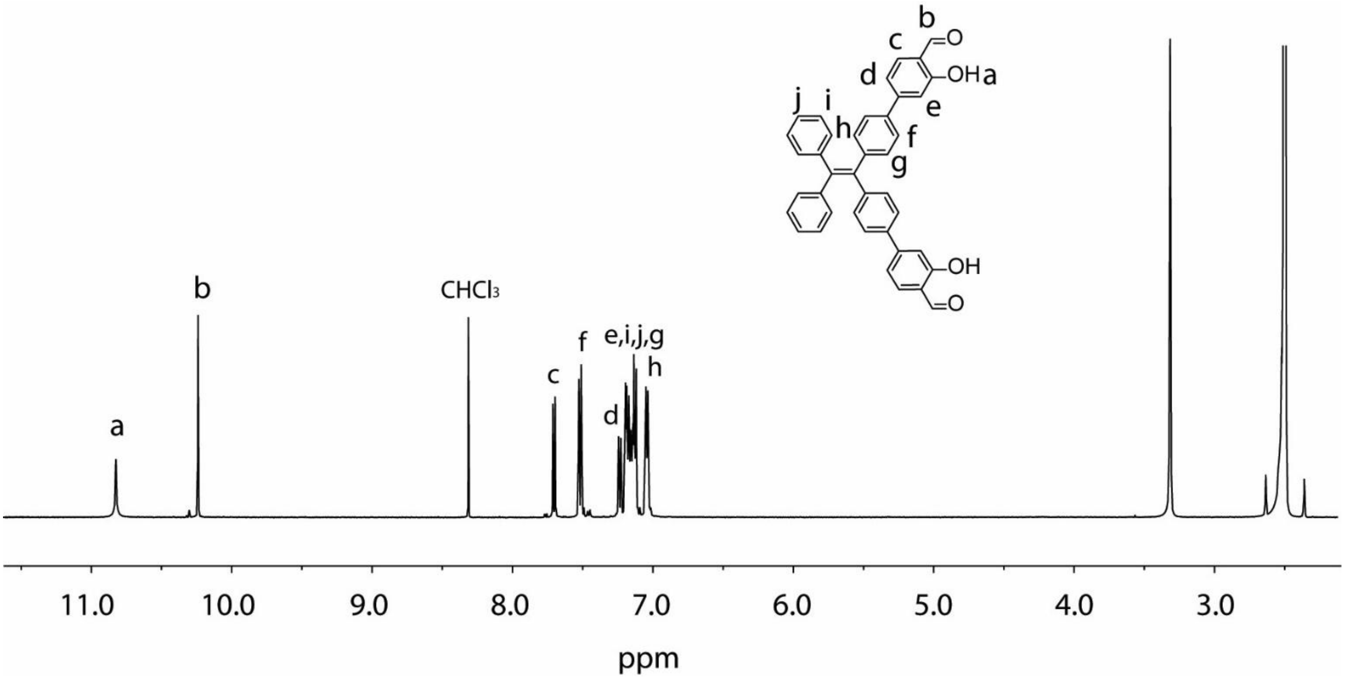

Figure S10. ^{1}H NMR spectrum of **L** (600 MHz, 298 K, [D6]DMSO).

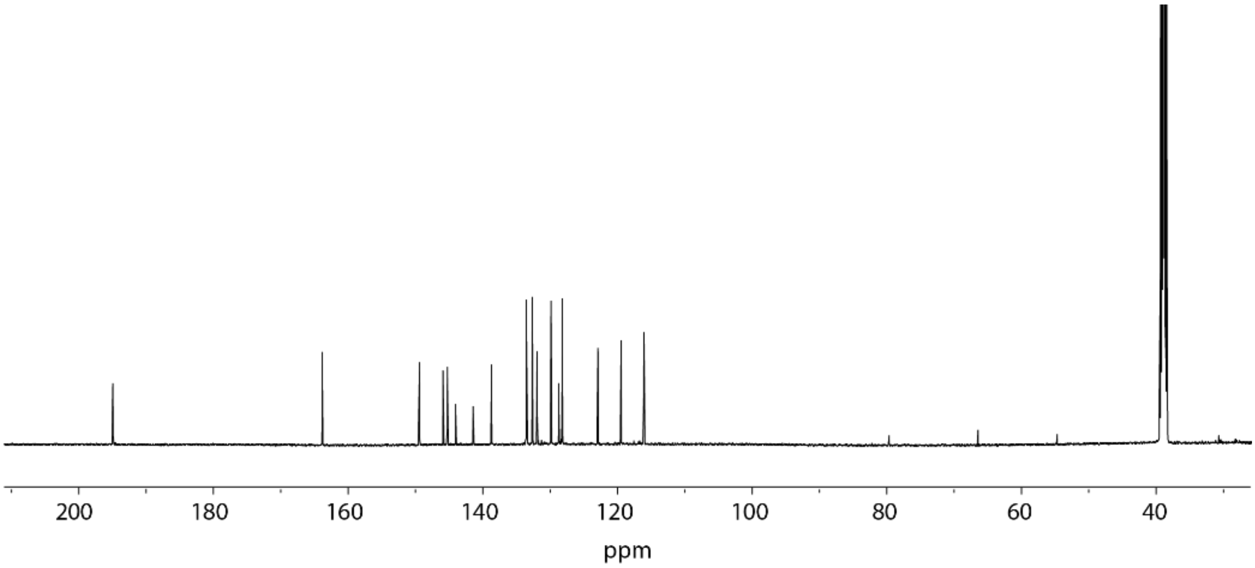

Figure S11. ^{13}C NMR spectrum of **L** (151 MHz, 298 K, [D6]DMSO).

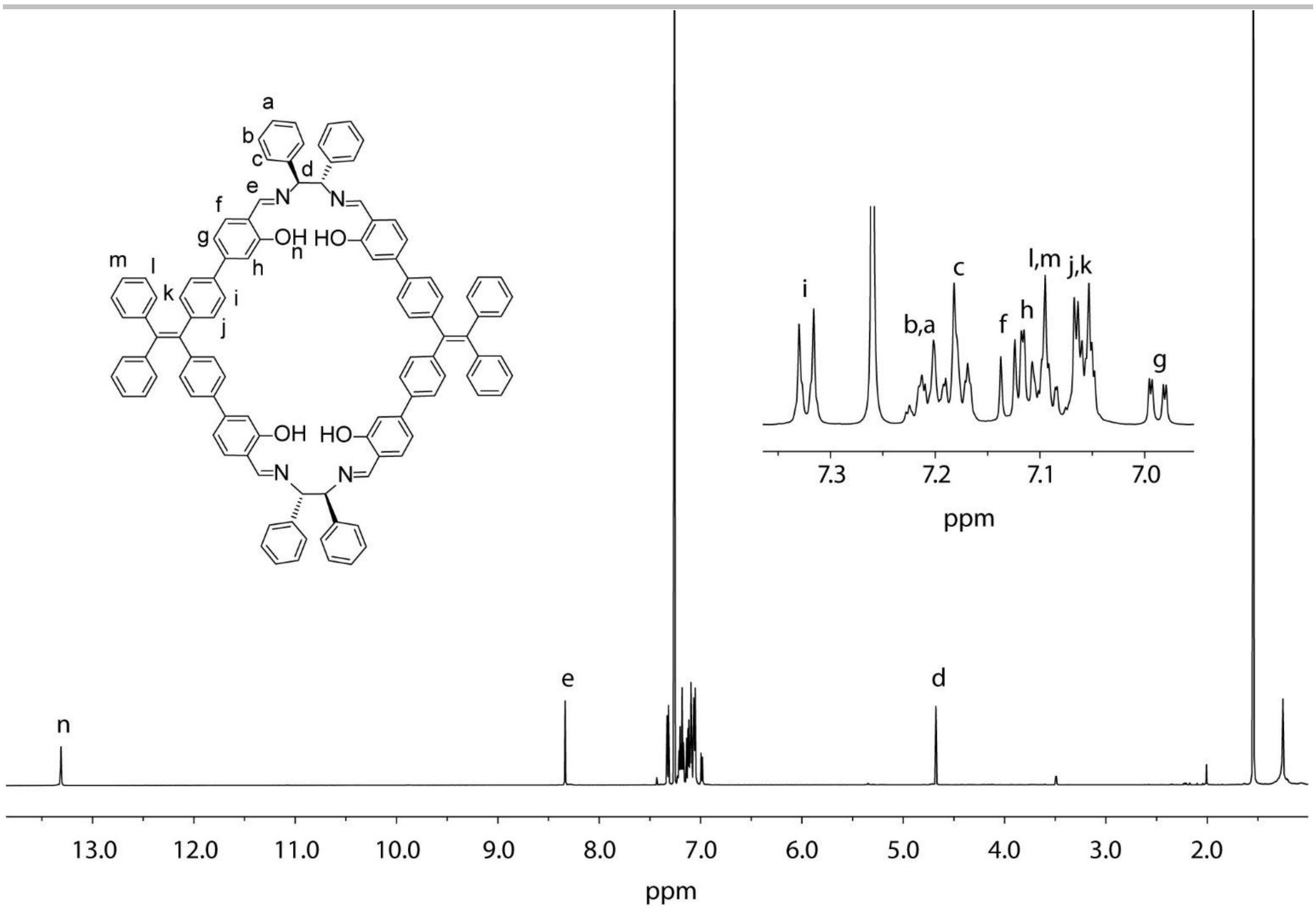

Figure S12. ^{1}H NMR spectrum of **R** (600 MHz, 298 K, $CDCl_3$).

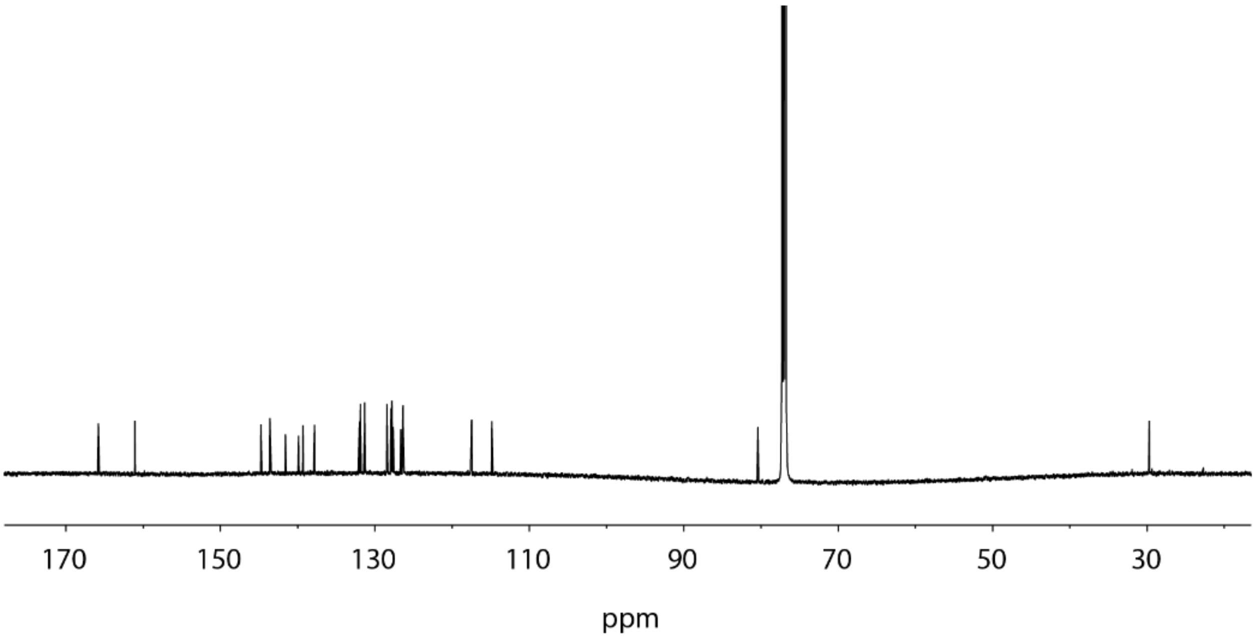

Figure S13. ^{13}C NMR spectrum of **R** (151 MHz, 298K, $CDCl_3$).

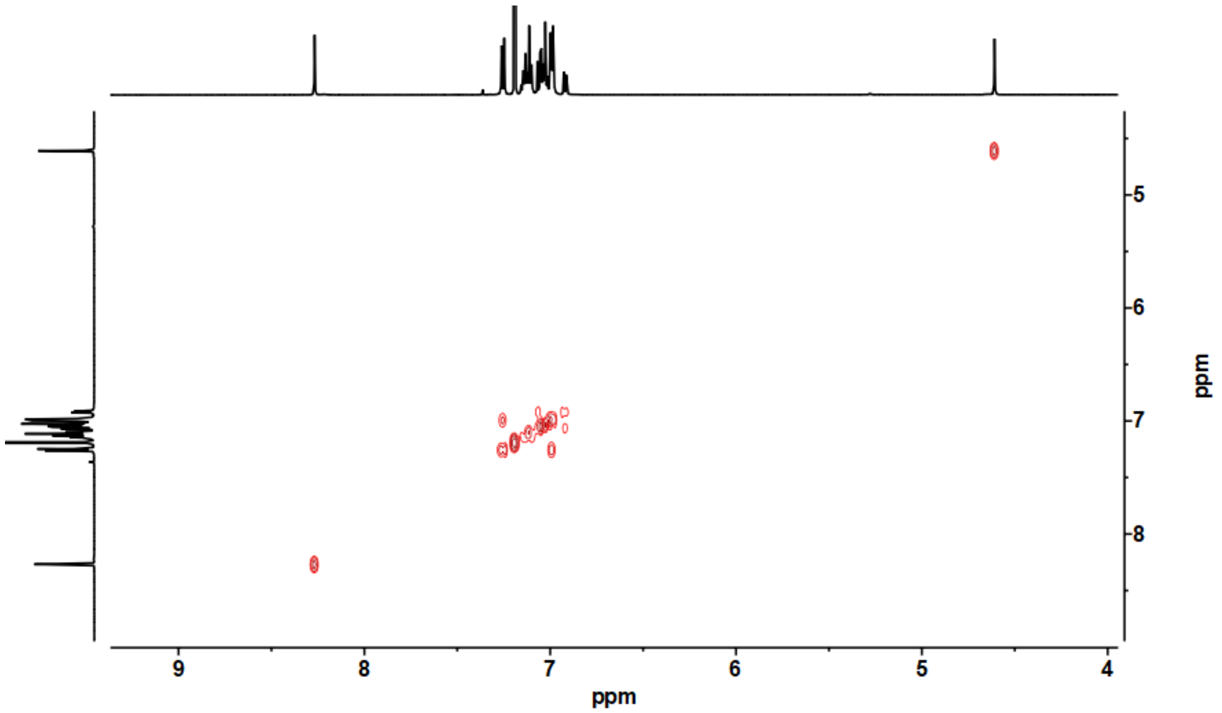

Figure S14. ^{1}H-^{1}H COSY spectrum of **R** (600 MHz, 298 K, $CDCl_3$).

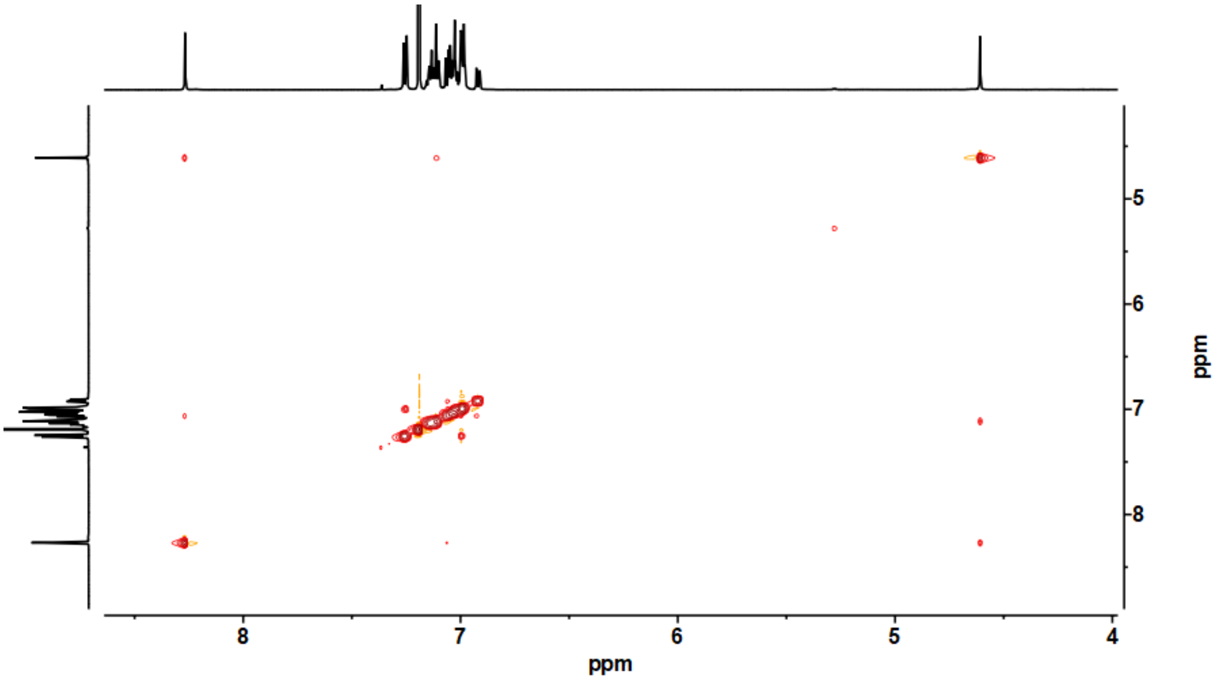

Figure S15. ^{1}H-^{1}H NOESY spectrum of **R** (600 MHz, 298 K, $CDCl_3$).

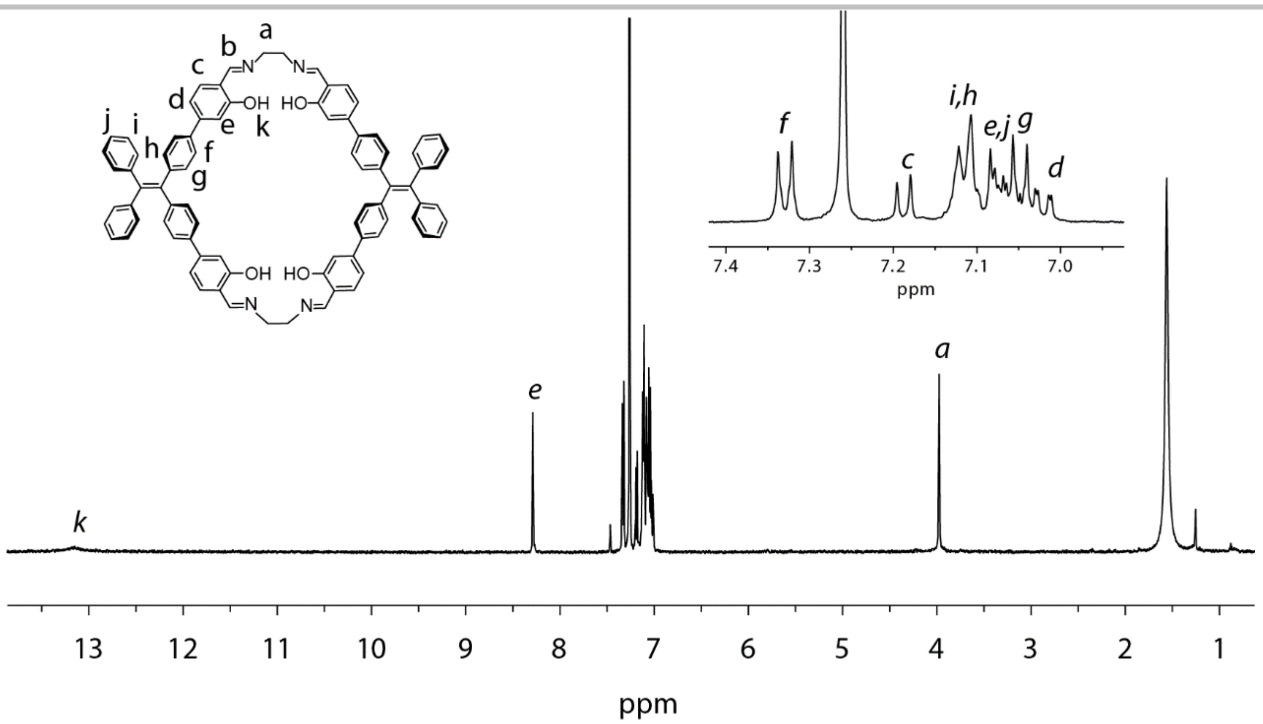

Figure S16. ^{1}H NMR spectrum of **R'** (500 MHz, 298 K, $CDCl_3$).

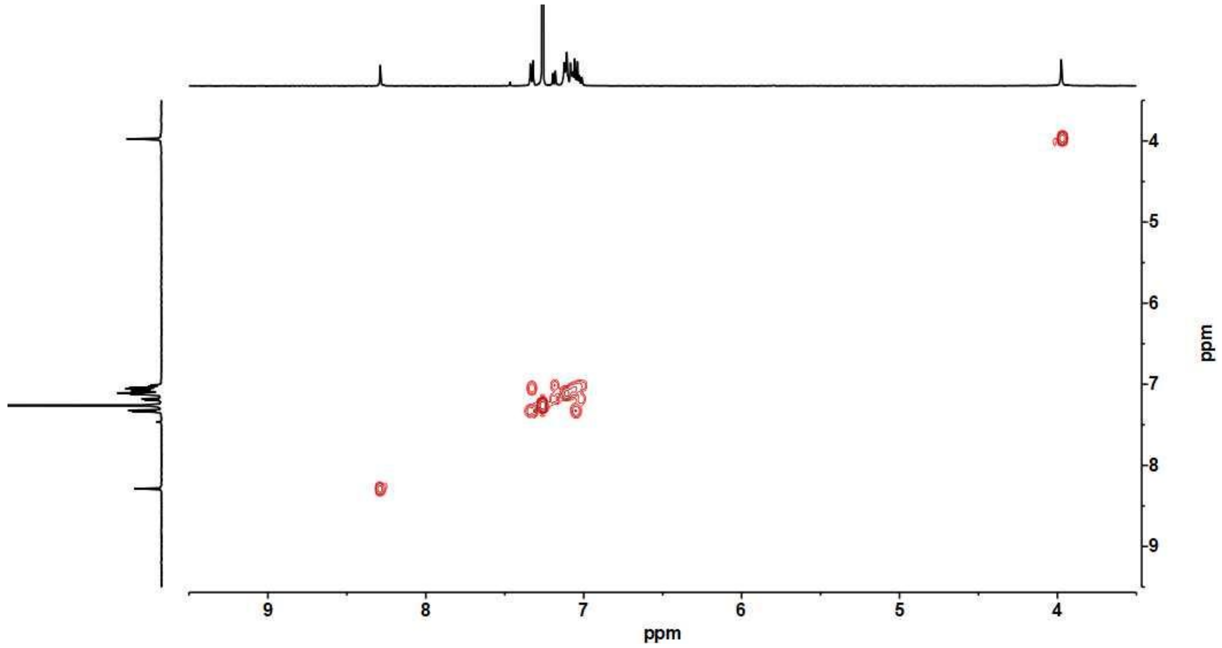

Figure S17. ^{1}H-^{1}H COSY spectrum of **R'** (500 MHz, 298 K, $CDCl_3$).

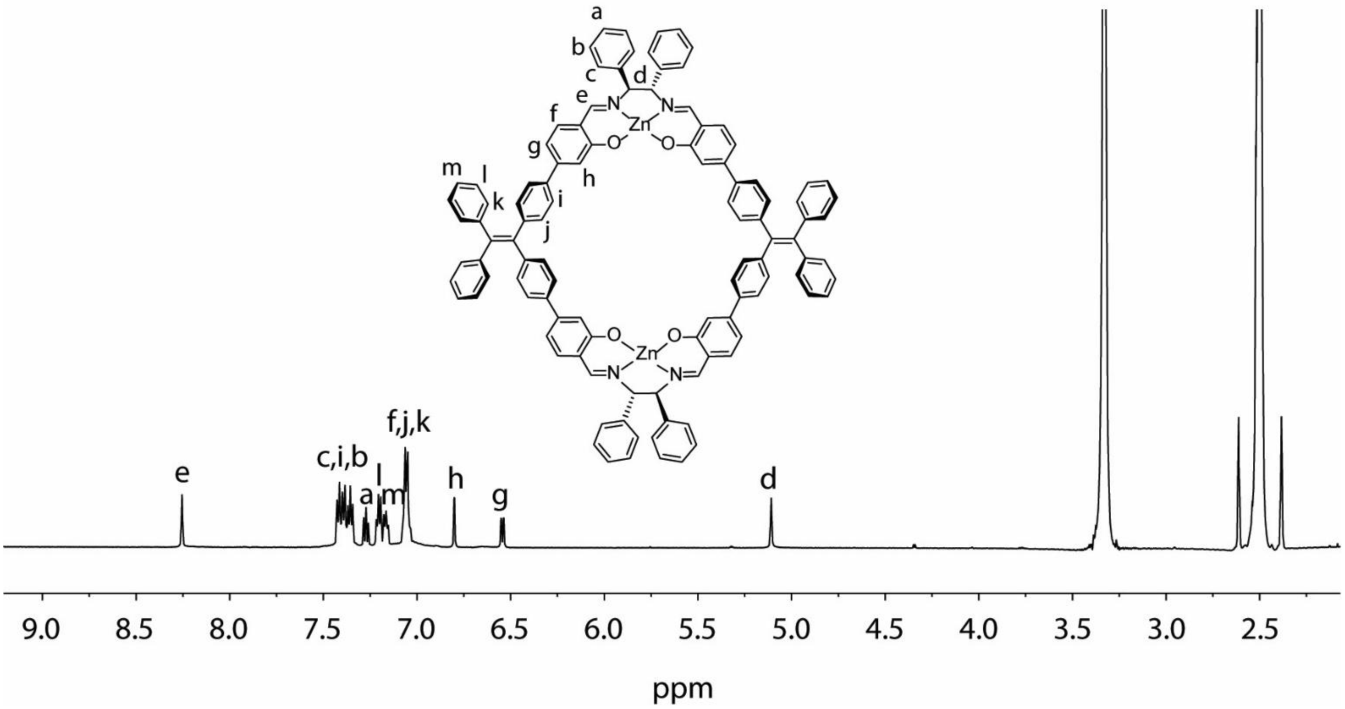

Figure **S18**. ^{1}H NMR spectrum of **Zn$_2$R** in [D6]DMSO (600 MHz, 298 K).

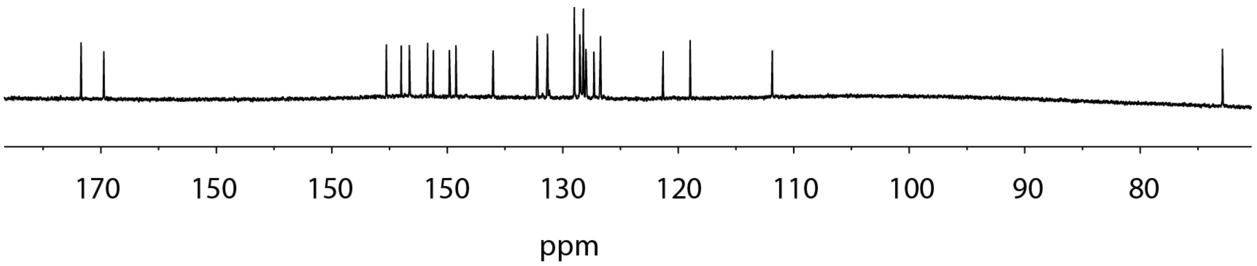

Figure S19. ^{13}C NMR spectrum of **Zn$_2$R** (151 MHz, 298 K, [D6]DMSO).

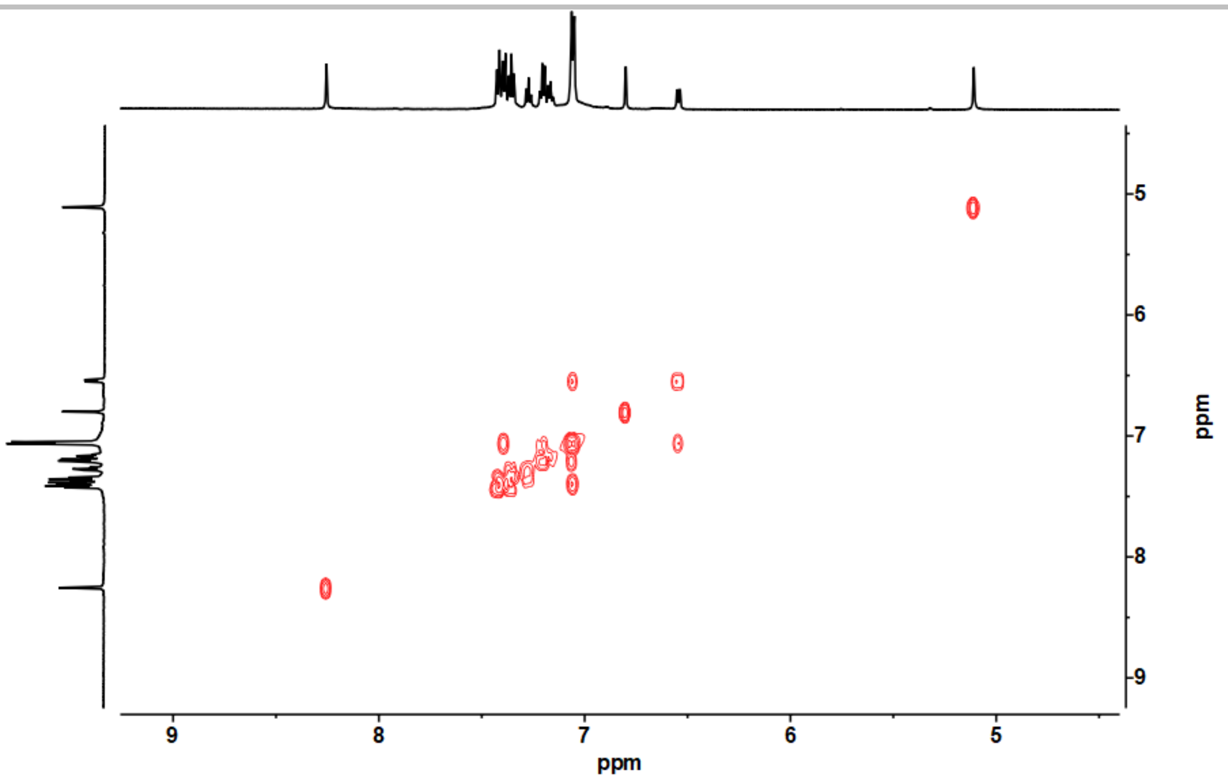

Figure 20. ^{1}H-^{1}H COSY spectrum of **Zn_2R** (500 MHz, 298 K, [D6]DMSO).

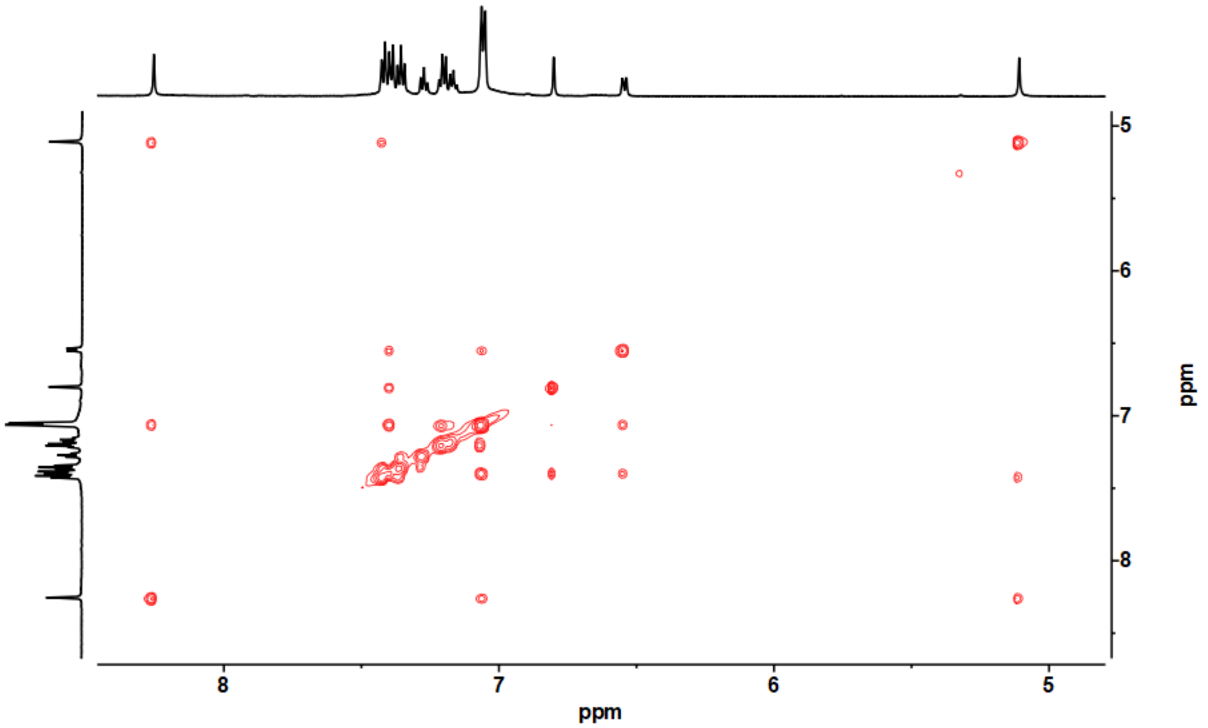

Figure S21. ^{1}H-^{1}H NOESY spectrum of **Zn_2R** (500 MHz, 298 K, [D6]DMSO).

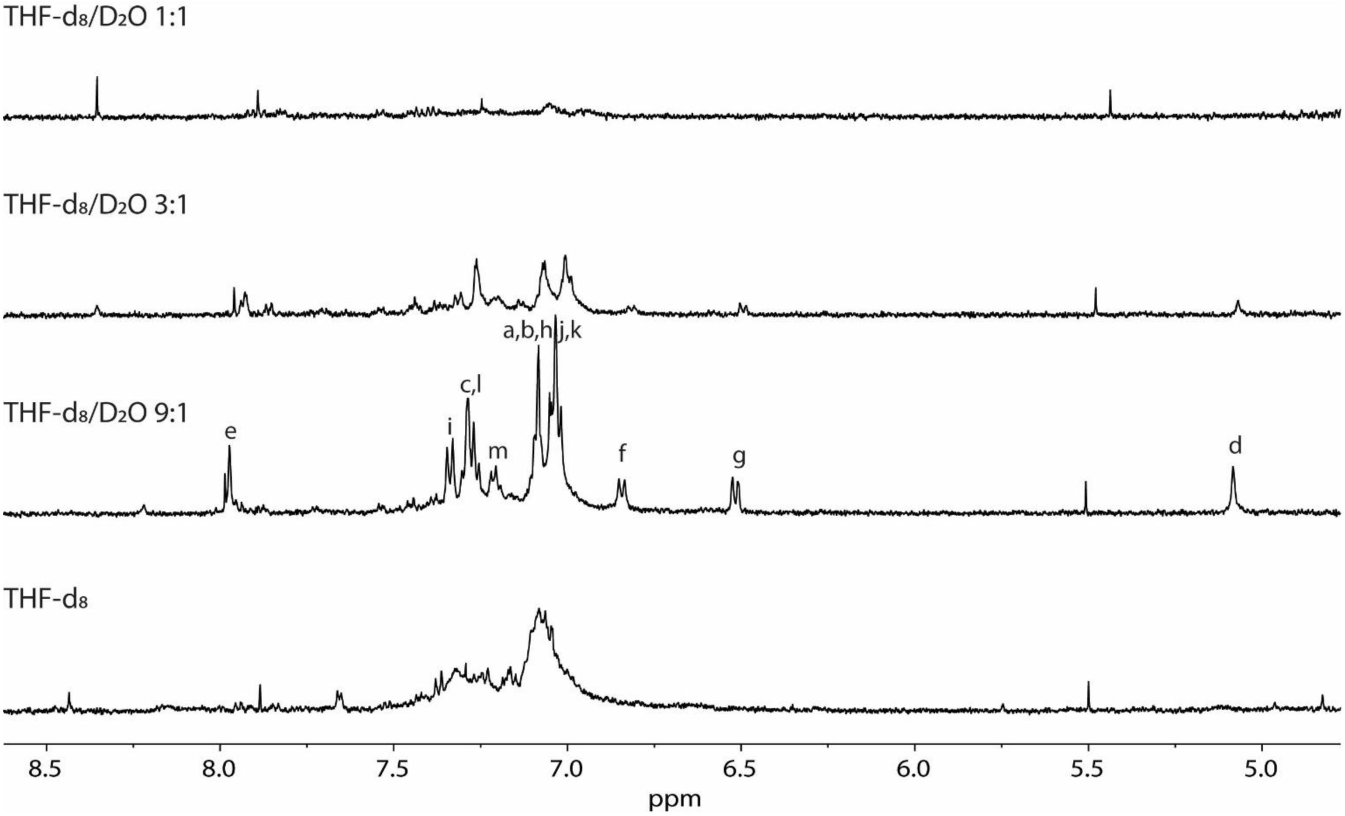

Figure **S22**. ^{1}H NMR spectrum of **Zn_2R** in [D8]THF and [D8]THF/D2O mixtures (500 MHz, 298 K).

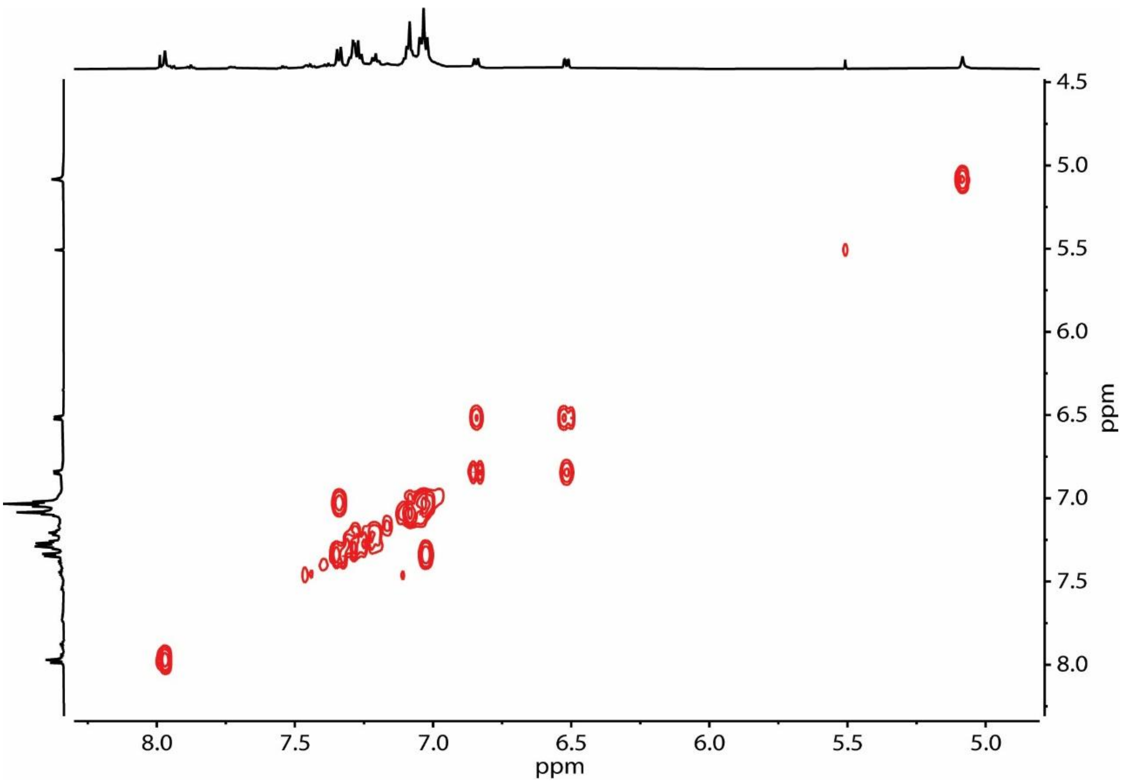

Figure 23. ^{1}H-^{1}H COSY spectrum of **Zn_2R** (600 MHz, 298 K, [D8]THF/D2O-9:1).

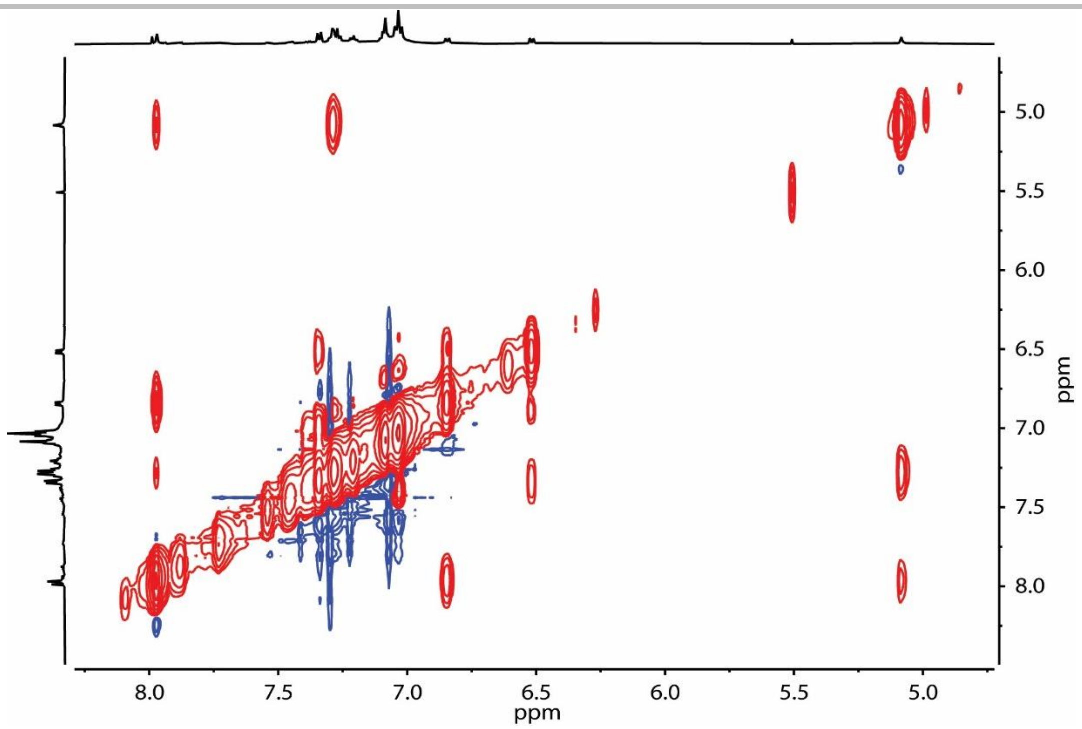

Figure S24. ^1H-^1H NOESY spectrum of **Zn_2R** (600 MHz, 298 K, [D8]THF/D2O-9:1).

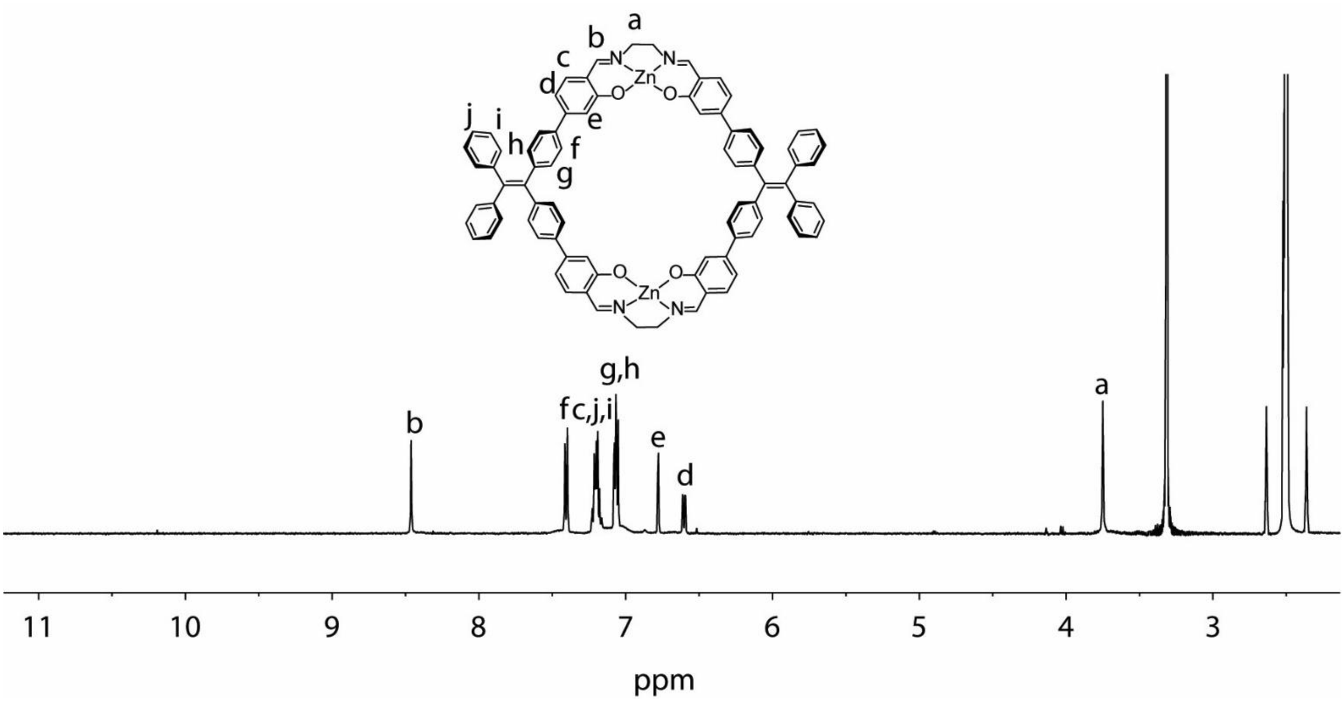

Figure S25. ^1H NMR spectrum of **Zn_2R'** (600 MHz, 298 K, $CDCl_3$).

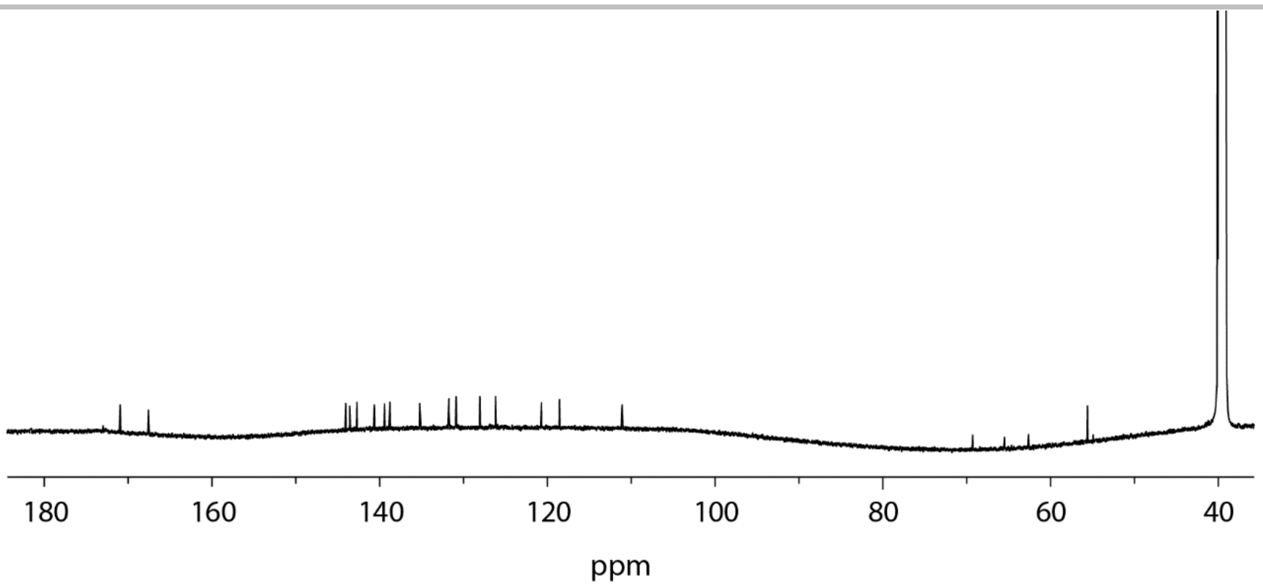

Figure S26. ^{13}C NMR spectrum of Zn_2**R'** (600 MHz, 298 K, $CDCl_3$).

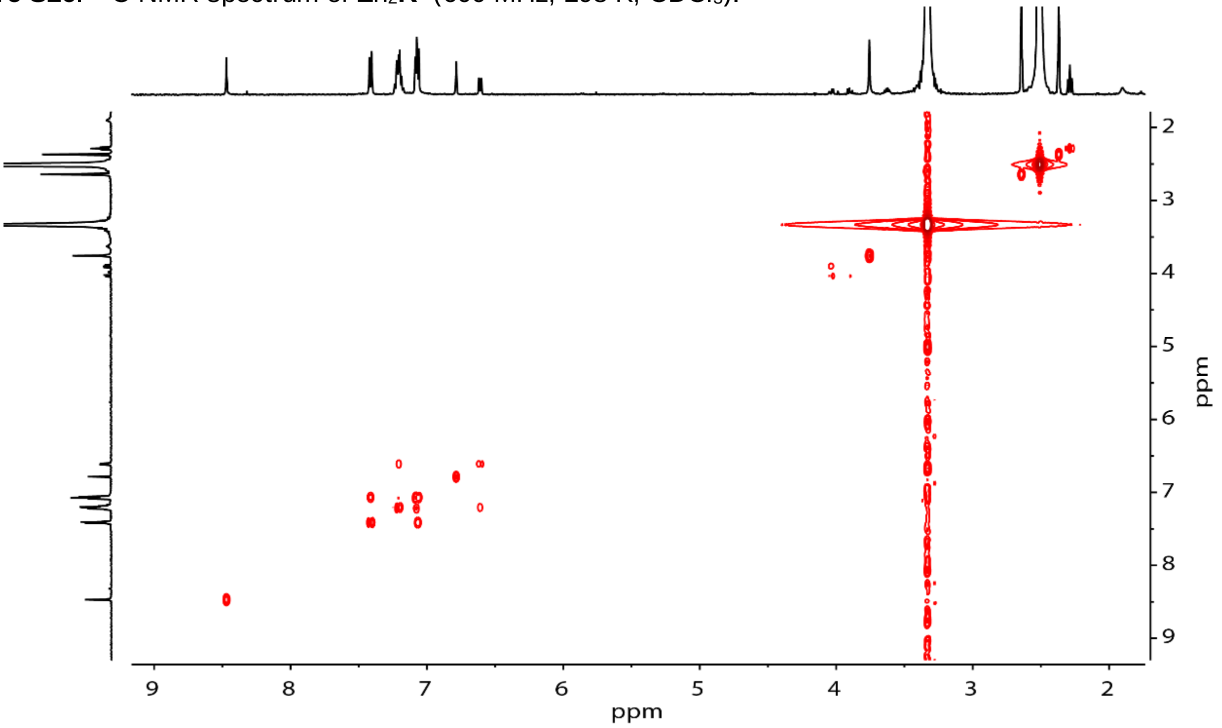

Figure S27. ^1H-^1H COSY spectrum of **ZnR'** (600 MHz, 298 K, [D6]DMSO).

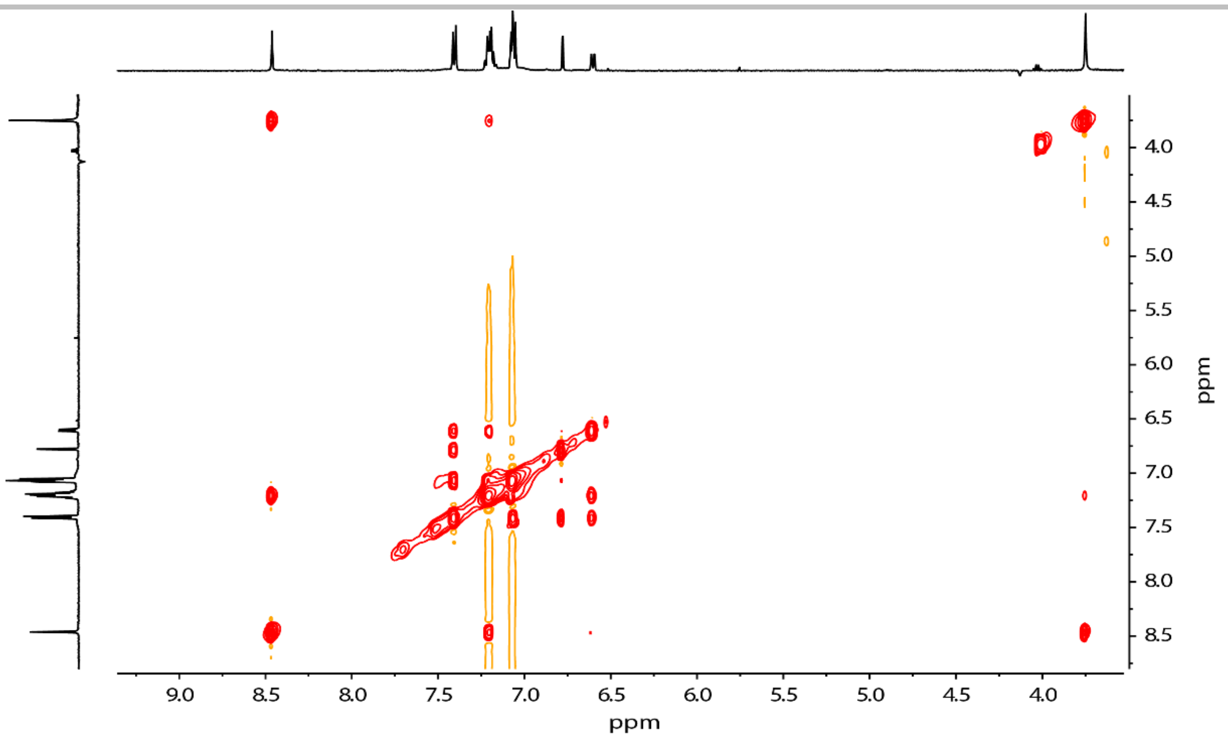

Figure S28. ^{1}H-^{1}H NOESY spectrum of **ZnR'** (600 MHz, 298 K, [D6]DMSO).

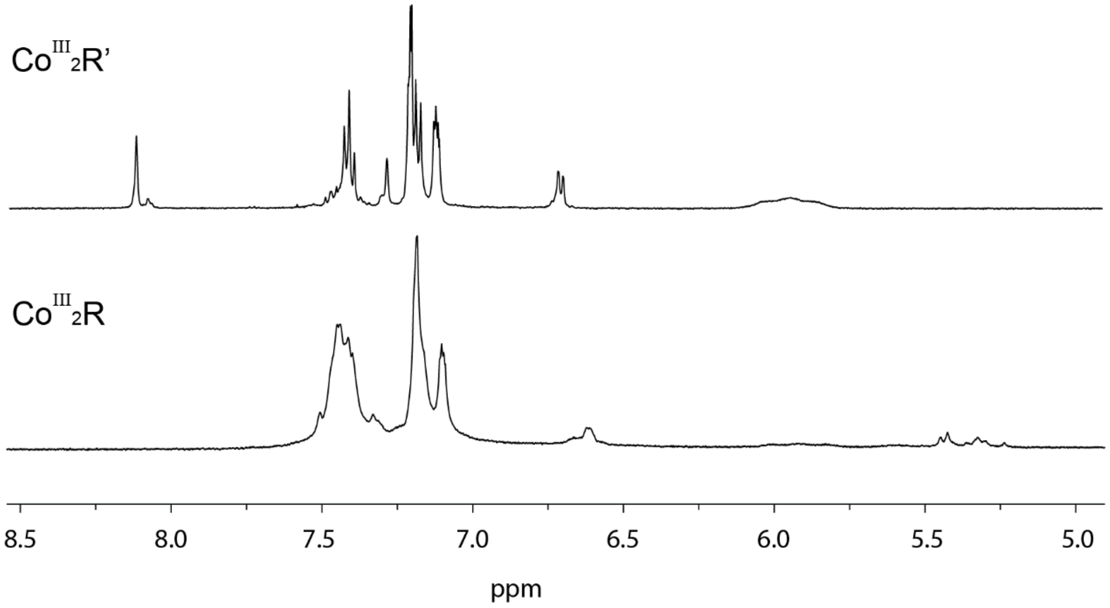

Figure S29. ^{1}H NMR spectra of **$Co^{III}{}_2R$** and **$Co^{III}{}_2R'$** (600 MHz, 298 K, CD_3CN).

3.1 ^{1}H DOSY NMR investigation

The hydrodynamic radii of the examined compounds were calculated according to the Stokes-Einstein equation:

$$D = \frac{k_B T}{6\pi\eta r_H}$$

Where D is the measured diffusion coefficient, k_B is the Boltzmann constant, T is the absolute temperature, η is the viscosity of the solvent and r_H is the hydrodynamic radius.

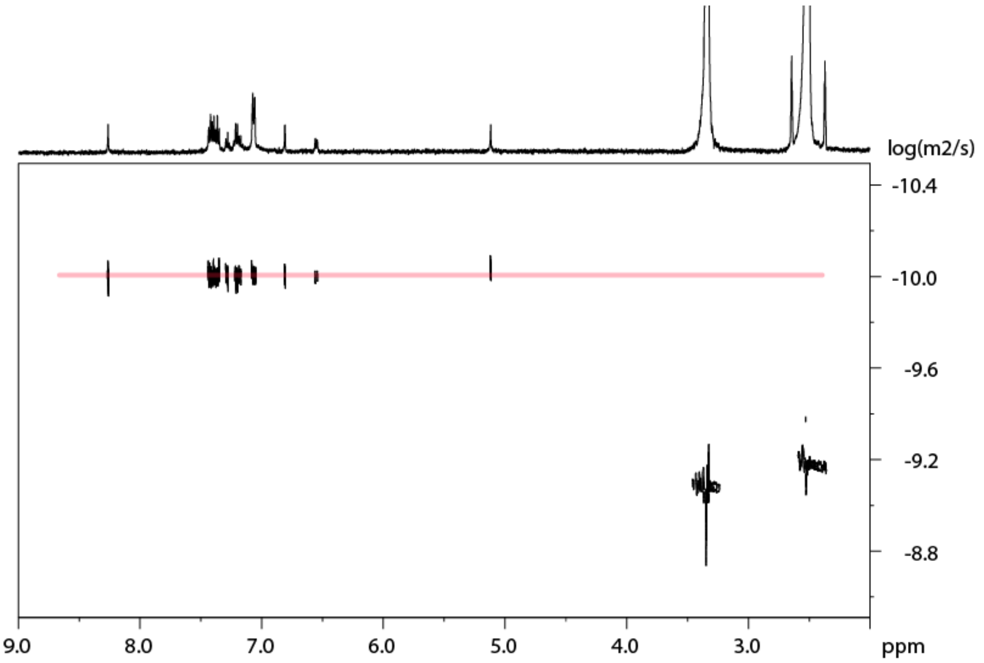

Figure S30. ^{1}H DOSY spectrum of **Zn$_2$R** (500 MHz, 298 K, [D6]DMSO). Diffusion coefficient: D = 9.54 × 10^{-11} m^2 s^{-1}, r_H = 11.47 Å.

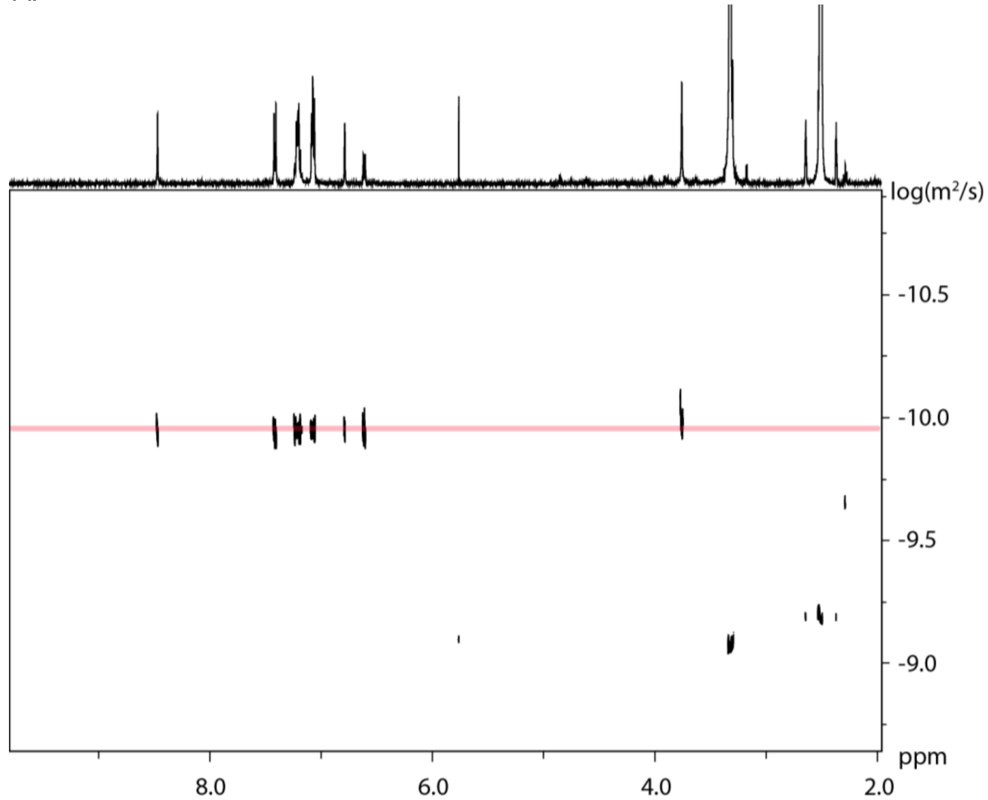

Figure S31. ^{1}H DOSY spectrum of **ZnR'** (500 MHz, 298 K, [D6]DMSO). Diffusion coefficient: D = 1.11 × 10^{-10} m^2 s^{-1}, r_H = 9.84 Å

4. Further ESI mass spectrometric results

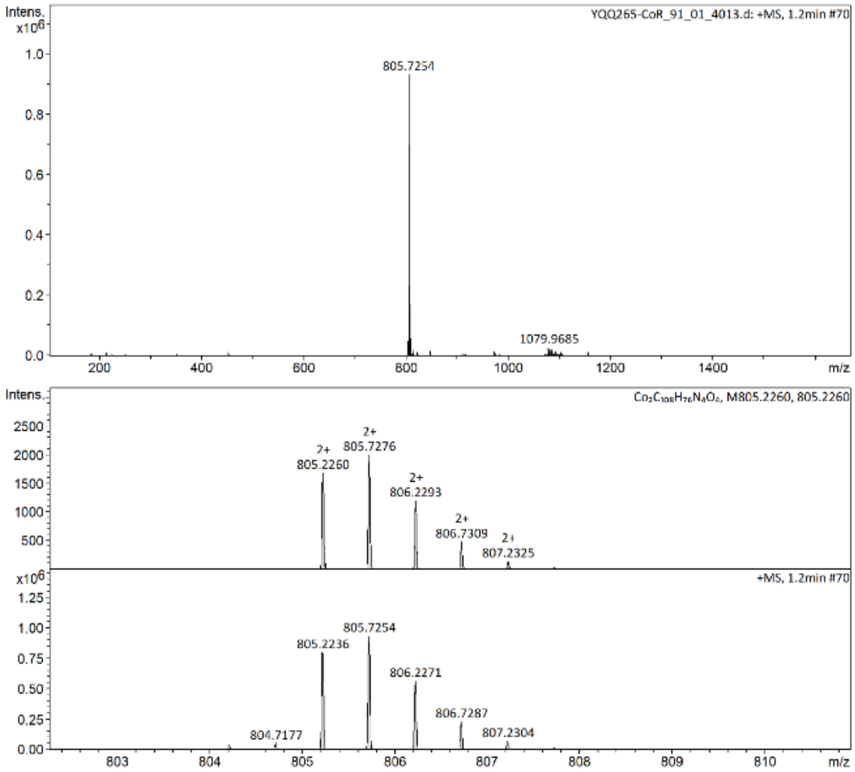

Figure S32. ESI-MS spectrum of **Co^{III}$_2$R** clearly showing the assembled ring containing two Co(III) cations as the most prominent signal.

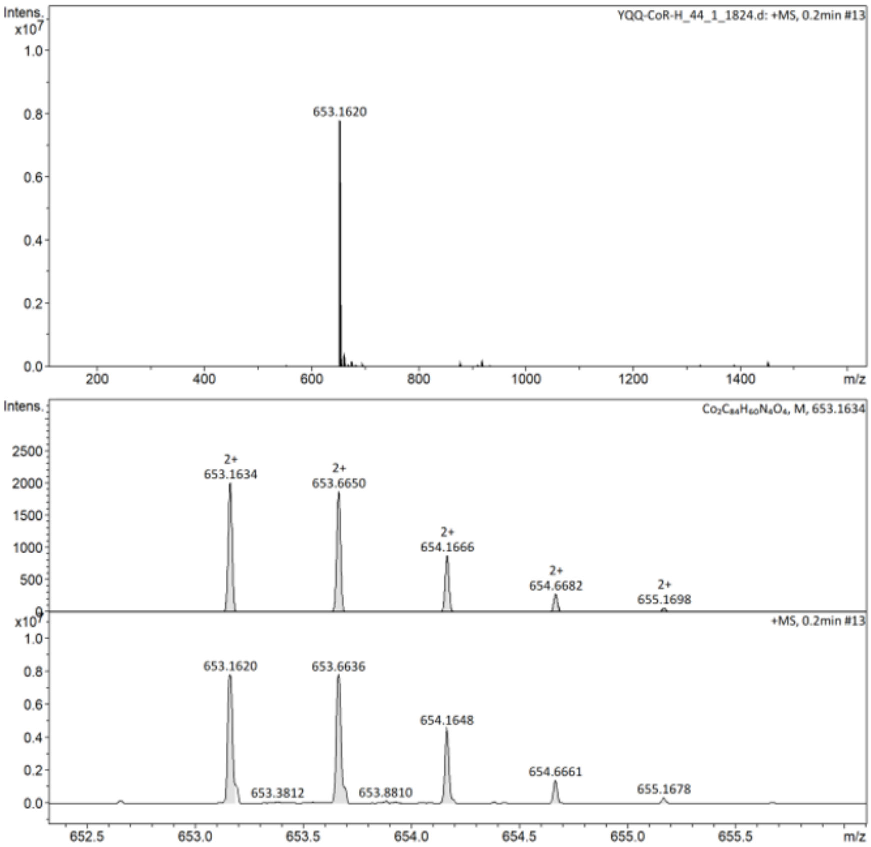

Figure S33. ESI-MS spectra of **Co^{III}$_2$R‘** clearly showing the assembled ring containing two Co(III) cations as the most prominent signal.

5. X-ray crystal structure analysis

Three different supramolecular ring structures were studied using single-crystal X-ray crystallography. The crystals of the supramolecular assemblies were extremely sensitive to loss of organic solvent. Due to very thin plate- or needle-shaped crystals, the analysis was further hampered by the limited scattering power of the samples, not allowing to reach the desired atomic resolution using an in-house microfocussed X-ray CuK_{α} source for one sample. In the other two cases, gaining detailed structural insight required cryogenic crystal handling and highly brilliant synchrotron radiation. Hence, diffraction data of most supramolecular assemblies was collected during two beamtime shifts at macromolecular synchrotron beamline P11, PETRA III, at DESY, Hamburg. Counterion and solvent molecule disorder required carefully adapted macromolecular refinement protocols employing geometrical restraint dictionaries, similarity restraints and restraints for anisotropic displacement parameters (ADPs). Further details can be found in the Crystallographic tables and discussion below.

Table S1: Crystal data and refinement details for supramolecular ring structures

**Compound**	**R'**	***r*-Zn$_2$R**	***s*-Co$_2$R**
**CIF ID**	**QY30**	**qy3ep_sq**	**qy2e_sq**
**CCDC no**	**2244465**	**2244466**	**2244467**
Empirical formula	$C_{90}H_{70}Cl_{18}N_4O_4$	$C_{222}H_{166}N_{10}O_{10}Zn_4$	$C_{57}H_{47}CoN_3O_4$
Formula weight	1909.60	3395.12	896.90
Temperature [K]	126(2)	100(2)	100(2)
Crystal system	monoclinic	monoclinic	monoclinic
Space group (number)	$P2_1/c$ (14)	$C2$ (5)	$P2_1$ (4)
a [Å]	12.3216(12)	11.2740(18)	16.553(3)
b [Å]	27.162(3)	34.462(4)	18.006(4)
c [Å]	13.7645(13)	13.8390(15)	24.146(5)
α [Å]	90	90	90
β [Å]	106.898(4)	99.85(2)	96.02(3)
γ [Å]	90	90	90
Volume [Å^3]	4407.7(7)	5297.6(12)	7157(3)
Z	2	1	4
ρ_{calc} [g/cm^3]	1.439	1.064	0.832
μ [mm^{-1}]	5.552	0.462	0.250
F(000)	1952	1768	1876
Crystal size [mm^3]	0.050×0.050×0.010	0.100×0.100×0.005	0.200×0.020×0.010
Crystal color	colourless	colourless	red
Crystal shape	plate	plate	plate
Radiation	CuK_α (λ=1.54178 Å)	synchrotron (λ=0.6888 Å)	synchrotron (λ=0.6888 Å)
2Θ range [°]	6.51 to 108.46 (0.95 Å)	2.29 to 52.36 (0.78 Å)	1.64 to 48.41 (0.84 Å)
Index ranges	-12 ≤ h ≤ 12<br>-27 ≤ k ≤ 28<br>-14 ≤ l ≤ 14	-14 ≤ h ≤ 14<br>-44 ≤ k ≤ 44<br>-17 ≤ l ≤ 17	-19 ≤ h ≤ 19<br>-19 ≤ k ≤ 18<br>-28 ≤ l ≤ 28
Reflections collected	60824	33380	82719
Independent reflections	5368<br>R_{int} = 0.1251<br>R_{sigma} = 0.0606	10280<br>R_{int} = 0.0452<br>R_{sigma} = 0.0425	23594<br>R_{int} = 0.0281<br>R_{sigma} = 0.0247
Completeness to θ = 25.242°	99.8 %	97.2 %	94.3 %
Data / Restraints / Parameters	5368/844/553	10280/1152/580	23594/2546/1172
Goodness-of-fit on F^2	1.171	1.016	1.000
Final *R* indexes [I≥2σ(I)]	R_1 = 0.1964<br>wR_2 = 0.3901	R_1 = 0.0737<br>wR_2 = 0.2209	R_1 = 0.0612<br>wR_2 = 0.1828
Final *R* indexes [all data]	R_1 = 0.2183<br>wR_2 = 0.3999	R_1 = 0.1040<br>wR_2 = 0.2533	R_1 = 0.0846<br>wR_2 = 0.2101
Largest peak/hole [eÅ^3]	1.18/-0.63	1.26/-0.21	0.53/-0.16
Flack x parameter	-	Parsons (SHELX) = 0.152(14)<br>Hooft (PLATON) = 0.142(10)	Parsons (SHELX) = 0.104(7)<br>Hooft (PLATON) = 0.050(5)

Crystal structure determination of R'

Colorless, plate-shaped crystals of R' were grown by slow evaporation of a chloroform solution at room temperature. A single crystal in mother liquor was pipetted onto a glass slide containing NVH oil. To avoid collapse of the crystal lattice, the crystal was quickly mounted onto a 0.2 mm nylon loop and immediately flash-cooled in liquid nitrogen. Diffraction data were collected at 126(2) K on a Bruker D8 Venture with INCOATEC microfocus sealed tube, Iys 3.0 using multilayer optics as monochromator and a Photon II detector. The diffractometer was equipped with an Oxford Cryostream 800 low temperature device and used CuK_α radiation (λ = 1.54178 Å). In total 29 sweeps with a detector distance of 38 mm and exposure times from 5s/° for low order (2Θ = 0°) up to 35s/° for high order (2Θ = 57°) were collected with an increment of 1° per image. All data were integrated with SAINT and a multi-scan absorption correction using SADABS was applied. The data was cut at 0.95 Å, with mean I/Sig(I) > 2 in the outer resolution shell. The structure was solved by intrinsic phasing/direct methods using SHELXT[3] and refined with SHELXL[4] using 22 CPU cores for full-matrix least-squares routines on F^2 and ShelXle[5] as a graphical user interface.

Specific refinement details of R'

The asymmetric unit contains one half of the ring and three co-crystallized chloroform solvent molecules. Disorder of one of the chloroform molecules was modeled with two discrete positions using a free variable for occupancy refinement. Hydrogen atoms of Hydroxy groups were modeled using restraints assuming an intermolecular hydrogen bond to the imine nitrogen close by.

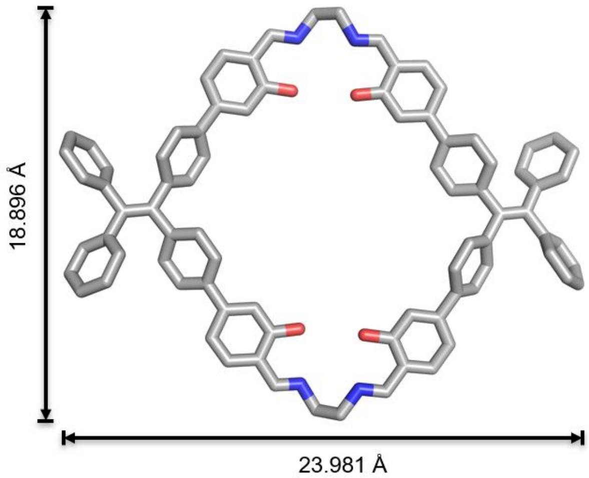

Figure S34. Single crystal X-ray structures of **R'** (solvent molecules and hydrogens are not shown for clarity).

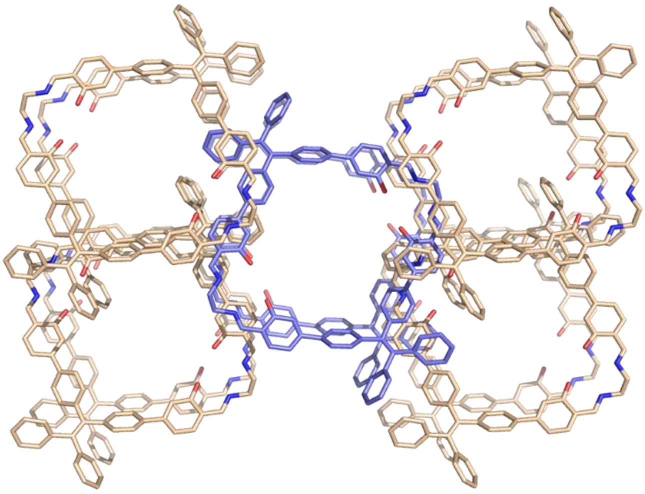

Figure S35. The packing structures of **R'** in top view (solvent molecules and hydrogens are not shown for clarity)

Crystal structure determination of *r*-Zn_2R

Colourless plate-shaped crystals of ***r*-Zn_2R** were grown by slow vapor diffusion of dietylether (Et_2O) into the solution of of *r*-Zn_2R in Dimethyllformamide (DMF) at room temperature. Single crystals in mother liquor were pipetted onto a glass slide containing NVH oil. To avoid collapse of the crystal lattice, the crystal was quickly mounted onto a 0.1 mm nylon loop and immediately flash-cooled in liquid nitrogen. Crystals were stored at cryogenic temperature in dry shippers, in which they were safely transported to macromolecular beamline P11 at Petra III ,[6] DESY, Hamburg, Germany. A wavelength of λ = 0.6888 Å was chosen using a liquid N_2 cooled double crystal monochromator. Single crystal X-ray diffraction data was collected at 100(2) K on a single axis goniometer, equipped with an Oxford Cryostream 800 and an Eiger 2x 16M detector. 3600 diffraction images were collected in a 360° φ sweep at a detector distance of 154 mm, 100% filter transmission, 0.1° step width and 0.1 seconds exposure time per image, reaching a resolution of 0.78 Å in the detector edges. Data integration and reduction were undertaken using XDS.[7] The structure was solved by intrinsic phasing/direct methods using SHELXT [3] and refined with SHELXL[4] using 22 CPU cores for full-matrix least-squares routines on F^2 and ShelXle[5] as a graphical user interface and the DSR program plugin was employed for modeling.[8,9]

Specific refinement details for *r*-Zn_2R

Stereochemical restraints for the organic **ring R** (residue AQA) dimethylsulfoxide solvent molecule (residue DMF) were generated by the GRADE program using the GRADE Web Server (http://grade.globalphasing.org) and applied in the refinement. A GRADE dictionary for SHELXL contains target values and standard deviations for 1,2-distances (DFIX) and 1,3-distances (DANG), as well as restraints for planar groups (FLAT). All displacements for non-hydrogen atoms were refined anisotropically. The refinement of ADP's for carbon, nitrogen and oxygen atoms was enabled by a combination of similarity restraints (SIMU) and rigid bond restraints (RIGU).[10] The contribution of the electron density from disordered counterions and solvent molecules, which could not be modeled with discrete atomic positions were handled using the SQUEEZE[11] routine in PLATON.[12] The solvent mask file (.fab) computed by PLATON was included in the SHELXL refinement via the ABIN instruction leaving the measured intensities untouched.

The asymmetric unit contains the ***r*-Zn_2R** ring structure and one dimethylsulfoxide solvent molecule coordinated to on Zn center, but disordered over a special position (twofold axis). The dimethylsulfoxide solvent molecule was modelled with 50% occupancy and negative Part number (PART -1). The enantiopurity distinguishing parameters according to Parsons = 0.152(14) (determined with the SHELX software) and Hooft = 0.142(10) (determined with the PLATON software) were found to be only moderate.

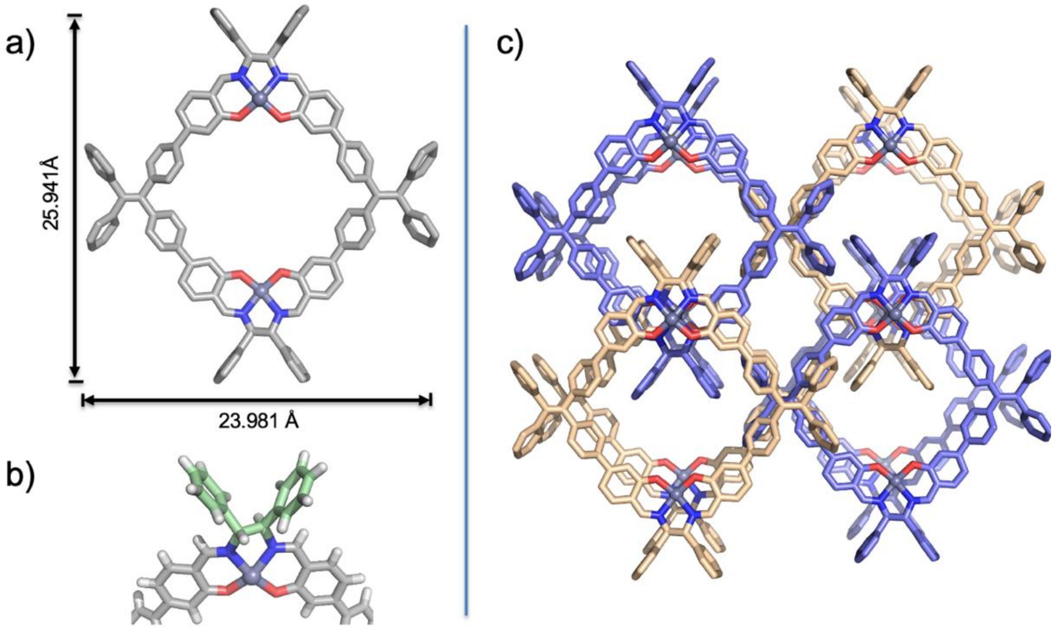

Figure S36. a) X-ray crystal structure of a) ring ***r*-Zn_2R** (C: gray, N: blue; O: red; Zn: dark purple), b) partial structure of the chiral salen moietiy and c) packing of ***r*-Zn_2R** rings in interdigitating columns viewed from the top (solvent molecules and hydrogens are not shown for clarity).

Crystal structure determination of *s*-Co_2R

Colourless plate-shaped crystals of ***s*-Co_2R** were grown by slow vapor diffusion of dietylether (Et_2O) into the solution of of *s*-Co_2R in Dimethyllformamide (DMF) at room temperature. Single crystals in mother liquor were pipetted onto a glass slide containing NVH oil. To avoid collapse of the crystal lattice, the crystal was quickly mounted onto a 0.1 mm nylon loop and immediately flash-cooled in liquid nitrogen. Crystals were stored at cryogenic temperature in dry shippers, in which they were safely transported to macromolecular beamline P11 at Petra III,[6] DESY, Hamburg, Germany. A wavelength of λ = 0.6888 Å was chosen using a liquid N_2 cooled double crystal monochromator. Single crystal X-ray diffraction data was collected at 100(2) K on a single axis goniometer, equipped with an Oxford Cryostream 800 and an Eiger 2x 16M detector. 1800 diffraction images were collected in a 360° φ sweep at a detector distance of 154 mm, 100% filter transmission, 0.1° step width and 0.1 seconds exposure time per image. Data integration and reduction were undertaken using XDS.[7] The data was cut at 0.84 Å, with mean I/Sig(I) > 1. The structure was solved by intrinsic phasing/direct methods using SHELXT[3] and refined with SHELXL[4] using 22 CPU cores for full-matrix least-squares routines on F^2 and ShelXle[5] as a graphical user interface and the DSR program plugin was employed for modeling.[8,9]

Specific refinement details for *s*-Co_2R

Stereochemical restraints for the organic **ring R** (residue AQA) dimethylsulfoxide solvent molecule (residue DMF) as well hydrogen from water molecule (residue H2O) were generated by the GRADE program using the GRADE Web Server (http://grade.globalphasing.org) and applied in the refinement. A GRADE dictionary for SHELXL contains target values and standard deviations for 1,2-distances (DFIX) and 1,3-distances (DANG), as well as restraints for planar groups (FLAT). All displacements for non-hydrogen atoms were refined anisotropically. The refinement of ADP's for carbon, nitrogen and oxygen atoms was enabled by a combination of similarity restraints (SIMU) and rigid bond restraints (RIGU).[10] The contribution of the electron density from disordered counterions and solvent molecules, which could not be modeled with discrete atomic positions were handled using the SQUEEZE[11] routine in PLATON.[12] The solvent mask file (.fab) computed by PLATON was included in the SHELXL refinement via the ABIN instruction leaving the measured intensities untouched.

The asymmetric unit contains the ***s*-Co_2R** ring structure, two dimethylsulfoxide solvent molecule each one coordinated at one Co site and two water molecules coordinated on the Co sites opposite to the dimethylsulfoxide. The enantiopurity distinguishing parameters according to Parsons = 0.104(7) (determined with the SHELX software) and Hooft = 0.050(5) (determined with the PLATON software) were found to be strong.

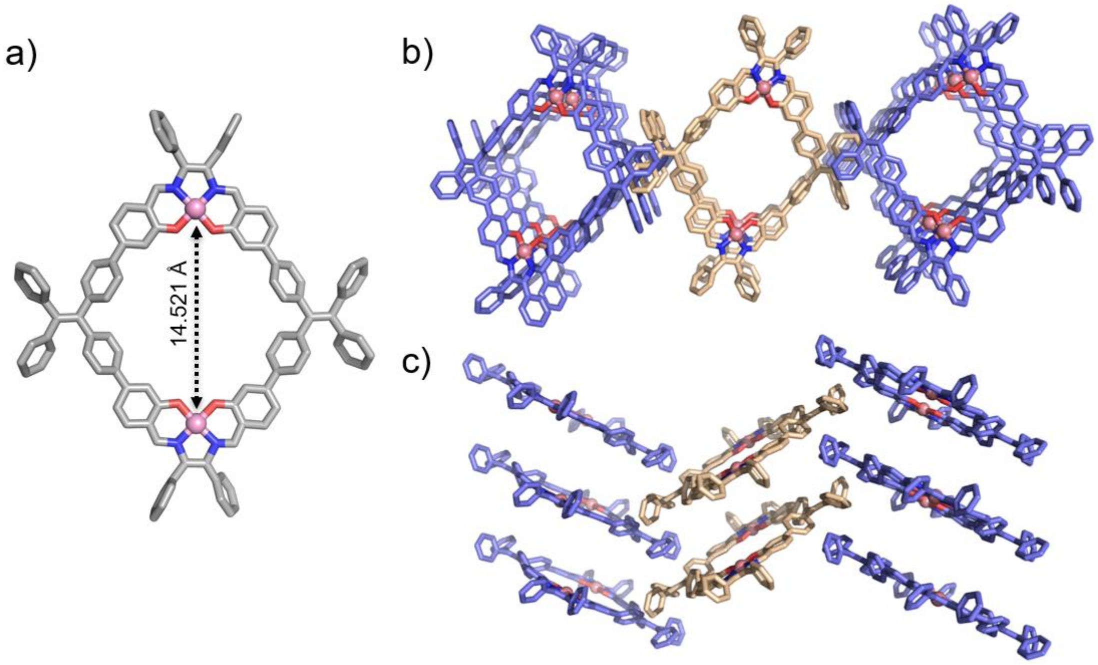

Figure S37. a) Single crystal X-ray structure and b) packing of ***s*-Co^{III}_2R** in top and c) in side view, showing a herring bone pattern of columns formed from displaced, stacked rings in the solid state (solvent molecules and hydrogens are not shown for clarity).

6. FT-IR spectroscopy

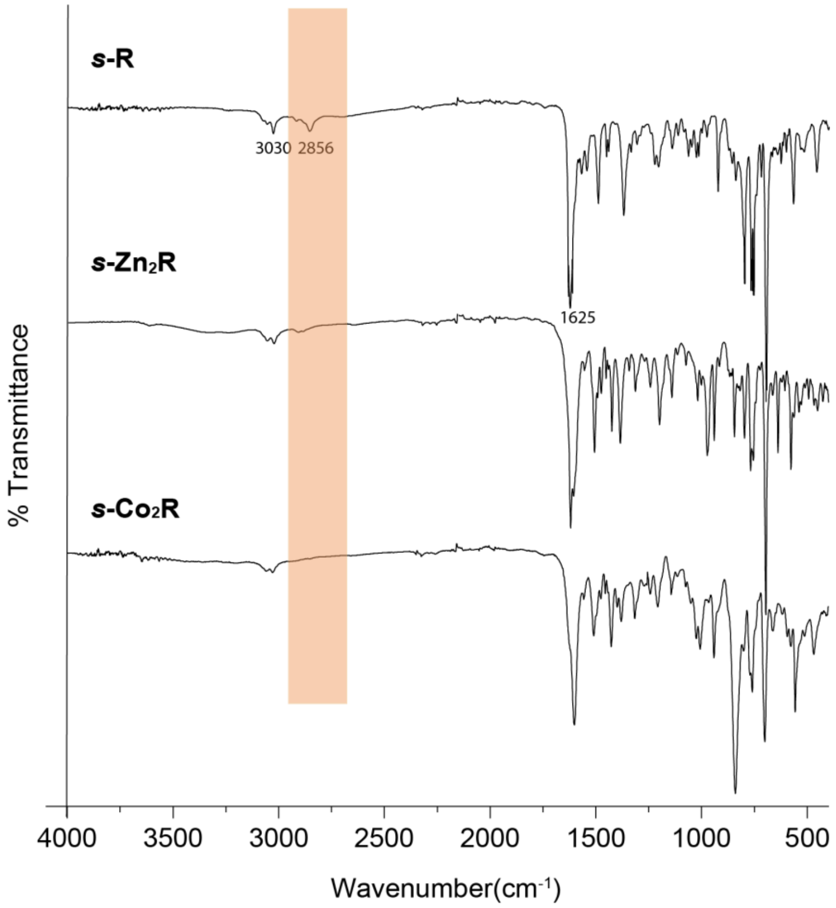

Figure S38. FT-IR spectra of **R**, **Zn_2R** and **Co(III)$_2$R** showing vanishing of the OH vibrational band at ν = 2856 cm^{-1} upon metal complexation.

7. UV-Vis Absorption spectroscopy

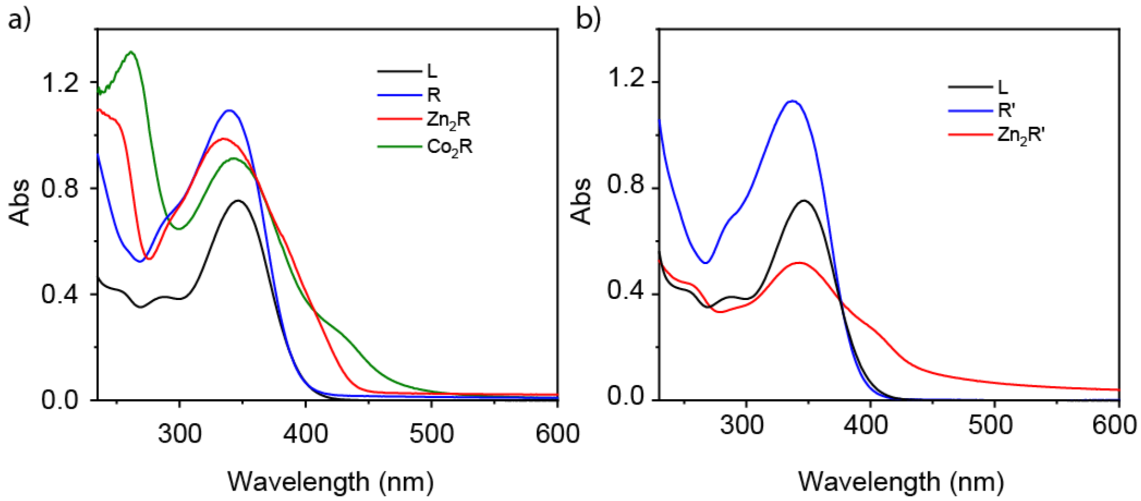

Figure S39. UV-Vis spectra of **L**, **R** and metal complexes obtained in THF at RT (c_L = 2 × 10^{-5} M, c_R = 1× 10^{-5} M, $c_{R'}$ = 1 × 10^{-5} M, c_{Zn2R} = 1 × 10^{-5} M, $c_{Zn2R'}$ = 1 × 10^{-5} M, cuvette path length 1 cm).

Table S2. Absorption values of compounds in THF

	L	R	R'	Zn_2R	Co^{III}_2R	Zn_2R'
λ_{max}/nm	347	339	337	334	343	343
Abs	0.75297	1.09278	1.1296	0.9869	0.91231	0.51853
$\varepsilon/(M^{1}cm^{1})$	75297	109278	112960	98690	91231	51853

8. Emission spectroscopy

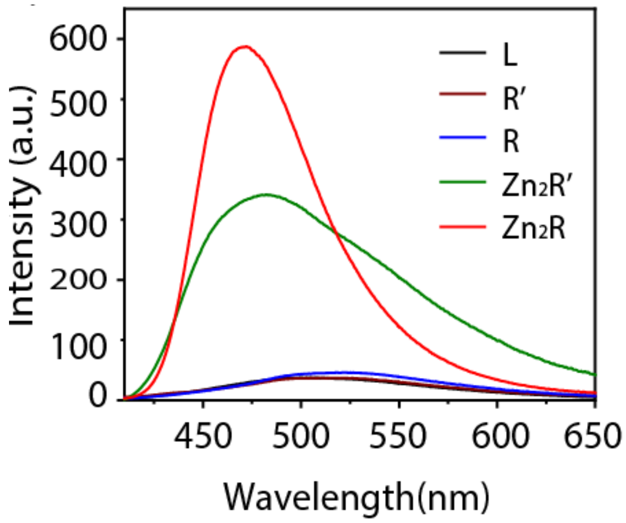

Figure S40. Emission spectra of ligand and macrocycles obtained in THF (RT, $c_L = 2\times10^{-5}$ M, $c_R = 1\times10^{-5}$ M, $c_{R'} = 1\times10^{-5}$ M, $c_{Zn2R} = 1\times10^{-5}$ M, $c_{Zn2R'} = 1\times10^{-5}$ M, cuvette path length 1 cm).

Table S3. Fluorescence data of the examined compounds.

	L	R'	R	Zn_2R'	Zn_2R
λ_{ex} (nm)	360	360	360	360	360
λ_{em} (nm)	500	525	525	480	470

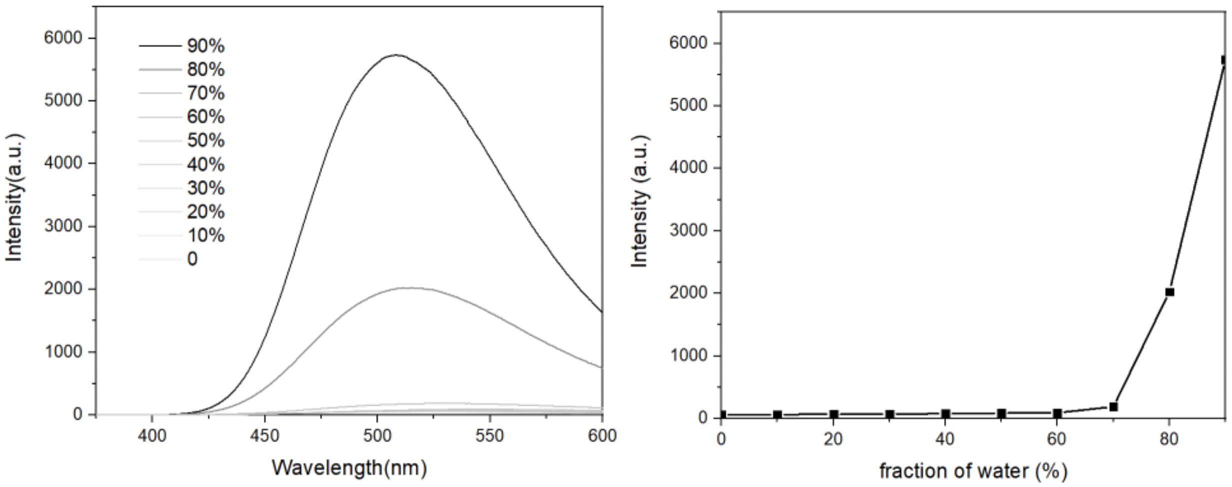

Figure S41. Emission spectra of **L** in THF/H_2O mixtures with different content of water.

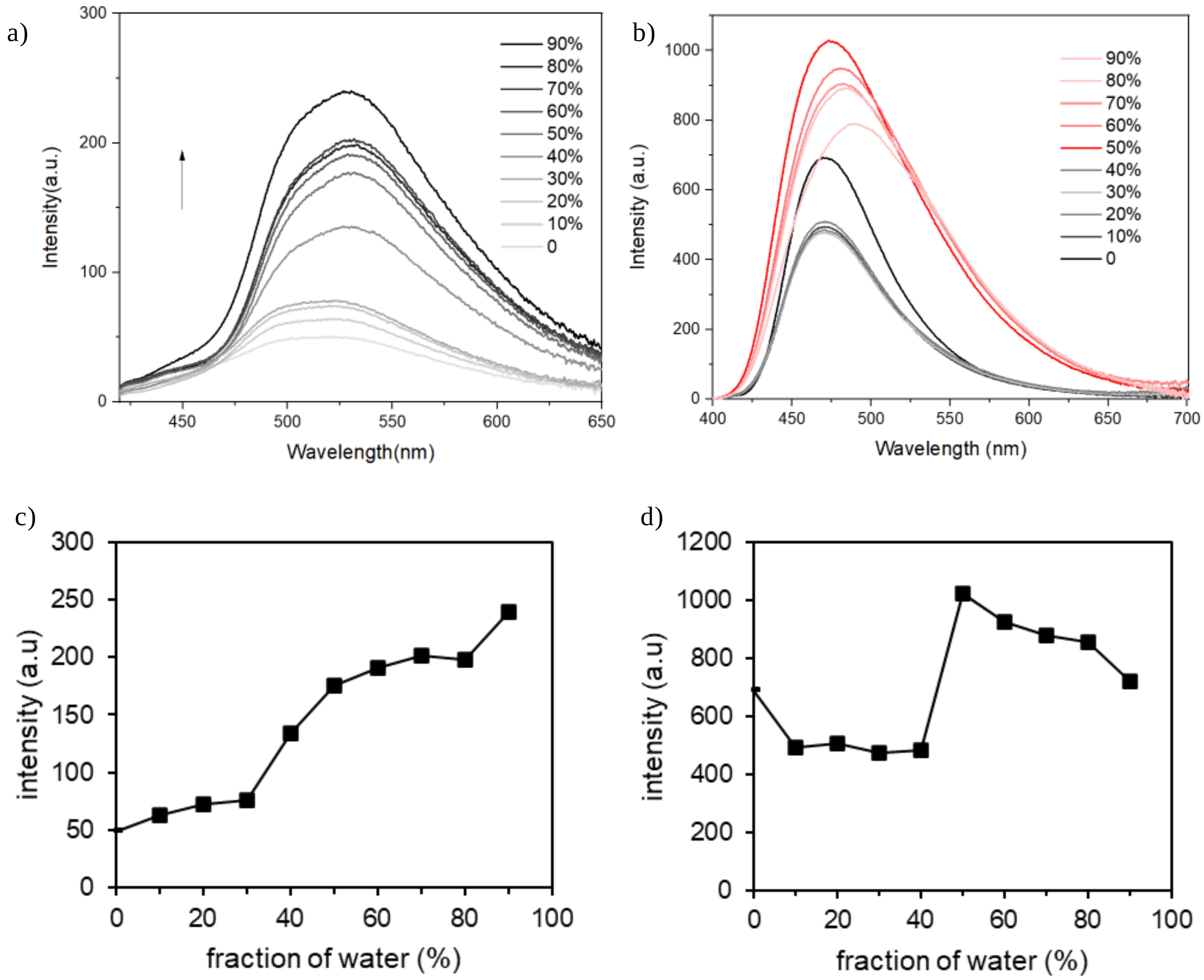

Figure S42. Emission spectra of a,c) **R** and b,d) **Zn_2R** in THF/H_2O mixtures with different content of water.

8.1 Quantum yield (QY) determination

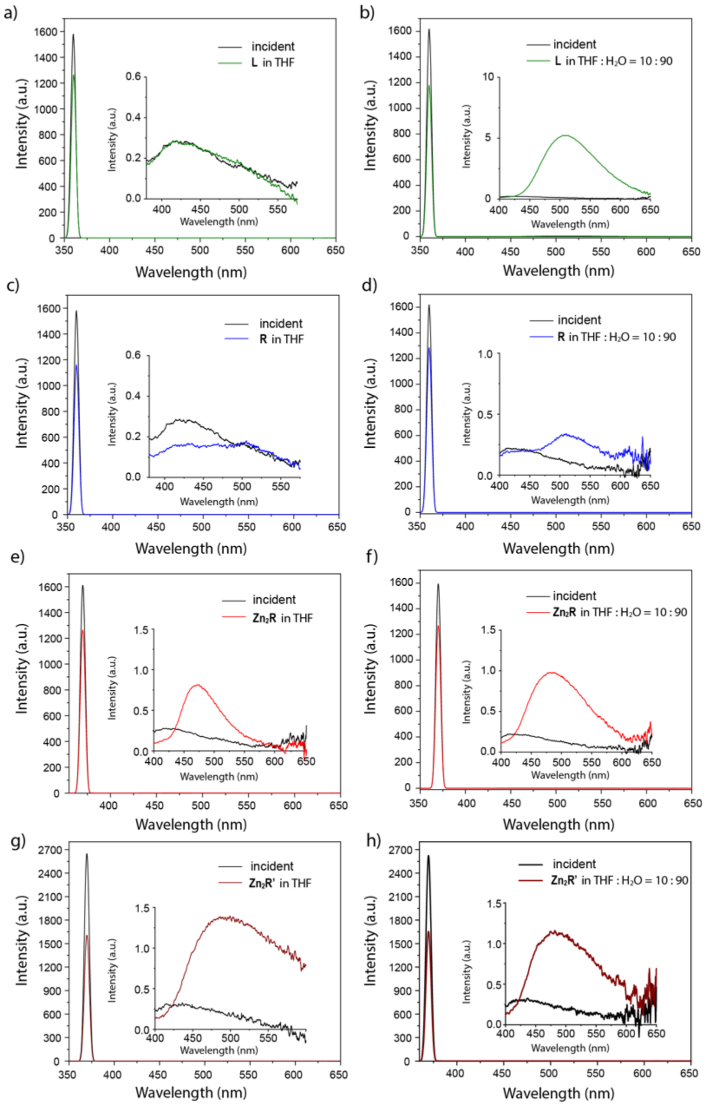

Figure S43. QY spectra of **L**, **R**, **Zn$_2$R** and **Zn$_2$R'** in THF and in THF: H_2O=10:90 (v : v) respectively.

Table S4. Luminescent quantum yields of the examined compounds (in %)

	Φ_F (in THF)	Φ_F (in THF: H_2O=10: 90)
**L**	1.86	21.23
**R**	1.11	2.70
**Zn$_2$R**	3.51	6.02
**Zn$_2$R'**	3.38	3.51

9. Chiroptical Spectroscopy

9.1 Circular Dichroism (CD) Spectra

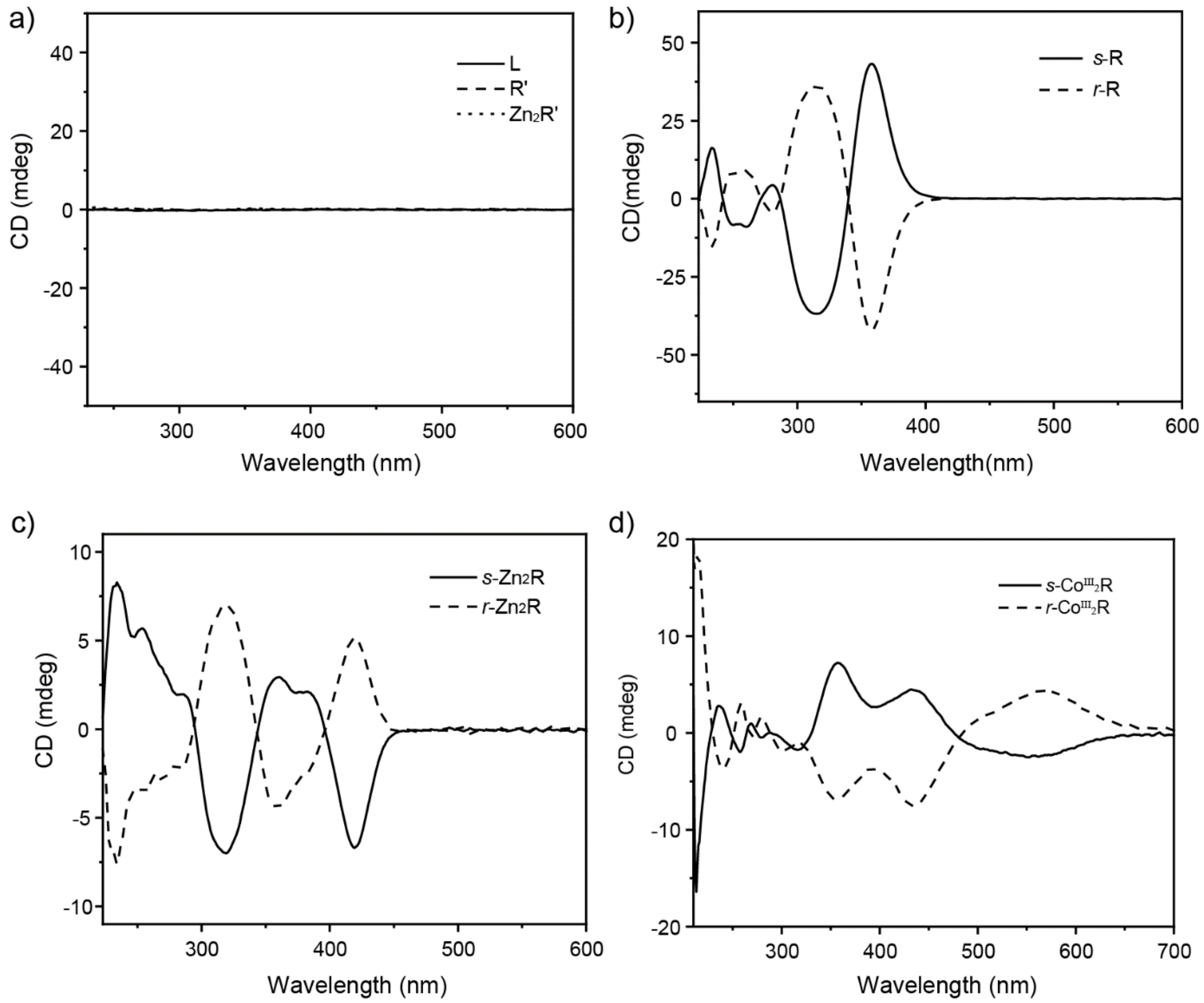

Figure S44. CD spectra of a) **L**, **R’**, **Zn_2R’** b) ***s-/r-* R** c) ***s-/r-* Zn_2R** and d) ***s-/r-* Co^{III}_2R** in THF (c = 1 × 10^{-5} M, 298 K).

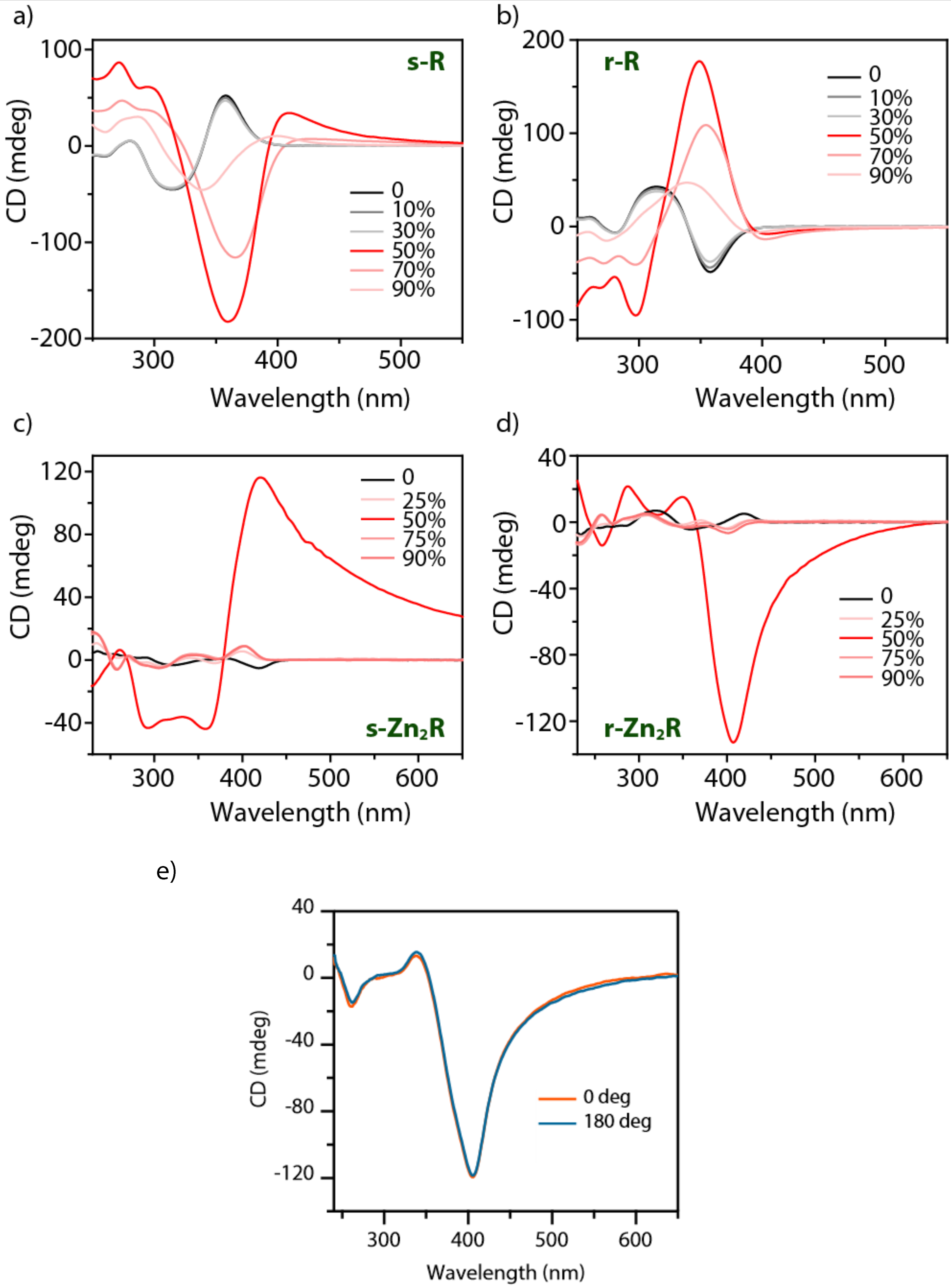

Figure S45. CD spectra of (a) ***s*-R** (b) ***r*-R** (c) ***s*-Zn₂R** (d) ***r*-Zn₂R** in THF with different fractions of water (c = $1x10^{-5}$ M, cuvette path length: 1 cm, 298 K). (e) CD spectra of ***r*-Zn₂R** obtained with cuvette position of 0° and 180° to test linear dichroism interference.

9.2 Circularly Polarized Luminescence (CPL)

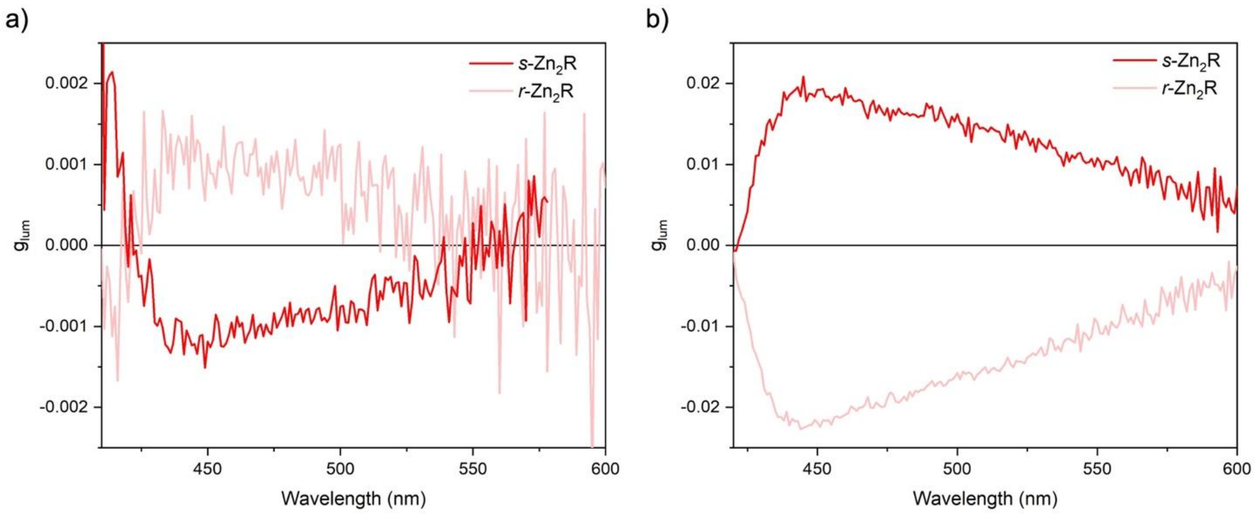

Figure S46. CPL (g_{lum}) spectra of ***s-/r-* Zn$_2$R** a) in THF and b) in THF:H_2O = 50:50 (λ_{ex} = 370 nm, 298 K).

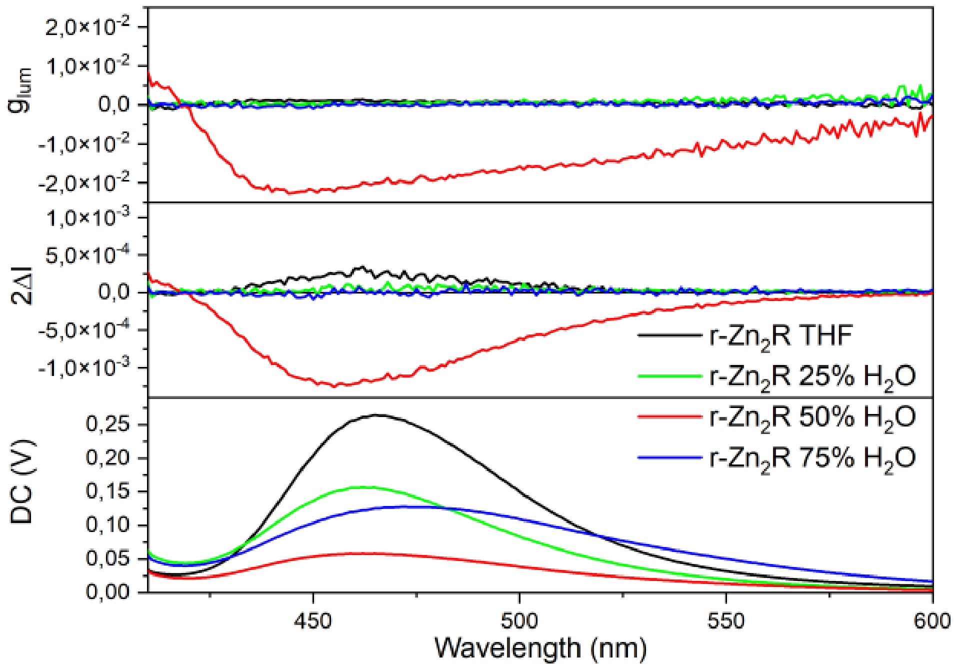

Figure S47. CPL spectra of ***r-* Zn$_2$R** in in THF:H_2O with different fractions of water 0, 25, 50 and 75% (λ_{ex} = 370 nm, 298 K).

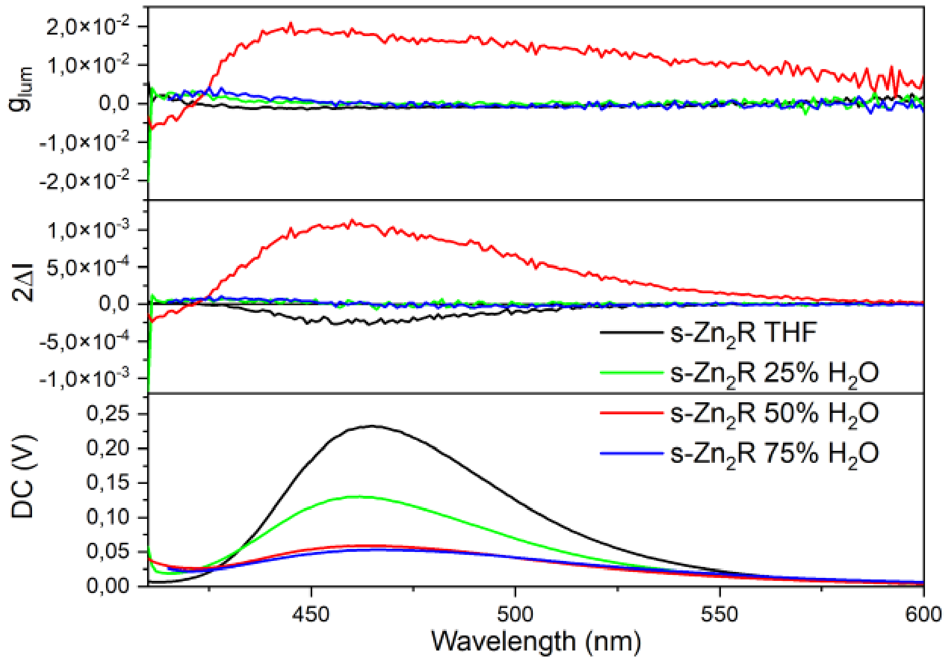

Figure S48. CPL spectra of ***s***- **Zn₂R** in in THF:H₂O with different fractions of water 0, 25, 50 and 75% (λ_{ex} = 370 nm, 298 K).

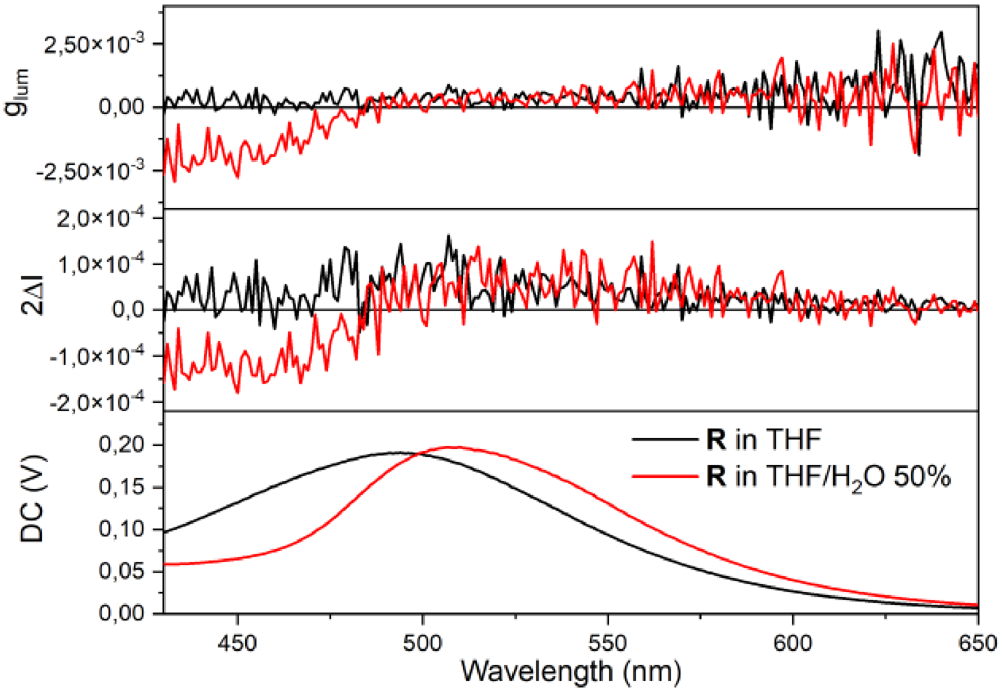

Figure S49. Accumulated CPL spectra of ***r*-R** in in THF (20 spectra averaged) and THF/H_2O 50% (25 spectra averaged; λ_{ex} = 370 nm, 298 K). The structurally highly flexible non-metallated ring does not show a CPL effect when fully dissolved in THF and only a slight effect (affected by chemical instability under irradiation, see next figure) in a THF/H_2O 50% mixture.

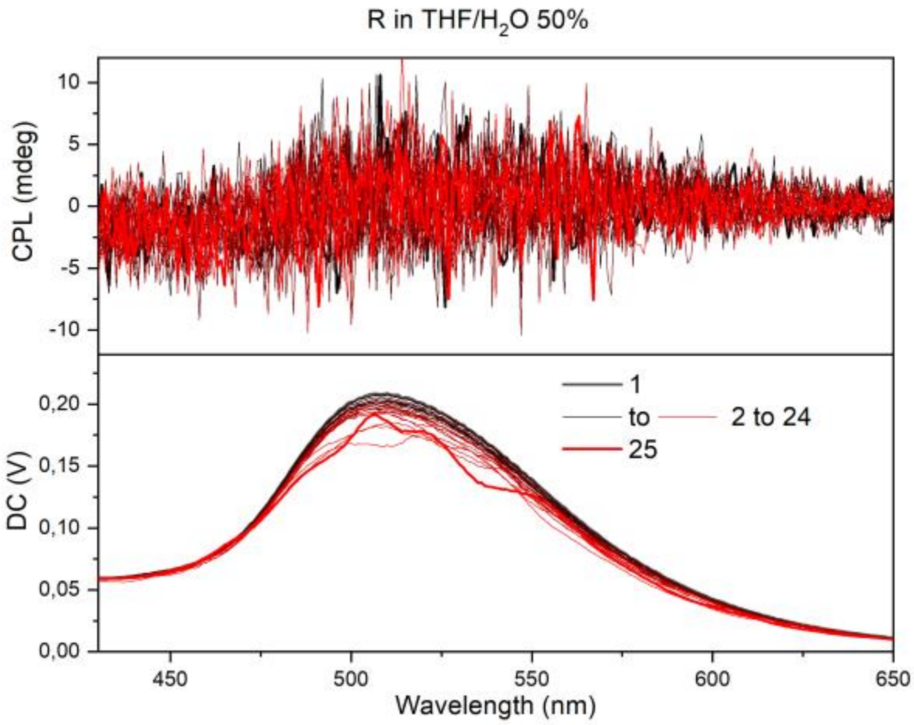

Figure S50. Stack of single emission and CPL spectra of ***r*-R** in THF/H_2O 50% showing instability of the sample under continuous irradiation over time (λ_{ex} = 370 nm, 298 K).

10. Dynamic Light Scattering (DLS) experiments

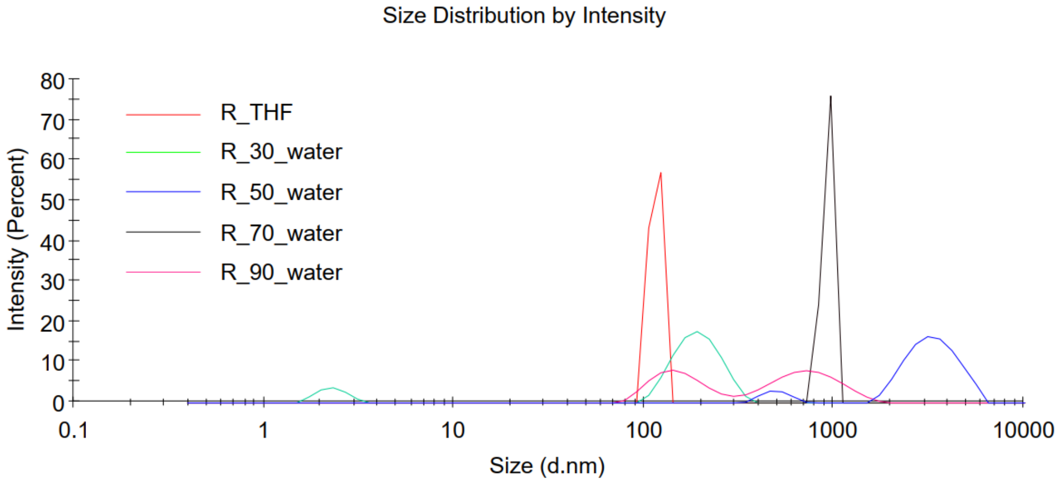

Figure S51. DLS distributions of **R** in THF and water with different fractions of water.

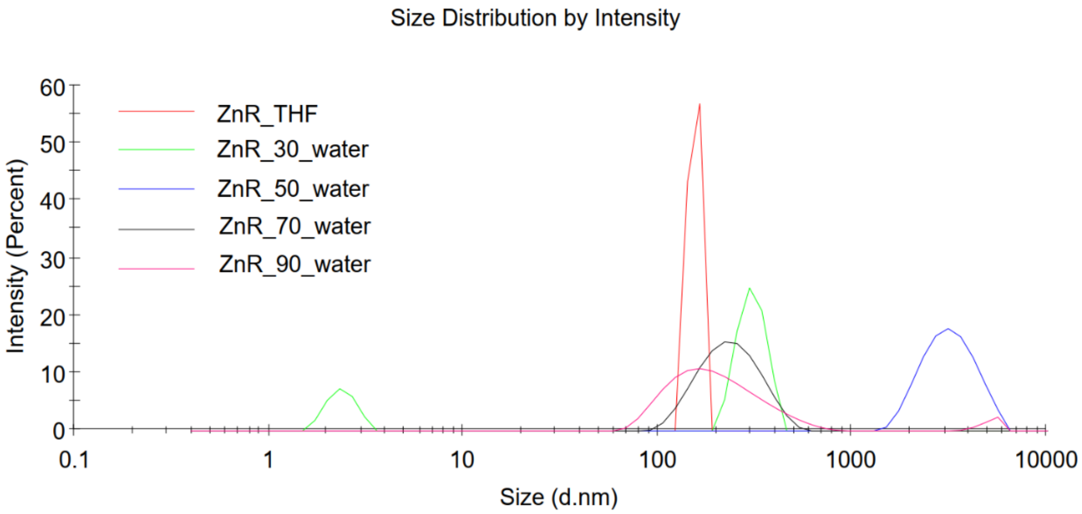

Figure S52. DLS distributions of **Zn_2R** in THF and water with different fractions of water.

11. SEM and EDS Electron Microscopy Results

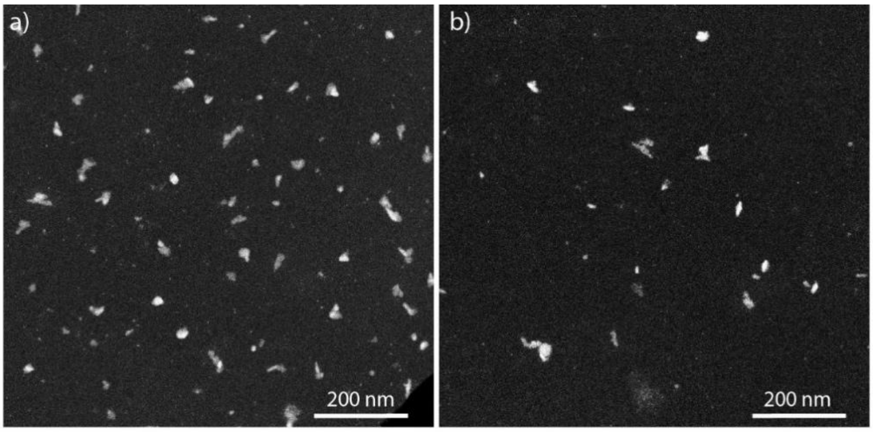

Figure S53. SEM image of ***s*-R** and ***s*-Zn_2R** deposited from THF.

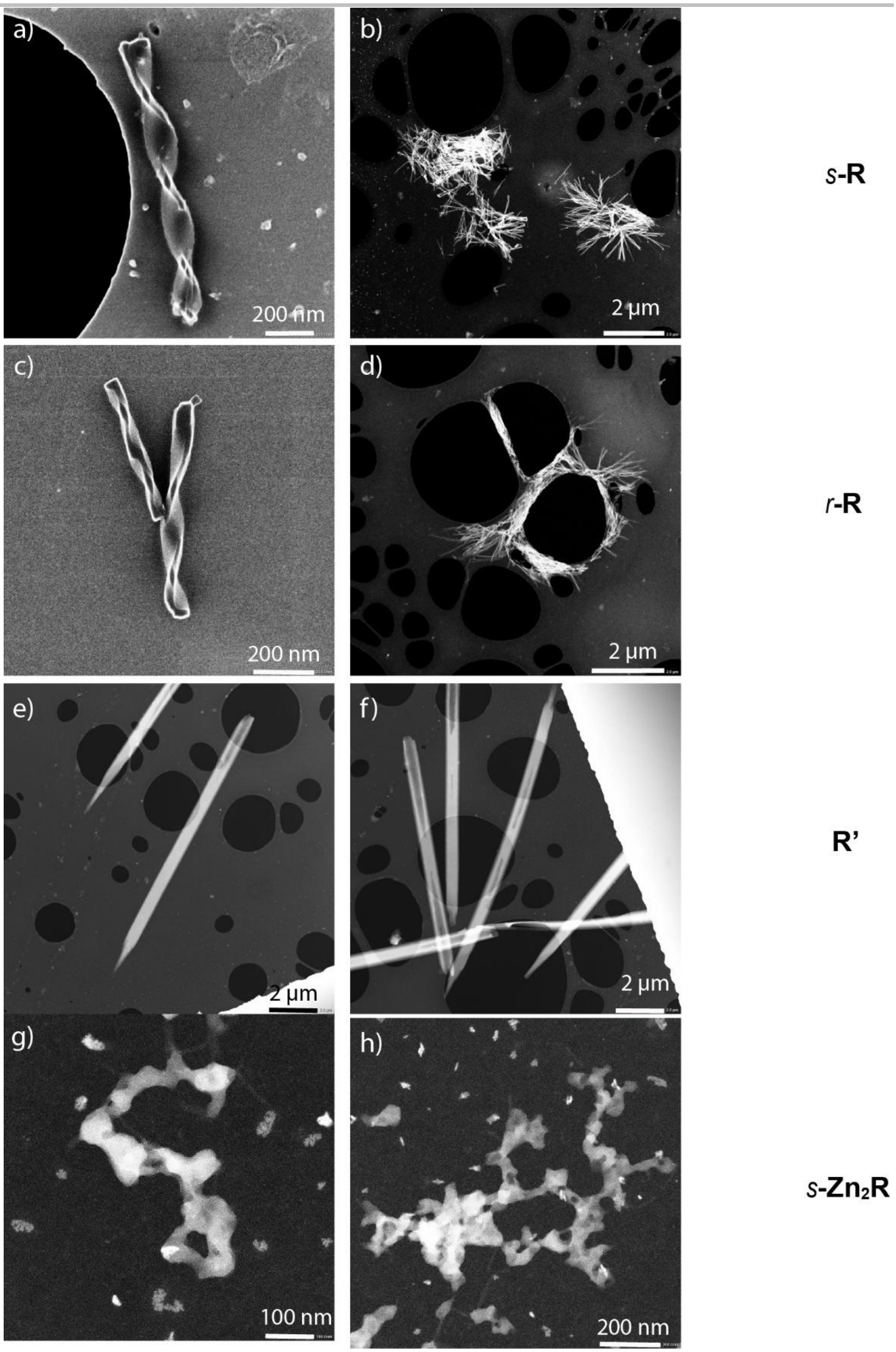

Figure S54. SEM images of compounds deposited from THF:H_2O=50:50.

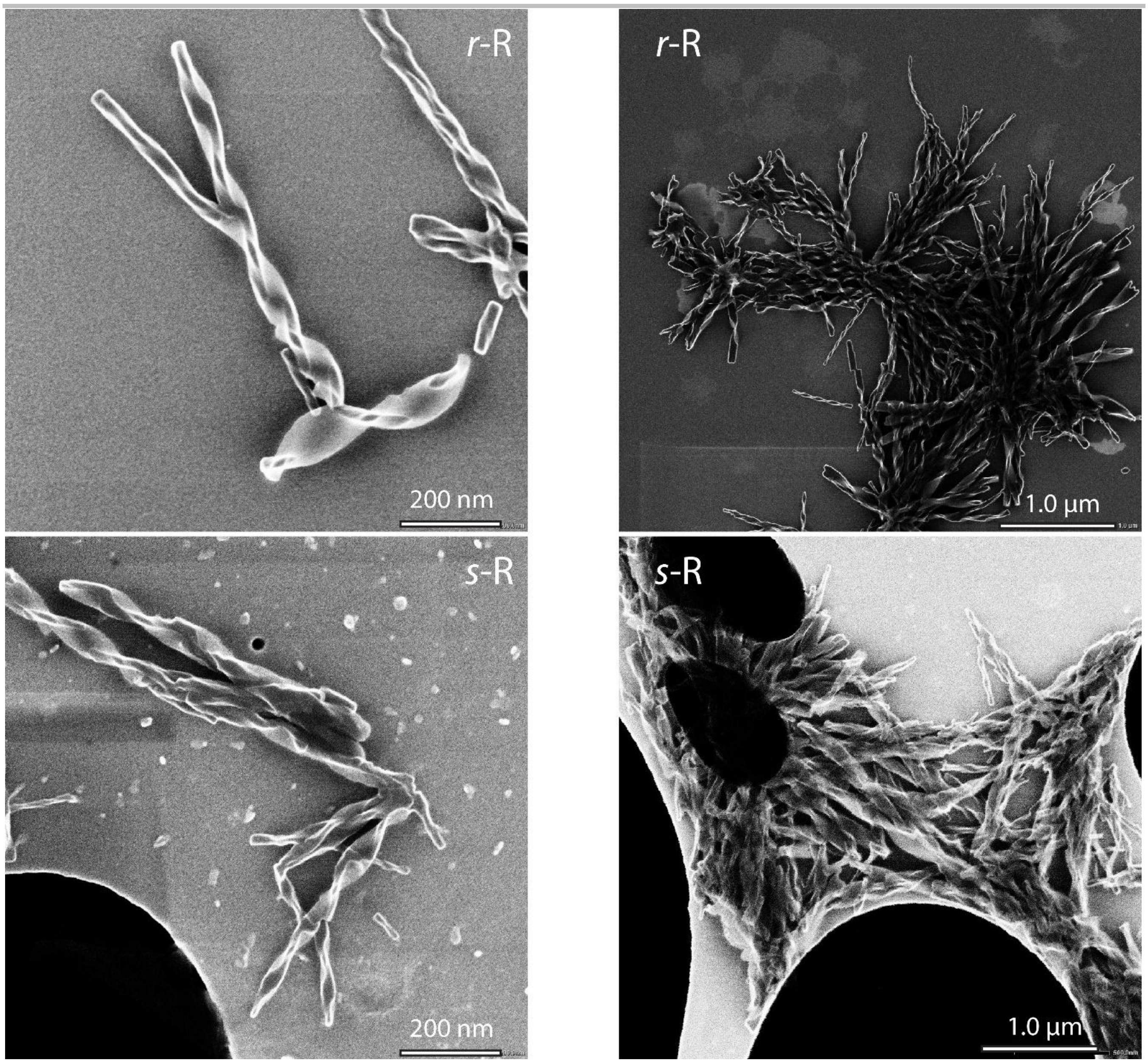

Figure S55. Further SEM images of compounds deposited from THF:H_2O=50:50. Note the opposite handedness of the curled fibers formed from ***r*-R** and ***s*-R**. In images prepared from the same sample batches, helices with a variety of different pitches can be observed, probably due to different stages of growth and local differences in concentration (i.e. it seems that thinner fibers are more strongly twisted and wider fibers are less twisted along their long axis, as best observable in the upper right figure).

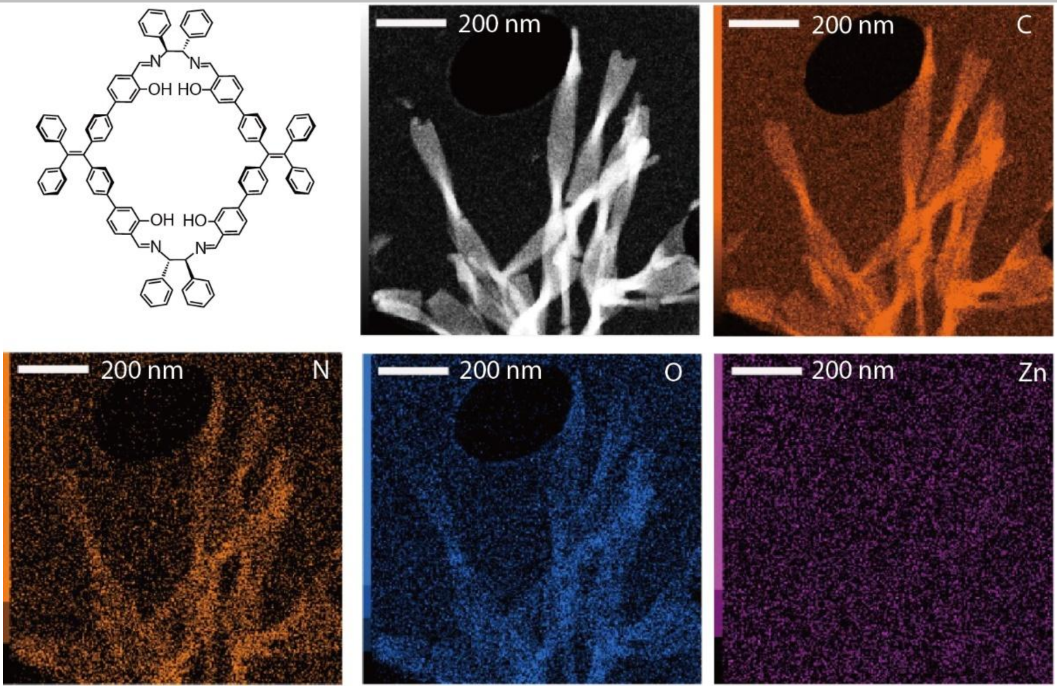

Figure S56. EDS mapping of ***s*-R** with element mapping of C, N, O and Zn.

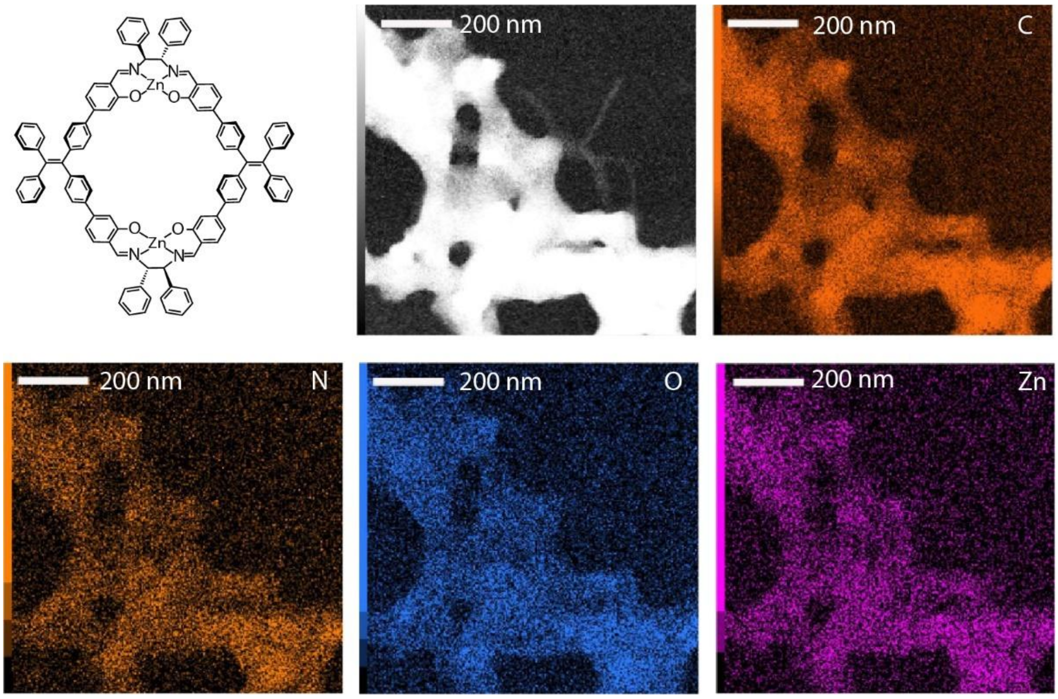

Figure S57. EDS mapping of ***s*-Zn$_2$R** with element mapping of C, N, O and Zn.

12. Theoretical Calculations

In order to understand both the chiroptical behavior of the THF-solvated and aggregated rings as well as the plausible molecular models of the aggregated rings assembled into columnar stacks, we performed a series of computations.

First, we examined the conformational preference of the Zn-free and Zn-coordinated rings with *r,r*- (or *s,s*-) stereochemistry at the diphenyl-ethylenediamine parts by geometry-optimizations (ORCA 5 program[13] using the r^{2}SCAN-3c[14] method, followed by single point energies on ωB97M-V/def2-TZVP level of theory using the CPCM solvent model for THF – Table S5). Therefore, starting structures were prepared with both phenyl substituents in axial positions as well as the ring-flipped isomer with both phenyls in equatorial positions. In addition, each isomer was calculated with the TPE units either in a *PP* or *MM* chiral twist. The computations showed, that – for **Zn$_2$R** – the lowest energy structure in solution always carries all phenyl substituents in axial positions, with the lowest energies obtained for axial ***r*-Zn$_2$R** with TPEs in *MM* configuration (and axial ***s*-Zn$_2$R** with TPEs in *PP* configuration for the mirror image case). Both equatorial conformers with TPEs in *MM* or *PP* twist were always of higher energy as the respective axial conformers for **Zn$_2$R** (Table S6). For more structurally flexible metal-free ring **R**, however, the equatorial isomers were more stable, in particular the equatorial ***r*-R** isomer with TPEs in *MM* configuration (Table S6).

Next, we computed the CD spectra by TD-DFT calculations on BHandHLYP/def2-SVP level of theory in Orca 5[13] and Gaussian 16[15] (Figure S59).

In order to get insight into plausible molecular arrangements in the TEM-observed fibers obtained from the THF:water = 1:1 mixtures, we placed three flat rings of ***r*-R** (without coordinated metal) and of ***r*-Zn$_2$R** in a stack in a way that the TPE-units are on top of each other as well as the salen units. For both stacks, the phenyl substituents on the ethylene diamine parts were arranged in equatorial positions. The stacks were then optimized using the GFN2-xTB[16] method.

To get additional insight into the structure and properties of stacked aggregates, the dimeric stacks of ***r*-R** and ***r*-Zn$_2$R** were isolated and optimized at r^{2}SCAN-3c level. Noteworthy, the optimization has not altered stack twist directions observed for GFN2-xTB structures. Then we calculated the CD spectra by TD-DFT calculations on BHandHLYP/def2-SVP level (Figure 8d) as well as single point energies on ωB97M-V/def2-TZVP level of theory using the CPCM solvent model for THF (Table S5). The latter allowed us to estimate the energetic from ring stacking of ***r*-R** and ***r*-Zn$_2$R** (Table S6).

To study the nature of single-electron excitation transitions and associated with them charge transfers for the lowest energy monomers and dimers of ***r*-R** and ***r*-Zn$_2$R**, the hole-electron distribution analysis[17] was performed using Multiwfn 3.8 software.[18] The latter method describes where the excited electron leaves as "hole" (blue on Figure S61) and arrives as "electron" (red on Figure S61).

Table S5. Energies (Harteree) of monomers and dimers metalated and metal-free ring conformers (eq./ax. refers to the phenyl substituents in the ethylenediamine part; *PP* and *MM* refers to the twisting sense of the TPE units; r^{2}SCAN-3c optimized, ωB97M-V/def2-TZVP energies in CPCM solvent THF).

Complex	***r*-R**		***r*-Zn$_2$R**		***s*-Zn$_2$R**	
monomer	MM	PP	MM	PP	MM	PP
eq.	–4683.707699	–4683.693013	–8240.076800	–8240.081488	–8240.081488	–8240.076800
ax.	–4683.690525	–4683.682203	–8240.086688	–8240.081902	–8240.081902	–8240.086688
dimer	–9367.459868		–16480.296648		–	

Table S6. Relative energies (kJ/mol) of monomers and dimers metalated and metal-free ring conformers (eq./ax. refers to the phenyl substituents in the ethylenediamine part; *PP* and *MM* refers to the twisting sense of the TPE units; r^{2}SCAN-3c optimized, ωB97M-V/def2-TZVP energies in CPCM solvent THF).

Complex	***r*-R**		***r*-Zn$_2$R**		***s*-Zn$_2$R**	
monomer	MM	PP	MM	PP	MM	PP
eq.	0.0	38.6	26.0	13.7	13.7	26.0
ax.	45.1	66.9	0.0	12.6	12.6	0.0
Dimerization energies ($E_{dim} = E_D - 2E_M$)						
E_{dim}	-142.6		-323.6		–	

eq. ***s*-Zn$_2$R** with *PP* TPE

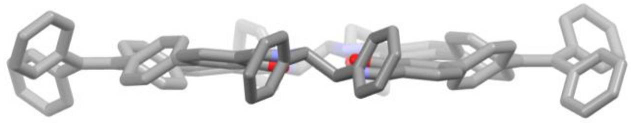

eq.l ***s*-Zn$_2$R** with *MM* TPE

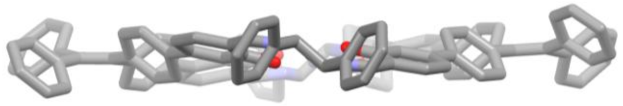

ax. ***s*-Zn$_2$R** with *PP* TPE

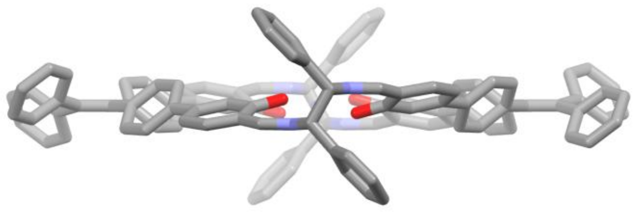

ax. ***s*-Zn$_2$R** with *MM* TPE

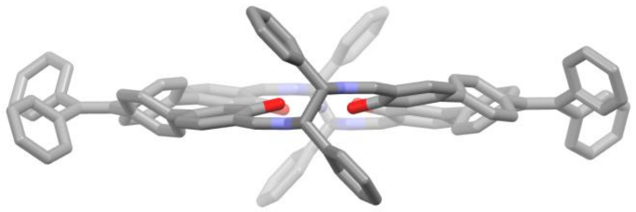

Figure S58. Optimized structures of the four ***s*-Zn$_2$R** conformers (view on diphenyl-salen part). According to the calculations, the energetically lowest conformer in solution is ax. ***s*-Zn$_2$R** with *PP* TPE (lower left).

a)

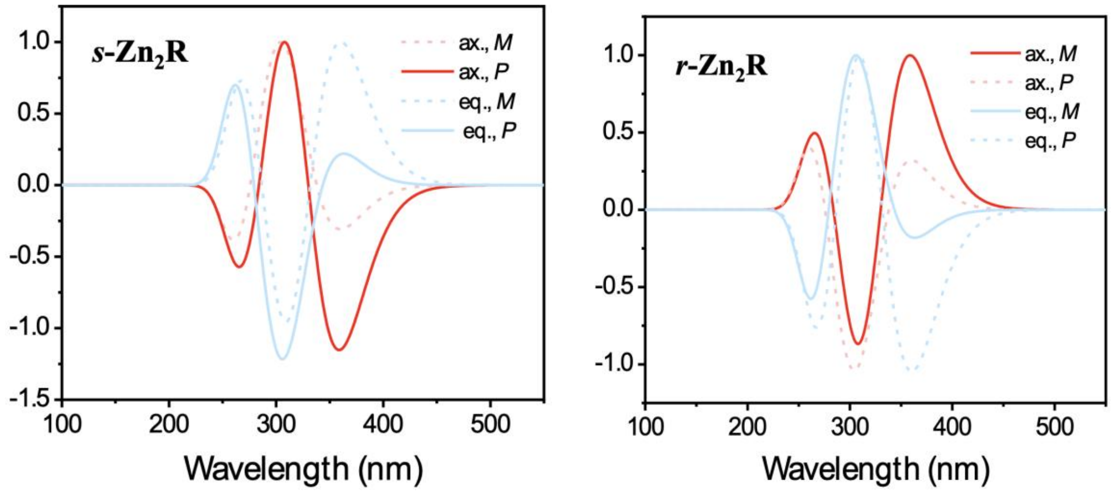

b)

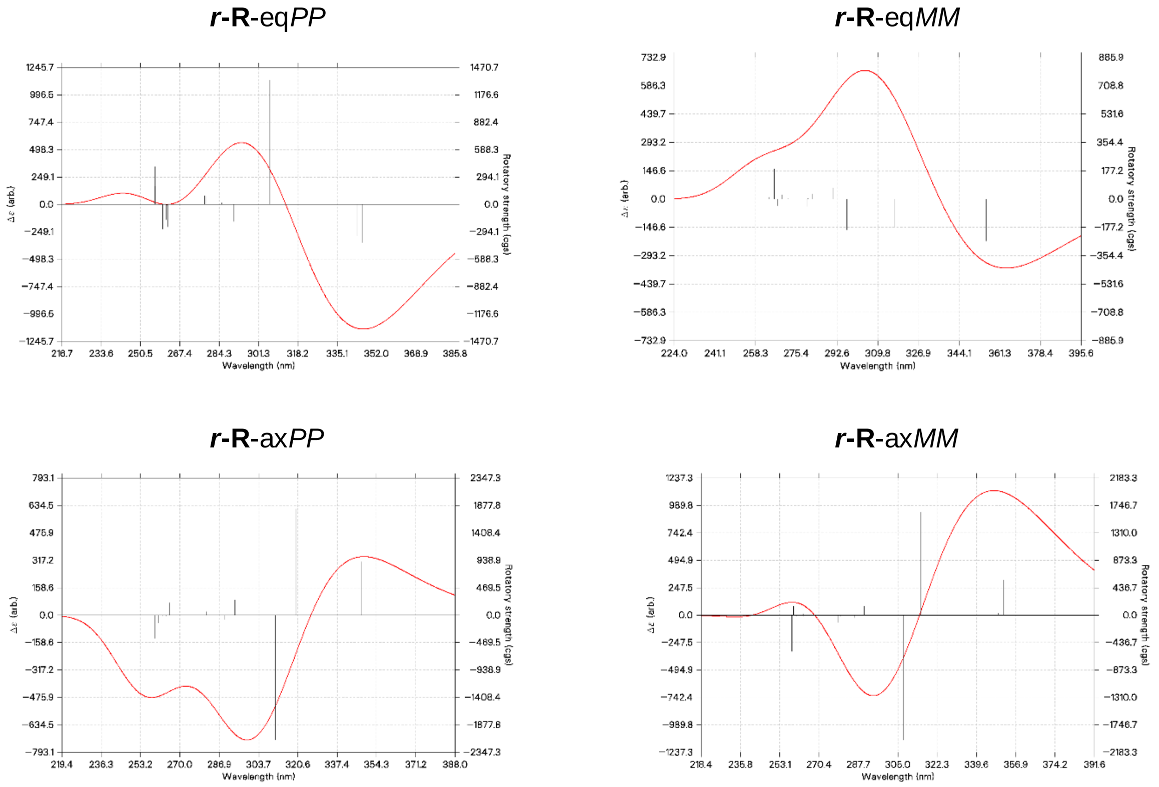

Figure S59. Computed CD spectra for all four conformers: a) per ring enantiomer for **Zn$_2$R** (based on structures shown above, TD-DFT: BHandHLYP/def2-SVP, CPCM solvent THF in Orca[13]) and b) for the four conformers of ***r*-R** (plotted using Multiwfn[15]). The results indicate that the sign of the Cotton effect is governed by the eq./aq. conformation of the diphenyl salen units, not by the TPE twist. The sign of the longest wavelength CD band correlates to the eq or ax conformation of the diphenyl-ethylenediamine parts of ***r*-R** and **Zn$_2$R** in the same way.

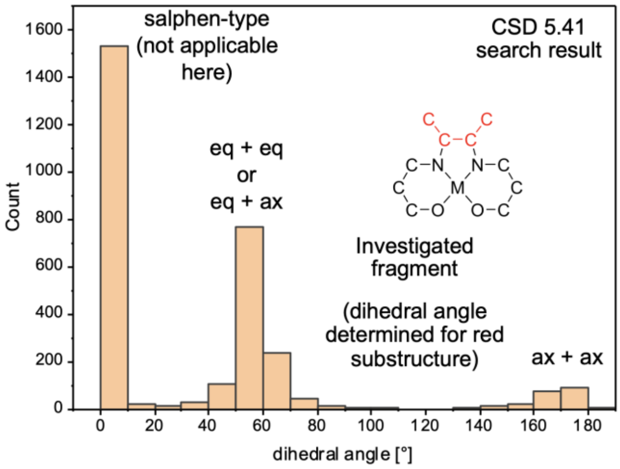

Figure S60. Crystal structure database (CSD) search results for a salen-derived fragment with two subsituent at the ethylenediamine-derived unit. Plotted is the count of found solid state structures over the dihedral angle between the two substituents. Angles close to 0° are not considered as they all belong to salphen-type compounds (all red atoms part of an aromatic ring). The other results show a strong prevalence for substituents in equatorial position in the solid-state structures, in agreement with the X-ay structures of the complexes. We hence expect the rings to adopt an equatorial conformation in columnar stacks within the TEM-observed fibers.

TD-DFT transition data of the ax. ***s*-Zn$_2$R** isomer with *PP* TPE conformation (BHandHLYP/def2-SVP, CPCM solvent THF):

```
Excited State   1:      Singlet-A      3.5591 eV  348.36 nm  f=2.5177  <S**2>=0.000
     418 -> 426        -0.16940
     419 -> 427         0.11345
     420 -> 425         0.19739
     421 -> 424        -0.39843
     421 -> 428         0.12674
     422 -> 423         0.42009
     422 -> 427        -0.12861
This state for optimization and/or second-order correction.
Total Energy, E(TD-HF/TD-DFT) =  -8233.32075222
Copying the excited state density for this state as the 1-particle RhoCI density.

Excited State   2:      Singlet-A      3.6127 eV  343.19 nm  f=0.0000  <S**2>=0.000
     418 -> 425         0.12564
     420 -> 426        -0.12811
     421 -> 423        -0.42803
     421 -> 427         0.17535
     422 -> 424         0.43492
     422 -> 428        -0.15187

Excited State   3:      Singlet-A      3.6477 eV  339.89 nm  f=0.0306  <S**2>=0.000
     417 -> 426        -0.25832
     418 -> 424         0.26849
     418 -> 428         0.13596
     419 -> 425         0.27770
     420 -> 423         0.31448
     420 -> 427         0.14049
     421 -> 426         0.21124
     422 -> 425         0.25972

Excited State   4:      Singlet-A      3.7836 eV  327.68 nm  f=0.1566  <S**2>=0.000
     417 -> 424         0.28118
     417 -> 428         0.10823
     418 -> 426        -0.27579
     419 -> 423         0.32030
     420 -> 425         0.30320
     421 -> 424         0.18157
     421 -> 428        -0.14373
     422 -> 423        -0.14331
     422 -> 427         0.16383
```

```
Excited State   5:      Singlet-A      3.7861 eV  327.47 nm  f=1.7439  <S**2>=0.000
     417 -> 425        0.29718
     418 -> 423        0.30891
     418 -> 427        0.13137
     419 -> 426       -0.27066
     420 -> 424        0.29618
     420 -> 428        0.13685
     421 -> 425       -0.20711
     422 -> 426       -0.22487

Excited State   6:      Singlet-A      3.8693 eV  320.43 nm  f=0.0000  <S**2>=0.000
     417 -> 423        0.32480
     417 -> 427        0.11807
     418 -> 425        0.32813
     419 -> 424        0.30767
     419 -> 428        0.11249
     420 -> 426       -0.30575
     421 -> 427       -0.11379
     422 -> 428        0.11758

Excited State   7:      Singlet-A      4.0435 eV  306.63 nm  f=0.0015  <S**2>=0.000
     414 -> 425        0.10606
     415 -> 424        0.19066
     416 -> 423       -0.20867
     417 -> 426        0.20344
     418 -> 424       -0.12137
     419 -> 425       -0.22538
     420 -> 423       -0.11303
     421 -> 426        0.33186
     422 -> 425        0.34338

Excited State   8:      Singlet-A      4.0917 eV  303.01 nm  f=0.9741  <S**2>=0.000
     415 -> 423       -0.18962
     416 -> 424        0.18769
     417 -> 425        0.19336
     418 -> 423        0.10953
     419 -> 426       -0.18922
     421 -> 425        0.37828
     422 -> 426        0.35352

Excited State   9:      Singlet-A      4.5663 eV  271.52 nm  f=0.8067  <S**2>=0.000
     413 -> 424        0.17832
     414 -> 423        0.21028
     415 -> 426       -0.25654
     416 -> 425       -0.29626
     421 -> 428       -0.28152
     422 -> 423        0.11892
     422 -> 427        0.30892

Excited State  10:      Singlet-A      4.6221 eV  268.24 nm  f=0.0000  <S**2>=0.000
     413 -> 423       -0.18867
     414 -> 424       -0.18093
     415 -> 425       -0.27068
     416 -> 426       -0.26155
     421 -> 423        0.13289
     421 -> 427        0.31608
     422 -> 428       -0.29860

Excited State  11:      Singlet-A      4.6613 eV  265.99 nm  f=0.0002  <S**2>=0.000
     393 -> 424       -0.12039
     394 -> 426        0.14551
     417 -> 424       -0.25045
     417 -> 428       -0.10987
     418 -> 426        0.28122
     419 -> 423        0.24490
     420 -> 425        0.25653
     421 -> 424        0.22292
     422 -> 423        0.24171

Excited State  12:      Singlet-A      4.6645 eV  265.80 nm  f=0.0000  <S**2>=0.000
     393 -> 423        0.11857
     394 -> 425        0.13621
     417 -> 423        0.23717
     417 -> 427        0.10910
     418 -> 425        0.25752
     419 -> 424       -0.26232
     420 -> 426        0.28700
     421 -> 423       -0.20769
     422 -> 424       -0.25624
     422 -> 428       -0.10980
```

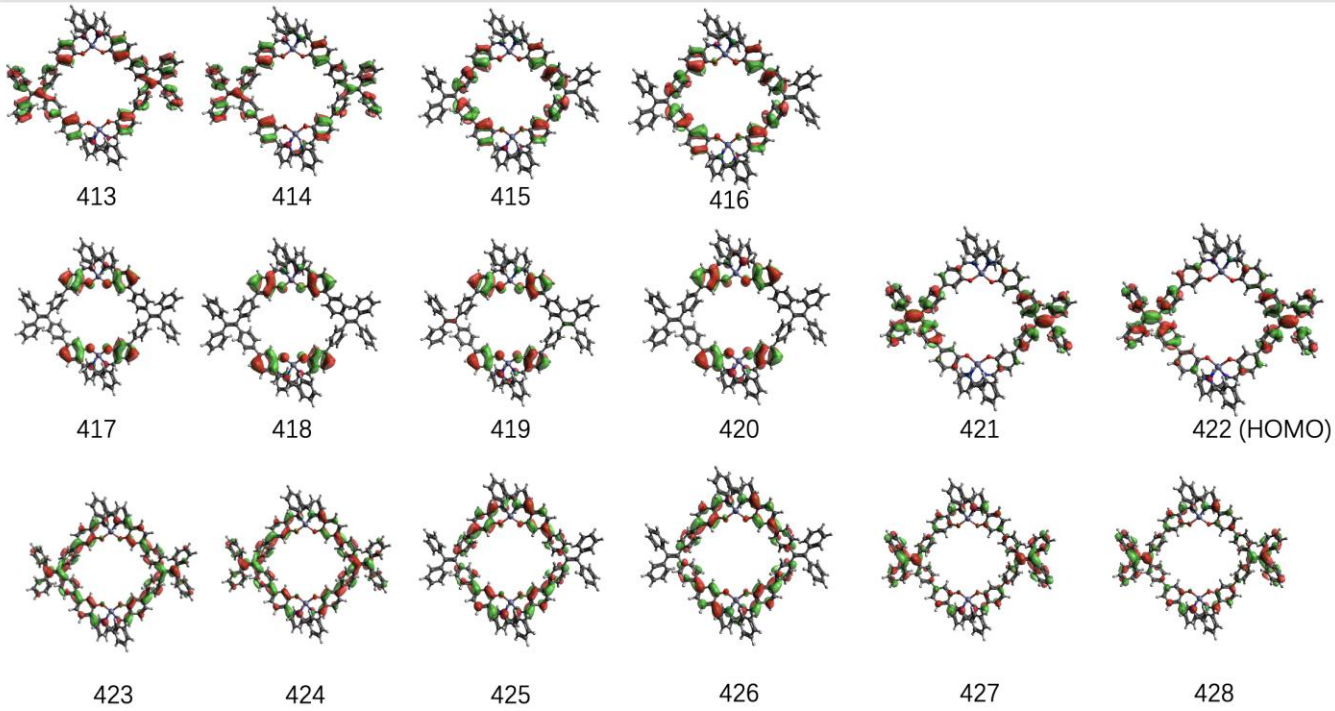

Figure S61. Orbital representations of some selected frontier orbitals

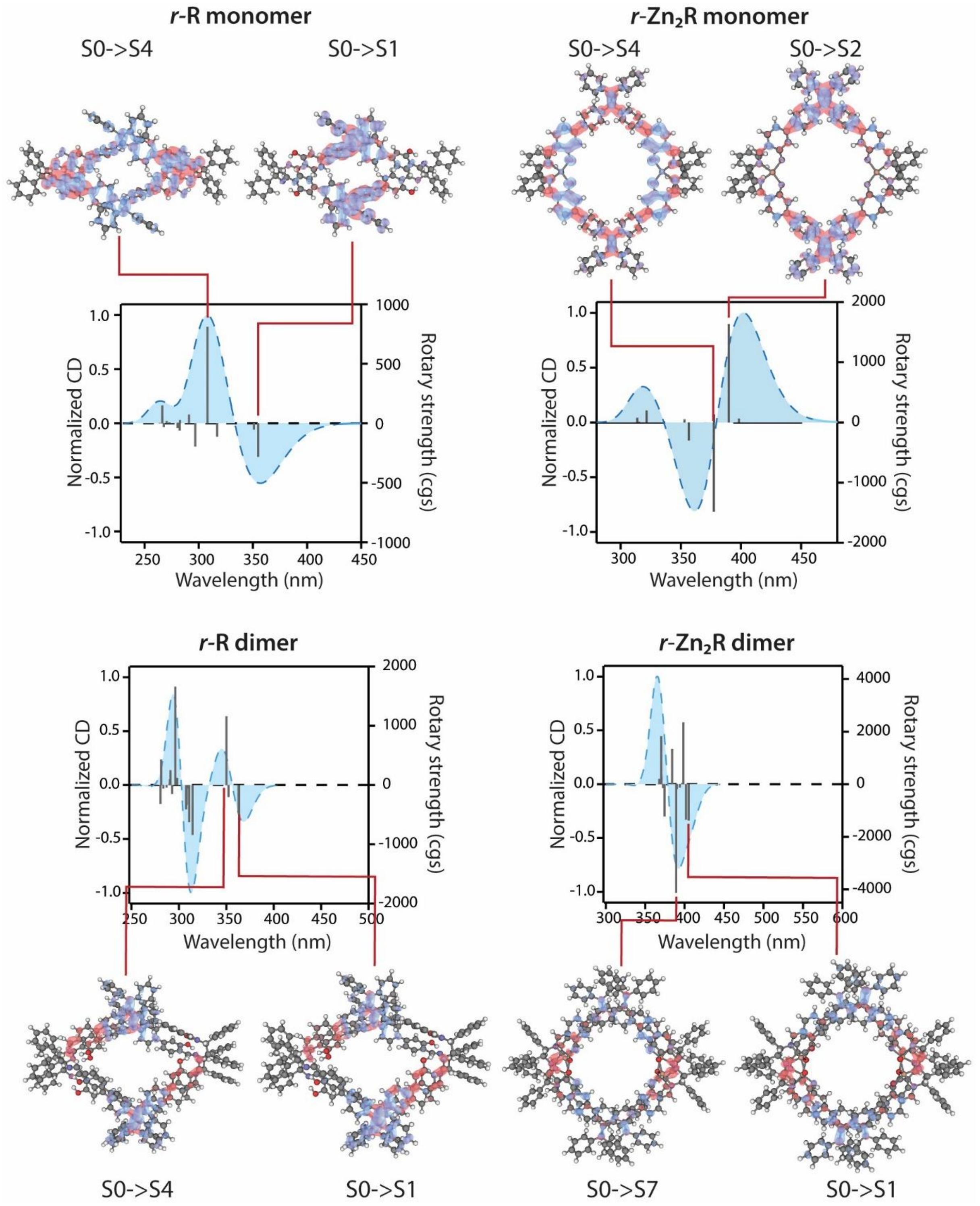

Figure S58. Calculated (grey area) absorption spectrum and oscillator strengths of the lowest energy monomers and dimers of ***r*-R** and ***r*-Zn$_2$R** together with the hole-electron distribution isosurfaces (isovalue 0.0005) for the selected transitions (hole distribution: blue; electron distribution: red).

13. References

[1] F. Mamiya, N. Ousaka, E. Yashima *Angew. Chem. Int. Ed*. **2015**, *54*, 14442.

[2] X. Zhou, H. Li, Z. Chi, X. Zhang, J. Zhang, B. Xu, Y. Zhang, S. Liu, J. Xu *New. J. Chem.* **2011**, *36*, 685.

[3] A. Burkhardt, T. Pakendorf, B. Reime, J. Meyer, P. Fischer, N. Stübe, S, Panneerselvam, O. Lorbeer, K. Stachnik, M. Warmer *Eur. Phys. J. Plus.* **2016**, *131*, 56.

[4] W. Kabsch *Acta Crystallogr. D Biol. Crystallogr*. **2010**. *66*, 125.

[5] G. M. Sheldrick *Acta Crystallogr. Sect. Found. Adv.* **2015***, 71*, 3.

[6] G. M. Sheldrick *Acta Crystallogr. C Struct. Chem.* **2015**, *71*, 3.

[7] C. B. Hübschle, G.M. Sheldrick, B. Dittrich *J. Appl. Crystallogr*. **2011**, *44*, 1281.

[8] D. Kratzert, J.J. Holstein, I. Krossing, *J. Appl. Crystallogr.* **2015**, *48*, 933.

[9] D. Kratzert, I. J. Krossing *Appl. Crystallogr.* **2018**, *51*, 928

[10] A.Thorn, B.Dittrich, G. M. Sheldrick *Acta Crystallogr. Sect. Found. Crystallogr.* **2012**, *68*, 448.

[11] A. L. Spek *Acta Crystallogr. C Struct. Chem.* **2015***, 71*, 9.

[12] A. L. Spek *Acta Crystallogr. D Biol. Crystallogr.* **2009**, *65*, 148.

[13] F. Neese, F. Wennmohs, U. Becker, C. Riplinger, *J. Chem. Phys.* **2020**, *152*, Art. No. L224108 doi.org/10.1063/5.0004608

[14] T. Gasevic, J. B. Stückrath, S. Grimme, M. Bursch, *J. Phys. Chem. A* **2022**, *126*, 3826–3838.

[15] Gaussian 16, Revision B.01, M. J. Frisch, G. W. Trucks, H. B. Schlegel, G. E. Scuseria, M. A. Robb, J. R. Cheeseman, G. Scalmani, V. Barone, G. A. Petersson, H. Nakatsuji, X. Li, M. Caricato, A. V. Marenich, J. Bloino, B. G. Janesko, R. Gomperts, B. Mennucci, H. P. Hratchian, J. V. Ortiz, A. F. Izmaylov, J. L. Sonnenberg, D. Williams-Young, F. Ding, F. Lipparini, F. Egidi, J. Goings, B. Peng, A. Petrone, T. Henderson, D. Ranasinghe, V. G. Zakrzewski, J. Gao, N. Rega, G. Zheng, W. Liang, M. Hada, M. Ehara, K. Toyota, R. Fukuda, J. Hasegawa, M. Ishida, T. Nakajima, Y. Honda, O. Kitao, H. Nakai, T. Vreven, K. Throssell, J. A. Montgomery, Jr., J. E. Peralta, F. Ogliaro, M. J. Bearpark, J. J. Heyd, E. N. Brothers, K. N. Kudin, V. N. Staroverov, T. A. Keith, R. Kobayashi, J. Normand, K. Raghavachari, A. P. Rendell, J. C. Burant, S. S. Iyengar, J. Tomasi, M. Cossi, J. M. Millam, M. Klene, C. Adamo, R. Cammi, J. W. Ochterski, R. L. Martin, K. Morokuma, O. Farkas, J. B. Foresman, and D. J. Fox, Gaussian, Inc., Wallingford CT, 2016.

[16] C. Bannwarth, S. Ehlert, S. Grimme, *J. Chem. Theory Comput.* **2019**, *15*, 1652–1671.

[17] Z. Liu, T. Lu, Q. Chen, *Carbon* **2020**, *165*, 461–467.

[18] T. Lu, *J. Chem. Phys.* **2024**, *161*, 082503.
